# Investigating the therapeutic mechanism of Puerarin in vascular dementia: an integrated approach combining network pharmacology and experimental validation

**DOI:** 10.3389/fphar.2026.1796295

**Published:** 2026-04-29

**Authors:** Hongmei Tang, Yanjiao Li, Yuting Pu, Lingxue Wang, Yunfang Yang, Lu Liu, Xue Bai

**Affiliations:** 1 Department of Neurology, The Affiliated Traditional Chinese Medicine Hospital of Southwest Medical University, Luzhou, Sichuan, China; 2 Chongqing Key Laboratory of Traditional Chinese Medicine for Prevention and Cure of Metabolic Diseases, College of Traditional Chinese Medicine, Chongqing Medical University, Chongqing, China

**Keywords:** molecular docking & dynamics simulations, network pharmacology, Puerarin, TLR4/MyD88/NF-κB, vascular dementia

## Abstract

**Background:**

Vascular dementia (VaD) is the second most common type of dementia after Alzheimer’s disease. Puerarin (PUE) is a natural compound isolated from *Puerariae Lobatae Radix*, plays an important role in treating neurodegenerative and neurovascular diseases. Studies have confirmed that Puerarin can reduce neuroinflammation and protect the blood-brain barrier (BBB). However, the precise mechanism by which PUE treats VaD remains to be elucidated.

**Objective:**

To investigate the therapeutic effect of Puerarin on VaD and its underlying molecular mechanisms.

**Materials and Methods:**

Cognitive performance was assessed using the behavioral testing. Hematoxylin and Eosin staining (H&E) and Nissl staining were employed to assess neuronal morphology in the hippocampus. Additionally, network pharmacology (NP) was used to identify putative bioactive compounds in PUE and candidate targets associated with VaD. Transmission electron microscopy (TEM), enzyme-linked immunosorbent assay (ELISA), quantitative PCR (qPCR), and Western blotting were used for experimental validation. To further interrogate pathway involvement, lipopolysaccharide (LPS), a Toll-like receptor 4 (TLR4) agonist, was administered, followed by repeated behavioral testing and molecular analyses.

**Results:**

Behavioral testing showed that PUE mitigated two-vessel occlusion (2VO)-induced cognitive deficits and reduced hippocampal neuronal injury. NP identified 17 putative active compounds and 100 overlapping targets between PUE and VaD. GO and KEGG enrichment analyses indicated that these targets were enriched in Toll-like receptor and NF-κB signaling, implicating an anti-neuroinflammatory mechanism. Experimental assays showed that PUE reduced TLR4 and myeloid differentiation primary response 88 (MyD88) expression, decreased nuclear factor-kappa-B p65 (NF-κB p65) phosphorylation, and lowered pro-inflammatory mediator levels. Importantly, the administration of the TLR4 agonist LPS effectively counteracted the therapeutic effects of Puerarin.

**Conclusion:**

Puerarin improves learning and cognitive abilities in VaD rats by inhibiting the TLR4/MyD88/NF-κB signaling pathway, thereby attenuating pathological alterations in the hippocampus, protecting the BBB integrity from cerebral ischemic damage, and exerting anti-neuroinflammatory effects.

## Graphical Abstract

**Figure:**
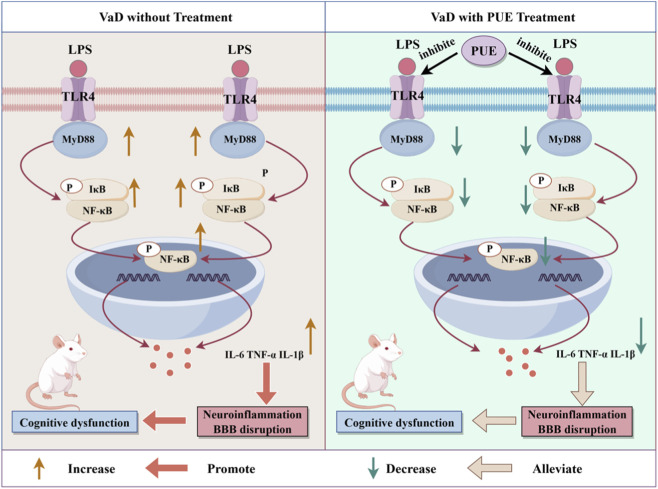

## 1 Introduction

Vascular dementia (VaD) is the second most common type of dementia after Alzheimer’s disease (AD), is estimated to account for over 20% of global dementia cases (Wolters and Ikram, 2019). VaD impedes daily living and social activities of the sufferer, posing heavy economic and psychological burdens on the family and society (Yang et al., 2017). Current therapeutic strategies predominantly rely on the off–label use of cognitive enhancers such as donepezil, memantine, and piracetam. However, none of these pharmacotherapeutics have received FDA approval for VaD–specific use (Wang et al., 2025). Therefore, developing novel therapeutic strategies for VaD, grounded in mechanistic studies, is of great importance.

The TLR4/MyD88/NF-κB signaling pathway, a canonical inflammatory cascade, plays a crucial role in neuroinflammation induced by cerebral ischemia. Upon neuronal damage and death, damage-associated molecular patterns ((DAMPs) are released and can be recognized by Toll-like receptor 4 (TLR4) on the surface of microglia. Subsequently, TLR4 recruits the key adaptor protein myeloid differentiation primary response 88 (MyD88), which drives the activation of the transforming growth factor-β-activated kinase 1 (TAK1) protein kinase complex. This leads to the phosphorylation and proteasomal degradation of the NF-κB inhibitory protein IκBα, allowing NF-κB to migrate into the nucleus and induce the expression of pro-inflammatory genes (Kumar, 2019; Lim and Staudt, 2013). Previous studies have demonstrated that inhibiting the TLR4/MyD88/NF-κB pathway can effectively ameliorate cognitive impairment in VaD rats (Bu et al., 2022; Zhu K. et al., 2021). Therefore, alleviating neuroinflammation by inhibiting this pathway may be a potential strategy for treating VaD.

Traditional Chinese medicine (TCM) has attracted growing attention owing to its favorable safety profile and fewer adverse reactions. Puerarin (PUE) is a natural compound isolated from *Puerariae Lobatae Radix* and an isoflavone compound, has been shown to be neuroprotective in different neurological pathologies such as AD, Parkinson’s disease, brain ischemia and bilateral common carotid artery occlusion (BCCAO)-induced VaD (Yu et al., 2022). Clinical investigations have revealed that PUE injection can effectively improve hemorheological parameters, increase cerebral blood flow, inhibit microthrombus formation, and show good therapeutic efficacy in the prevention and treatment of ischemic stroke (Liu et al., 2016). Additionally, PUE has protective effects against cognitive impairment, oxidative stress, and cellular apoptosis (Ren and Qu, 2023; Zhu T. et al., 2021). However, the mechanism by which PUE exerts neuroprotective effects has not been fully elucidated and warrants further investigation. Therefore, based on existing databases, we employed network pharmacology (NP) and molecular docking techniques to predict key targets and pathways and to experimentally validate the proposed mechanism by which PUE improves cognitive outcomes.

With the rapid development of bioinformatics, NP has become a useful tool for dissecting multicomponent therapeutics and their system-level mechanisms. It is widely used to discover potential active ingredients in traditional Chinese medicine and predict their molecular-level pharmacological targets (Zhao et al., 2023; Noor et al., 2022). In addition, molecular docking is a method based on computer modelling of structures. It is widely used to assist in the discovery of new medicines (Pinzi and Rastelli, 2019). This study hypothesize that Puerarin attenuates VaD by modulating the TLR4/MyD88/NF-κB signaling pathway, thereby preserving blood-brain barrier (BBB) integrity and alleviating neuronal injury. To investigate the therapeutic effect of Puerarin on VaD and its underlying molecular mechanisms, network pharmacology predictions were performed and subsequently validated through *in vivo* experiments.

## 2 Materials and methods

### 2.1 Network pharmacology analysis

#### 2.1.1 Collecting targets of PUE and vascular dementia

The targets of PUE were collected using the TCMSP (http://tcmspw.com/tcmsp.php) with “Puerarin” as the keyword. VaD-related genes were identified from the GeneCards (https://www.genecards.org/), OMIM (https://omim.org/), and TTD (http://db.idrblab.net/ttd/) databases using “Vascular Dementia” as the keyword. The retrieved targets were combined, deduplicated, and designated as VaD-related targets. A Venn diagram was generated to visualize the common targets between PUE and VaD using the Venn Diagrams package (Version 1.11). Subsequently, a “PUE-VaD-Target” network was constructed using Cytoscape 3.7.1 software.

#### 2.1.2 Constructing the protein-protein interaction network

The common targets were uploaded to the STRING database (https://cn.string-db.org/) to construct a protein-protein interaction (PPI) network, with the organism set to “*Homo sapiens*” and aconfidence score ≥0.4. The PPI network data were then imported into Cytoscape to identify core proteins based on their degree values. These proteins were further analyzed using the molecular complex detection (MCODE) plug-in.

#### 2.1.3 Gene ontology (GO) and kyoto encyclopedia of genes and genomes (KEGG) pathway enrichment analyses

The Metascape database (https://metascape.org/) was utilized to perform GO and KEGG pathway enrichment analyses. GO enrichment analysis included biological processes (BP), cellular components (CC), and molecular functions (MF). For KEGG pathway analysis, terms with a *p*-value ≤0.05 were considered significant, and the top 19 pathways based on count values were selected. The results were visualized using the Bioinformatics platform (http://www.bioinformatics.com.cn/) to generate bubble charts and histograms.

#### 2.1.4 Molecular docking

Molecular docking was performed between the PUE with the core targets from the PPI network. The two-dimensional structures of the small-molecule ligands were obtained from the PubChem database (https://pubchem.ncbi.nlm.nih.gov). The three-dimensional structures of the target proteins were retrieved from the Protein Data Bank (https://www.rcsb.org/). Water molecules and original ligands were removed from the protein structures using PyMOL software. The proteins were then prepared by adding hydrogen atoms and defining the active pockets using AutoDock Tools. Molecular docking was executed using AutoDock Vina, and the results were visualized with PyMOL.

### 2.2 Drugs and animals

Puerarin (Pur, >98% HPLC purity) was provided by Aladdin (Shanghai, China, Cat. No.: P111270).

The doses of Puerarin (100, 200, and 400 mg/kg) were selected based on previous study (Zhang et al., 2026) demonstrating its inhibition of the TLR4/NF-κB signaling pathway in a dose-dependent manner.

A total of 70 male Sprague-Dawley (SD) rats (8 weeks old, 250–300 g) were obtained from the Experimental Animal Center of Southwest Medical University. All rats were housed under a 12-h light/dark cycle at a temperature of 22 °C ± 2 °C and 65% ± 5% relative humidity. All experimental procedures were strictly performed in accordance with the ethical guidelines and were approved by the Institutional Animal Care and Use Committee of Chongqing Medical University (Approval No.: IACUC-CQMU-2424-0733). All animals were euthanized by an intraperitoneal injection of an overdose of sodium pentobarbital (150 mg/kg body weight) at the end of the experiment. Pentobarbital sodium (Cat. No.: P12149, purity >99%) was purchased from Serva (Heidelberg, Germany).

### 2.3 Establishment of the VaD rat model and grouping

The VaD model was established by permanent two-vessel occlusion (2VO) (Eklöf and SIESJö, 1972; Farkas et al., 2007; Venkat et al., 2015). In the sham group, the common carotid arteries were isolated but not ligated. Penicillin (80,000 IU/d Yeasen, Shanghai, China, No.:6026ES76) was administered intramuscularly to the rats for 3 days post-surgery. After a 30-day recovery period, the rats were randomly divided into the following groups (*n* = 10 per group) using a random number table: Sham group, Model group (2VO), Low-dose PUE group (2VO + PUE 100 mg/kg/d), Medium-dose PUE group (2VO + PUE 200 mg/kg/d), High-dose PUE group (2VO + PUE 400 mg/kg/d), LPS group (2VO + LPS), and LPS + PUE group (2VO + LPS + PUE 400 mg/kg/d). The LPS and LPS + PUE groups received intraperitoneal injections of LPS (Ahmadi et al., 2022) (0.5 mg/kg, Solarbio, Beijing, China, Cat. No.: L8880) 1 day before the start of gastric gavage. The Model and Sham groups were administered 3 mL of normal saline (Biyuntian biological technology, Shanghai, China, Cat. No.: ST341) by gavage. Oral gavage was performed daily in the morning for 30 consecutive days. The Morris Water Maze (MWM) test, Y-maze test, and Novel Object Recognition (NOR) test were conducted after the final gavage to evaluate cognitive function. A total of 10 rats were included in each group. During the Morris water maze training phase, 2 rats per group failed to complete the acquisition trials and were excluded from subsequent behavioral testing. Consequently, behavioral data were obtained from 8 rats per group, hippocampal tissues from 5 rats per group were collected for qPCR and Western blot analysis. An additional 3 rats per group were used for histopathological examination.

### 2.4 Morris Water Maze test

The MWM test, used to assess spatial learning and memory, consisted of a spatial acquisition phase and a spatial probe test (Morris, 1984). After each trial, the apparatus (water maze pool, Y-maze arms, or open field arena) was thoroughly cleaned with 70% ethanol solution and dried completely to remove any olfactory traces left by the previous animal. This procedure was strictly followed to minimize olfactory cues and ensure that each animal’s performance was based solely on spatial or visual cues rather than scent marks The spatial acquisition test was conducted over 5 consecutive days (days 1–5) to evaluate spatial learning ability. The spatial probe test was performed on day 6 to assess spatial memory retention. All data were recorded and analyzed using a video tracking system. The results are expressed as mean escape latency (seconds) to find the hidden platform, time spent in the target quadrant (seconds) and number of platform crossings.

### 2.5 Y-maze test

The Y-maze consisted of three black, opaque arms arranged at 120° angles. Rats were acclimated to the testing room for at least 24 h before testing. Each rat was placed at the end of one designated arm and allowed to explore freely for 8 min. An entry into all three arms in consecutive order was recorded as a spontaneous alternation. After each trial, all apparatuses were cleaned with 70% ethanol between trials to eliminate odor cues to remove any olfactory traces left. The total number of arm entries and the number of spontaneous alternations were recorded. The spontaneous alternation percentage was calculated as: (alternations/[total entries −2]) × 100% (Kraeuter et al., 2019; Song et al., 2022).

### 2.6 Novel object recognition test

Rats were acclimated to the testing room for at least 24 h before testing. On day 1 (familiarization phase), two identical objects (A and B) were placed in the bottom left and top right corners of the box. Each rat was placed in the box facing away from the objects and allowed to explore for 5 min. On day 2 (test phase), object B was replaced with a novel object C. Each rat was again placed in the box and allowed to explore for 5 min. After each trial, the device and artefacts were thoroughly cleaned with 70% ethanol to eliminate odor cues to remove any olfactory traces left. The interaction times with the familiar object (A) and the novel object (C) on day 2 were recorded. The discrimination ratio was calculated as: [Time exploring novel object/(Time exploring novel object + Time exploring familiar object)] × 100% (Ennaceur and DELACOUR, 1988; Tian et al., 2023).

### 2.7 Specimen collection

#### 2.7.1 Serum collection

Blood samples were collected from the abdominal aorta under deep anesthesia (1% pentobarbital sodium, 40 mg/kg, i.p.) immediately after behavioral testing. Pentobarbital sodium (Cat. No.: P12149, purity >99%) was purchased from Serva (Heidelberg, Germany). Blood was allowed to clot at room temperature for 30 min, then centrifuged at 3000 rpm for 15 min at 4 °C. The supernatant (serum) was carefully collected and stored at −80 °C until further analysis.

#### 2.7.2 Fresh hippocampal tissue collection

After blood collection, rats were rapidly decapitated. The whole brain was quickly removed from the skull and placed on an ice-cold plate. The hippocampus was bilaterally dissected according to a standard rodent brain dissection protoco (Aboghazleh et al., 2024). The procedure was performed rapidly (within 3-5 min post-euthanasia) on ice to minimize tissue degradation. Fresh hippocampal tissues were immediately frozen in liquid nitrogen and stored at −80 °C for subsequent Western blot and PCR analyses.

#### 2.7.3 Pathological tissue collection

For histopathological examination, rats were transcardially perfused with 0.1 M phosphate-buffered saline (PBS, pH 7.4, Thermo Fisher Scientific, Waltham, MA, USA, Cat. No.: 70011069) followed by 4% paraformaldehyde in 0.1 M PBS (Biotium, Fremont, CA, USA, Cat. No.:22023). The brains were carefully removed and post-fixed in the same fixative for 24 h at 4 °C. After fixation, brains were embedded in paraffin (Paraplast® X-tra, Leica Biosystems, Wetzlar, Germany, Cat. No.: 39503002), and coronal sections (5 μm thickness) were cut through the dorsal hippocampus (bregma −2.5 to −4.0 mm) using a microtome (model M530; MEDITE Medical GmbH, Burgdorf, Germany). Sections were mounted on slides for subsequent HE, Nissl, and immunohistochemical staining. For TEM analysis, the hippocampus was dissected immediately upon removal of the brain following cardiac perfusion on an ice-cold platform. Under a stereomicroscope, the hippocampal cornu ammonis 1 (CA1) region was identified and cut into small blocks (approximately 1 mm^3^) using a sharp razor blade. Tissue blocks were immediately immersed in 2.5% glutaraldehyde fixative (Electron microscopy grade, 2.5% in 0.1 M PBS; Macklin, via Beijing Lanyi Chemical Co., Ltd., Beijing, China, Cat. No.: G708101-100 mL) and stored at 4 °C. All procedures from perfusion to tissue immersion in fixative were completed within 3 min to preserve ultrastructural integrity.

### 2.8 Transmission electron microscopy (TEM)

Hippocampal tissues were fixed with 2.5% glutaraldehyde (Electron microscopy grade, 2.5% in 0.1 M PBS; Macklin, via Beijing Lanyi Chemical Co., Ltd., Beijing, China, Cat. No.: G708101-100 mL) and then with 1% osmium tetroxide (Thermo Fisher Scientific, Waltham, MA, USA, Cat. No.: ALF-045384-AB). Following dehydration through a graded acetone series (HPLC grade, ≥99.9%, Sigma-Aldrich, St. Louis, MO, USA, Cat. No.: 270725), the tissues were embedded in EPON812 resin (Embed 812, Electron Microscopy Sciences, Hatfield, PA, USA, Cat. No.: 100503-876). Ultrathin sections (80 nm) were cut using an ultramicrotome (Leica EM UC7; Leica, Nussloch, Germany), stained with uranyl acetate (4% aqueous solution, EPREDIA, via VWR, Radnor, PA, USA, Cat. No.: 102092-288) for 10-15 min and lead citrate (≥99%, Electron Microscopy Sciences, Hatfield, PA, USA, Cat. No.: 100504-070) for 1-2 min, and finally observed and photographed under a transmission electron microscope (JEM-1400FLASH, JEOL, Tokyo, Japan). The results are expressed descriptively, focusing on the ultrastructural features of the BBB.

### 2.9 Histological staining

Hippocampal tissues were fixed in formaldehyde and embedded in paraffin using standard protocols. The paraffin blocks were sectioned into 4 μm slices. Sections were stained with Hematoxylin and Eosin staining (H&E) or Nissl staining according to standard protocols (Cardiff et al., 2014; De Prisco et al., 2022). The H&E staining kit was purchased from Solarbio (Beijing, China; Cat. No.: G1120). The Nissl staining kit was purchased from KEMOBio (Wenzhou, Zhejiang, China; Cat. No.: KMR021308). The stained sections were examined and imaged under a light microscope (Leica DM4000B; Leica, Solms, Germany) at ×200 and ×400 magnification. The results are expressed as the number of surviving neurons and Nissl bodies per 1 mm length in the CA1 region of the hippocampus.

### 2.10 Real-time quantitative polymerase chain reaction (qPCR)

Total RNA was extracted from hippocampal tissues stored at −80 °C using TRIzol reagent (Invitrogen, Shanghai, China, Cat. No.: 15596026CN). The concentration and purity of RNA were assessed using a NanoDrop 2000 spectrophotometer (ND-2000; Thermo Fisher Scientific, Waltham, MA, USA). Samples with A260/A280 ratios between 1.9 and 2.1 and A260/A230 ratios greater than 2.0 were considered acceptable for further analysis, indicating pure RNA free from protein and organic solvent contamination (Fleige and Pfaffl, 2006; Bustin et al., 2009). Only RNA samples meeting these purity criteria were used for subsequent qPCR. For cDNA synthesis, 1 μg of total RNA was reverse transcribed using a PrimeScript™ RT reagent kit (AG Biotechnology Co., Ltd, Shanghai, China, Cat. No.: AG12503) following the manufacturer’s protocol. Quantitative real-time PCR was performed on a fluorescence real-time PCR system (LightCycler® 480 II; Roche, Switzerland) using SYBR Green Master Mix (PowerTrack™ SYBR Green Master Mix, Applied Biosystems, Thermo Fisher Scientific, Waltham, MA, USA, Cat. No.: A46110). A melting curve analysis was performed to confirm the specificity of the amplification. The results are expressed as relative mRNA expression levels normalized to the internal control gene (GAPDH), calculated using the 2^−ΔΔCt^ method (Livak and Schmittgen, 2001; Schmittgen and Livak, 2008), and presented as fold change relative to the control group. The primer sequences are listed in Supplementary Table 1 (designed and synthesized by Shanghai Bioengineering Co., Ltd., Shanghai, China).

### 2.11 Western blotting

Hippocampal tissues were homogenized in RIPA lysis buffer (Biyuntian biological technology, Shanghai, China, Cat. No.: P0013E) containing protease inhibitor, phosphatase inhibitor, and PMSF on ice to extract total protein. The protein concentration was determined using a BCA assay (Biyuntian biological technology, Shanghai, China, Cat. No.: P0010). Then, 30 μg of total protein per sample was separated by SDS-PAGE (Biyuntian biological technology, Shanghai, China, Cat. No.: P0012AC) and transferred onto PVDF membranes (Merck Millipore, Darmstadt, Germany, Cat. No.: IPVH00010). The membranes were blocked with 5% non-fat milk (Biyuntian biological technology, Shanghai, China, Cat. No.: P0216-300g) for 1 h at room temperature and subsequently incubated with specific primary antibodies overnight at 4 °C. The primary antibodies used were as follows: Zonula occludens-1 (ZO-1) (1:5000, Proteintech, Wuhan, Hubei, China, Cat. No.: RMX00002), Occludin (1:5000, Proteintech, Wuhan, Hubei, China, Cat. No.: 66378-1-Ig), Claudin-5 (1:5000, Proteintech, Wuhan, Hubei, China, Cat. No.: 29767-1-AP), TLR4 (1:100, SANTA, Dallas, TX, USA, No.: sc-293072), NF-κB p65 (1:10000, Proteintech, Wuhan, Hubei, China, Cat. No.: 10745-1-AP), P-NF-κB p65 (1:1000, abmart, Shanghai, China, No.: AB_2936854), Interleukin-6 (IL-6) (1:1000, abmart, Shanghai, China, No.: TD6087), Tumor necrosis factor-alpha (TNF-α) (1:1000, abmart, Shanghai, China, No.: PA2174), Interleukin-1β (IL-1β) (1:1000, Zenbio, Chengdu, Sichuan, China, Cat. No.: 516288) and β-actin (1:1000, Proteintech, Wuhan, Hubei, China, Cat. No.: 20536-1-AP). After incubation, the membranes were washed three times with TBST (Biyuntian biological technology, Shanghai, China, Cat. No.: ST673-500 mL) and incubated with an HRP-conjugated secondary antibody (1:5000, CST, Danvers, MA, USA, anti-rabbit IgG, Cat. No.: 7074; anti-mouse IgG, Cat. No.: 7076) for 1 h at room temperature. Protein bands were visualized using an ECL chemiluminescence substrate (Yeasen, Shanghai, China, No.:36222ES60), detected with a Chemi-doc XRS gel documentation system (Bio-Rad, Laboratories, Hercules, CA, USA) and quantified by densitometry using ImageJ software (National Institutes of Health, Bethesda, MD, USA). The results are expressed as the relative protein expression level (target protein/β-actin ratio).

### 2.12 Immunohistochemistry

Sections were deparaffinized and rehydrated. Immunohistochemistry was performed according to instructions. Briefly, the sections were pretreated with sodium citrate buffer (Boster Biological Technology, Pleasanton, CA, USA, Cat. No.: AR0024) at 98 °C for 30 min for antigen retrieval, and then the endogenous peroxidase activity was blocked with SignalStain® Peroxidase Blocking Reagent (CST, Danvers, MA, USA, Cat. No.: 15039S) for 10 min and then with BSA (Biyuntian biological technology, Shanghai, China, Cat. No.: ST023) for 30 min, incubated with the primary P-NF-κB p65 (1:300, abmart, Shanghai, China, No.: TP56372) overnight at 4 °C, and finally with goat anti-mouse biotinylated antibody (1:200, CST, Danvers, MA, USA, No.: 14709S) for 30 min at room temperature. For chromogenic detection, sections were treated with DAB Chromogen Kit (Biocare Medical, Pacheco, CA, USA, Cat. No.: DB801), followed by hematoxylin (Sigma-Aldrich, St. Louis, MO, USA, Cat. No.: HHS16) counterstaining.

### 2.13 Enzyme-linked immunosorbent assay (ELISA)

The serum levels o in rats were determined using commercial ELISA kits (Aifang Bio, Changsha, Hunan, China, No.: AF03056-A for TNF-α, AF03066-A for IL-6, AF02923-A for IL-1β) strictly according to the manufacturer’s instructions. The absorbance was measured at 450 nm. The results are expressed as the concentration of the target protein (pg/mL), calculated by interpolation from the standard curve generated with known concentrations of the standard.

### 2.14 Statistical analysis

**FIGURE 1:**
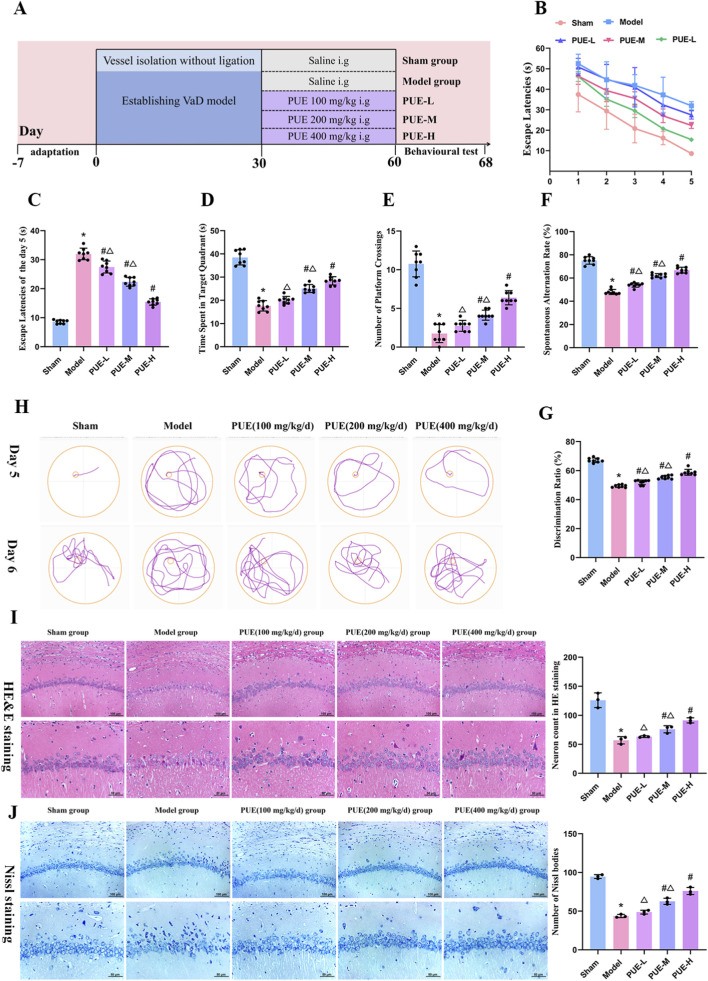
PUE ameliorates memory impairment and hippocampal pathological damage in VaD rats. **(A)** Timeline of experiments; **(B)** Escape latency of 1-5 days. Data are presented as mean ± SEM to illustrate the learning curves of each group. No statistical comparisons were performed on these training-phase data; **(C)** Escape latency of the Day 5; **(D)** The time spent in target quadrant of the Day 6; **(E)** The number of platform crossings of the Day 6; **(F)** Y-maze test of spontaneous alternation rate; **(G)** The novel object recognition test of discrimination index; **(H)** Representative trajectories from Day 5 and Day 6; **(I)** HE staining results; **(J)** Nissl staining results. Data are presented as mean ± SEM (B–G: *n* = 8; I–J: *n* = 3). Significant differences compared with Sham group were designated as *P < 0.05, with Model group as ^#^ *P* < 0.05 and with PUE-H group with as ^△^ *P* < 0.05.

Data are presented as the mean ± standard error of the mean (SEM). Statistical analysis was performed using GraphPad Prism 8.4.3 (GraphPad Software, La Jolla, CA, USA). The normality of the data was assessed with the Shapiro-Wilk test. Statistical significance was determined by one-way analysis of variance (ANOVA) followed by Tukey’s post-hoc test for multiple comparisons. The training phase data (escape latency across 5 days) shown in Figures 1B, 3B are presented for descriptive purposes only to illustrate the learning trends of each group; no statistical comparisons were performed on these longitudinal data. *P* < 0.05 was considered statistically significant.

## 3 Results

### 3.1 PUE treatment improved learning and memory functions in VaD rats

The timeline of experiments is shown in Figure 1A. In the Morris water maze test, during the 5-day acquisition phase, the model group exhibited significantly longer escape latency compared to the sham group. Treatment with various doses of PUE markedly reduced the escape latency in VaD rats (Figures 1B,C). In the probe trial, PUE treatment increased the time spent in the target quadrant and the number of platform crossings in VaD rats (Figures 1D,E). The trajectory maps on days 5 and 6 of the Morris water maze test are shown in Figure 1H. In the Y-mase test, the model group showed a significantly reduced spontaneous alternation rate compared to the sham group, whereas PUE-treated VaD rats showed higher rates of spontaneous alternation (Figure 1F). The novel object recognition test revealed that the sham group exhibited a clear preference for novel objects, while the model group showed a significantly decreased discrimination index. In contrast, PUE-treated VaD rats demonstrated higher recognition indices (Figure 1G).

### 3.2 PUE treatment alleviates neuronal injury and loss in the hippocampus of VaD rats

In the hippocampal CA1 region, H&E staining revealed that neurons in the sham group were densely packed with intact cellular structures. In contrast, the model group exhibited reduced neuronal density, incomplete cellular structures, and instances of nuclear condensation and necrosis. Treatment with PUE significantly improved neuronal morphology in the hippocampal CA1 region of VaD rats, resulting in densely packed neurons and reduced necrosis (Figure 1I). Nissl staining further revealed that neurons in the sham group exhibited round nuclei, clear membrane structures, and abundant, uniformly distributed Nissl bodies. The model group showed a significant decrease in Nissl bodies, indicating neuronal damage. Following PUE treatment, the number of Nissl bodies in the hippocampal CA1 region of VaD rats was markedly increased (Figure 1J). Quantitative analysis revealed that the effects were more pronounced in the high-dose PUE groups.

### 3.3 Identification of potential mechanisms of PUE

**FIGURE 2:**
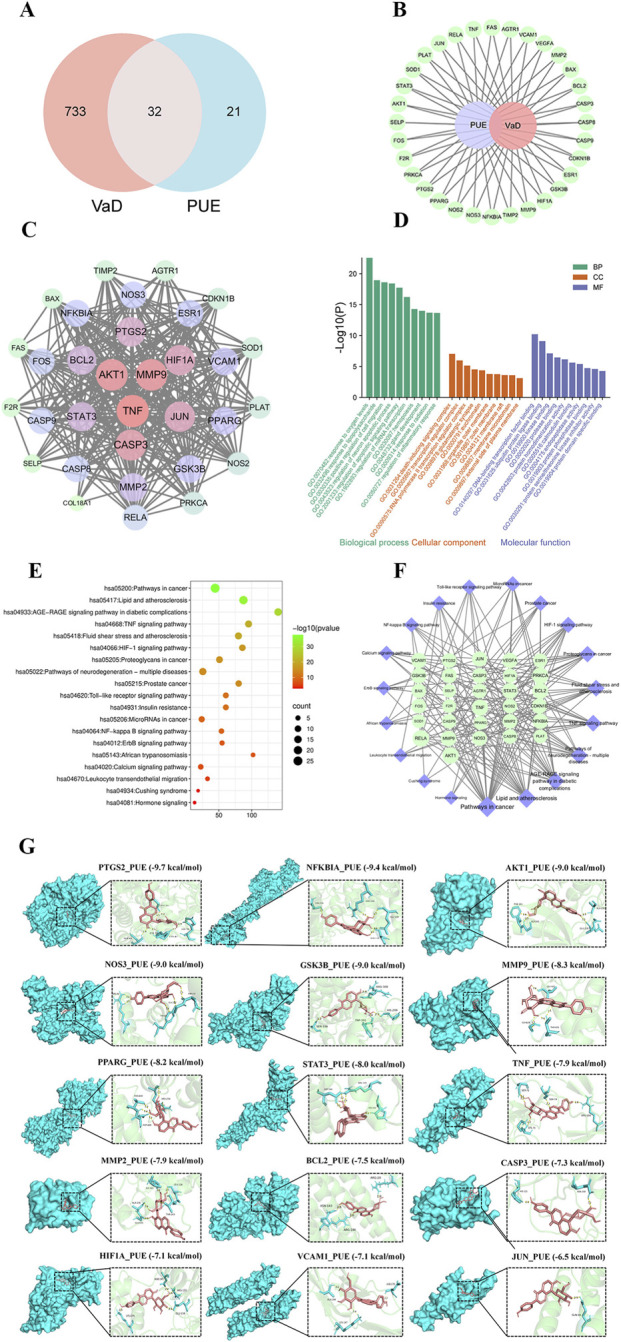
Network pharmacology results. **(A)** Venn diagram of VaD and PUE; **(B)** “PUE-VaD-Target network”. The dark green represent target genes; **(C)** PPI network of the common target genes. The nodes represent target genes; the edges represent target genes-target genes associations; **(D)** GO analysis on common targets (top 10). Green represents biological process, red represent cellular component, and purple represent molecular function; **(E)** “KEGG pathway-Targets network”; **(F)** KEGG pathway exploration of common targets (top 20). The size of the bubble indicated the pathway count, while colors indicated the significance of *P*-value. **(G)** Molecular docking results. Active ingredient shown as pink color, Hydrogen bonds are shown as yellow lines.

A total of 53 potential targets associated with PUE were retrieved from TCMSP after name conversion and deduplication. Meanwhile, 765 VaD-related targets were collected from the TTD, OMIM, and GeneCards databases. Comparison of PUE targets with VaD targets yielded 32 common genes as potential therapeutic targets for PUE in VaD (Figure 2A).

A “PUE-VaD-Target’ network was constructed using Cytoscape 3.7.1 software (Figure 2B). PPI network analysis of the 32 potential targets identified the top 9 key targets based on degree values: *AKT1, TNF, MMP9, CASP3, JUN, HIF1A, PTGS2, BCL2* and *STAT*3 (Figure 2C).

GO enrichment analysis revealed significant terms, including responses to lipopolysaccharide, regulation of neuron apoptotic process and regulation of inflammatory response. The involved cellular components primarily included various membrane structures and the cytoplasm (Figure 2D). KEGG pathway enrichment analysis identified 131 significantly enriched pathways (*P* < 0.05). The top 19 pathways are displayed in Figures 2E,F. Key pathways relevant to VaD included the TNF signaling pathway, Toll-like receptor signaling pathway and NF-κB signaling pathway.

### 3.4 Molecular docking results

Molecular docking analysis revealed binding energies between PUE and the top 15 targets (ranked by degree). The binding energies are shown in Figure 2G.

### 3.5 PUE protects the BBB via the TLR4/MyD88/NF-κB signaling pathway

**FIGURE 3:**
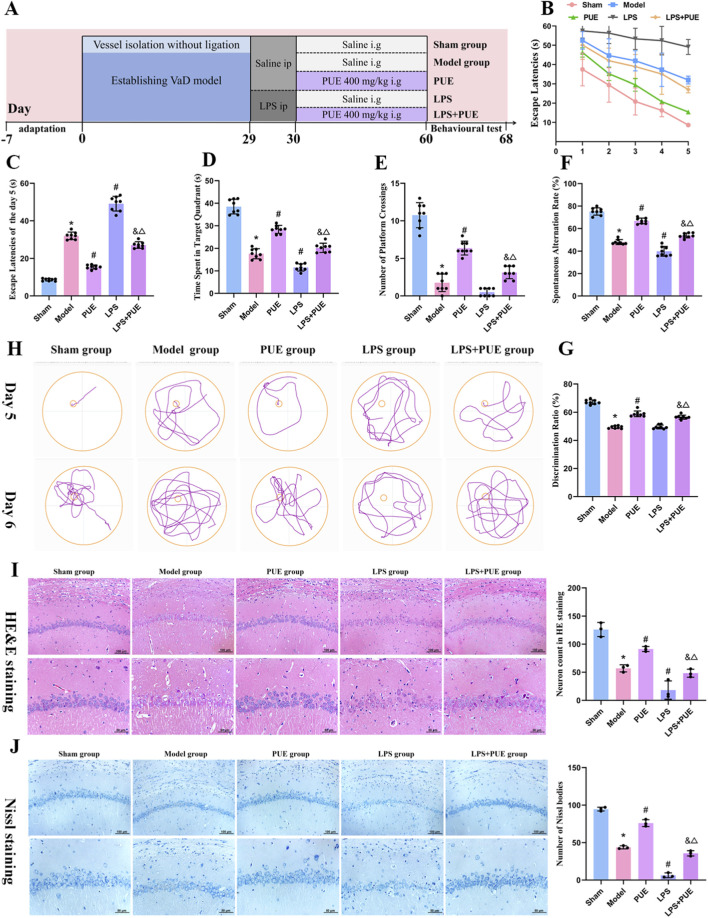
LPS-Eliminated PUE’s ameliorative effects on memory impairment and hippocampal pathological damage in VaD rats. **(A)** Timeline of experiments; **(B)** Escape latency of 1-5 days; **(C)** Escape latency of the Day 5. Data are presented as mean ± SEM to illustrate the learning curves of each group. No statistical comparisons were performed on these training-phase data; **(D)** The time spent in target quadrant of the Day 6; **(E)** The number of platform crossings of the Day 6; **(F)** Y-maze test of spontaneous alternation rate; **(G)** The novel object recognition test of discrimination index; **(H)** Representative trajectories from Day 5 and Day 6; **(I)** HE staining results; **(J)** Nissl staining results. Data are presented as mean ± SEM (B–G: *n* = 8; I–J: *n* = 3). Significant differences compared with Sham group were designated as *P < 0.05, with model group as ^#^ *P* < 0.05, with PUE group as ^&^ *P* < 0.05 and with LPS group as ^△^ *P* < 0.05.

The behavioral and histopathological benefits of PUE in VaD rats were consistent with the results described above. Notably, these effects were attenuated by co-administration of LPS, a TLR4 agonist. As shown in Figure 3, LPS stimulation further exacerbated learning and memory impairments and pathological damage in the hippocampal CA1 region of VaD rats, while PUE therapy partially reversed these alterations.

**FIGURE 4:**
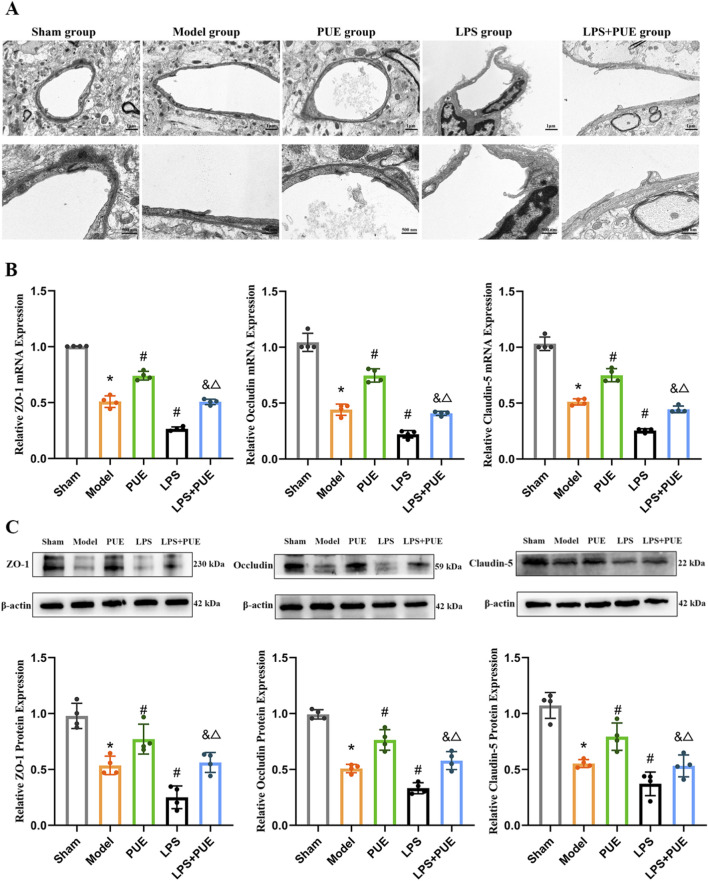
PUE protects the BBB Structure in VaD Rats. **(A)** Transmission electron microscope result (12000×,30000×); **(B)** mRNA levels of ZO-1, Occludin and clauidn-5; **(C)** Protein levels of ZO-1, Occludin and clauidn-5. Data are presented as mean ± SEM (*n* = 4). Significant differences compared with Sham group were designated as *P < 0.05, with Model group as ^#^ *P* < 0.05; with PUE group as ^&^ *P* < 0.05 and with LPS group as ^△^ *P* < 0.05.

As shown in Figure 4A, TEM revealed intact BBB structures with normal mitochondria and tight junctions in the sham group. In contrast, the model group showed disrupted tight junctions, a pathology that was exacerbated in VaD rats receiving LPS injection. PUE treatment effectively attenuated this damage. Consistent with these findings, the levels of ZO-1, Occludin, and Claudin-5 were significantly lower in the VaD group than in the Sham group (Figures 4B,C). This decrease was further aggravated by LPS administration. Importantly, PUE treatment reversed this downregulation of tight junction proteins.

**FIGURE 5:**
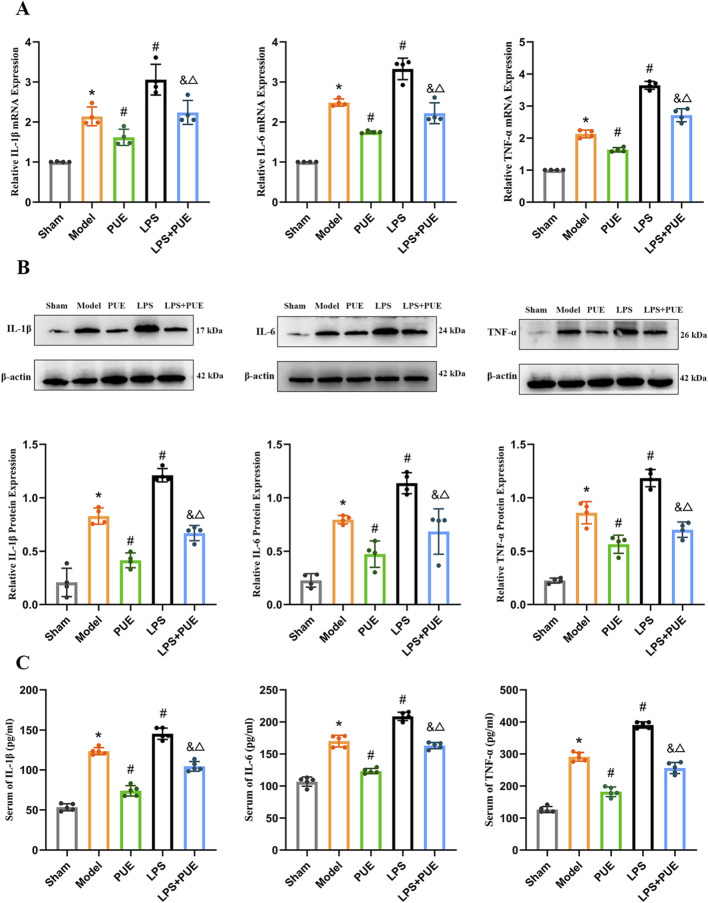
PUE reduced the inflammatory factors in vascular dementia rats. **(A)** mRNA levels of IL-1β,IL-6 and TNF-α (*n* = 4); **(B)** Protein levels of IL-1β,IL-6 and TNF-α (*n* = 4); **(C)** Expression of IL-1β,IL-6 and TNF-α in serum of rats (*n* = 5). Data are presented as mean ± SEM. Significant differences compared with Sham group were designated as *P < 0.05, with Model group as ^#^ *P* < 0.05, with PUE group as ^&^ *P* < 0.05 and with LPS group as ^△^ *P* < 0.05.

The mRNA and protein levels of IL1β, IL-6 and TNF-α were significantly elevated in VaD rats compared to the sham group. PUE treatment strongly suppressed these increases (Figures 5A,B). Serum levels of IL1β, IL-6, and TNF-α were also elevated in the model group and reversed by PUE (Figure 5C).

**FIGURE 6:**
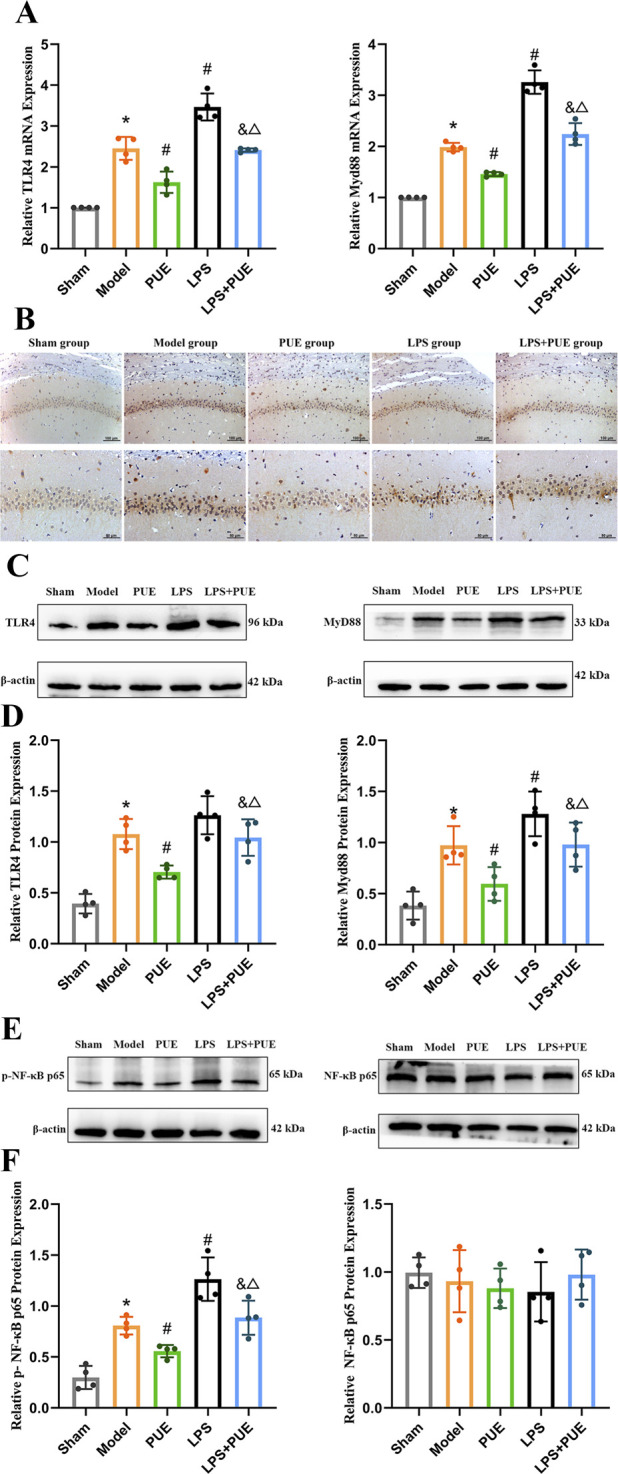
PUE inhibits the TLR4/MyD88/NF-κB signaling pathway in VaD rats. **(A)** mRNA levels of TLR4 and MyD88(*n* = 4); **(B)** Immunohistochemical staining of P- NF-κB P65 in hippocampus (200×, 400×); **(C–F)** Protein levels of TLR4, Myd88, P- NF-κB P65, NF-κB P65 (*n* = 4); Data are presented as mean ± SEM. Significant differences compared with Sham group were designated as *P < 0.05, with Model group as ^#^ *P* < 0.05, with PUE group as ^&^ *P* < 0.05 and with LPS group as ^△^ *P* < 0.05.

TLR4, MyD88, and phosphorylated NF-κB p65 (p-p65) levels were significantly increased in VaD rats compared with the sham group. PUE treatment reduced these increases, whereas total p65 levels did not differ significantly among groups (Figure 6).

The therapeutic effects of PUE were abolished by the TLR4 agonist LPS. As shown in Figures 5, 6, LPS administration increased the levels of TLR4, MyD88, p-p65, and inflammatory factors compared to the model group. However, co-treatment with PUE and LPS counteracted this increase, leading to a decrease in these markers.

## 4 Discussion

The increasing incidence of Vascular Dementia has intensified research focus on its underlying mechanisms, among which long-term cerebral hypoperfusion is a primary cause. Inadequate blood flow activates a cascade of cellular and molecular reactions, leading to a breakdown of the blood-brain barrier and neurodegeneration (Rajeev et al., 2022; Muneer et al., 2017). Chronic cerebral hypoperfusion with reduced cerebral blood flow (CBF) can result in brain damage through oxidative stress, apoptosis, neuroinflammation, and abnormal energy metabolisms (Zou et al., 2023). With a venerable clinical history, Puerarin has been extensively applied in treating cerebrovascular diseases. Study (Li et al., 2025) showed that Puerarin treatment significantly reduced neuronal apoptosis and provided significant cognitive and memory function recovery. Another study (Luo et al., 2025) demonstrated that Puerarin has significant therapeutic potential in treating Chronic cerebral hypoperfusion (CCH)-related cognitive impairments and white matter injury (WMI) by modulating CD36-mediated microglial myelin clearance through the IL-10/STAT3 pathway. However, its potential role and underlying mechanisms in VaD-associated inflammation remained unexplored. The present study aimed to investigate, for the first time, the anti-inflammatory effects of Puerarin and the involved mechanisms using a combination of network pharmacology and experimental validation. The finding revealed that Puerarin treatment alleviated cognitive impairment, attenuated pathological alterations in the CA1 area, and protected the BBB integrity. Mechanistically, these effects are attributed to the inhibition of the TLR4/MyD88/NF-κB inflammatory signaling pathway, a mechanism not previously reported in the context of VaD.

Cognitive deficits in VaD are closely associated with hippocampal neuronal integrity and function. The observed preservation of neuronal structure and reduction in neurodegenerative changes following Puerarin treatment is consistent with its well-documented neuroprotective properties. The BBB is crucial for protecting the central nervous system from potentially harmful signals. Furthermore, BBB disruption is recognized as a critical pathological event in VaD progression (Zhang et al., 2024). The ability of Puerarin to reverse BBB damage and upregulate tight junction proteins observed in this study corroborates earlier work suggesting that Puerarin maintains vascular integrity through multiple mechanisms (Chen et al., 2025). Interestingly, this therapeutic effect was reversed following the introduction of the TLR4 agonist LPS.

To explore the molecular basis of these effects, network pharmacology analysis was employed. This approach identified multiple key targets, including *AKT1, TNF, MMP9, CASP3, JUN, HIF1A,* and *PTGS2*, which are known to be involved in inflammatory responses and neuronal survival. GO and KEGG enrichment analyses further predicted that Puerarin may modulate biological processes related to inflammatory response regulation and influence inflammation-related pathways such as TNF, Toll-like receptor, and NF-κB signaling. These predictions are consistent with the established multi-target, multi-pathway characteristics of natural compounds (Hopkins, 2008). The subsequent molecular docking analysis confirmed favorable binding affinities between Puerarin and the predicted core targets. Typically, binding energies <−4.25 kcal/mol indicate potential binding, whereas values <−5.0 and <−7.0 kcal/mol suggest relatively strong and excellent affinity, respectively (Li et al., 2022).

Toll-like receptors are transmembrane pattern recognition receptors responsible for pathogen recognition and initiating innate immunity. TLR4, the most extensively studied, is a recognized therapeutic target for cerebral ischemia (Han et al., 2020). Genetic deletion of TLR4 has been shown to confer neuroprotection in ischemic brain injury (Hyakkoku et al., 2010), while its activation induces NF-κB phosphorylation, elevates inflammatory cytokines, and promotes neuroinflammation and neuronal apoptosis (Yao et al., 2023). And another study showed that TLR4 inhibition ameliorates cognitive impairment, restores hippocampal tissue damage, and alleviates neuroinflammation after hypoxic ischemic brain damage (Zou et al., 2023). In the brain, TLR4 is widely expressed on the surface of microglia and is involved in the recognition of LPS, which is mediated by the adapter MyD88 (Yin et al., 2020). This facilitates the entry of cytoplasmic NF-κB into the nucleus and promotes the activation of the NF-κB complex, which then involves the transcriptional expression of downstream inflammatory cytokines. TLR4-initiated signaling mediates the phosphorylation and degradation of IKB-α, which allows the NF-κB p65 subunit to shuttle into the nucleus, where it binds to specific DNA shared sequences, thereby enhancing the transcription of inflammation-associated proteins((e.g., IL-6, TNF-α, IL-1β) (Jin et al., 2019).

Neuroinflammation has been implicated as a key contributor to post-stroke dementia (Yang et al., 2022). Consistent with this, network pharmacology predictions in the present study suggested that Puerarin may inhibit inflammatory responses through the Toll-like receptor and NF-κB signaling pathways. Evidence that TLR4/MyD88/NF-κB signaling pathway is involved in the pathogenesis of many cerebrovascular diseases. The inhibition of the over-activation of TLR4/MyD88/NF-κB signaling pathway can reduced neuronal death and neuroinflammation in middle cerebral artery occlusion/reperfusion (MCAO/R) models (Zhang et al., 2024). Furthermore, inhibiting the TLR4/MyD88/NF-κB signaling pathway can mitigate neuronal death, protect the BBB, and restore cognitive dysfunction in vascular dementia mice (Zou et al., 2023). Therefore, inhibiting the TLR4/MyD88/NF-κB pathway may be an effective strategy for treating VaD. However, the role of Puerarin in regulating this pathway has been seldom investigated in the context of VaD. The present study addresses this gap by demonstrating that Puerarin treatment suppresses the activation of TLR4, MyD88, and phosphorylated NF-κB p65. To further verify the pathway’s involvement, pharmacological intervention with the TLR4 agonist LPS was employed. LPS administration reversed the beneficial effects of Puerarin, exacerbating neuroinflammation and memory impairment, thereby underscoring the pivotal role of TLR4 in VaD pathogenesis. Collectively, these findings indicate that the anti-inflammatory effects of Puerarin in VaD are mediated, at least in part, through regulation of the TLR4/MyD88/NF-κB pathway, positioning Puerarin as a potential TLR4 inhibitor for neuroinflammatory conditions.

While the TLR4 agonist LPS was employed to validate the involvement of this pathway, it is important to acknowledge the limitations of this pharmacological approach. LPS is a potent inflammatory stimulus that may potentially activate pattern recognition receptors or signaling cascades beyond TLR4. Therefore, while our findings strongly suggest that the anti-inflammatory effects of Puerarin are mediated via the TLR4/MyD88/NF-κB pathway, the possibility of contributions from other signaling pathways cannot be entirely excluded. Future studies utilizing more specific genetic approaches, such as TLR4 knockout or siRNA-mediated knockdown models, would provide more definitive evidence and are warranted to further clarify the precise molecular mechanism.

In conclusion, the present study demonstrates that Puerarin treatment alleviates cognitive impairment and neuroinflammation in VaD rats by inhibiting the TLR4/MyD88/NF-κB inflammatory signaling pathway. These findings not only elucidate a key inflammatory mechanism in VaD but also position Puerarin as a promising therapeutic candidate for its treatment.

## Data Availability

The original contributions presented in the study are included in the article/Supplementary Material, further inquiries can be directed to the corresponding authors.
