# Supplementary figures and images for: Investigating the therapeutic mechanism of Puerarin in vascular dementia: an integrated approach combining network pharmacology and experimental validation
Source: Front Pharmacol. 2026 Apr 29;17:1796295. doi: 10.3389/fphar.2026.1796295 (PMC13168132; doi:10.3389/fphar.2026.1796295)

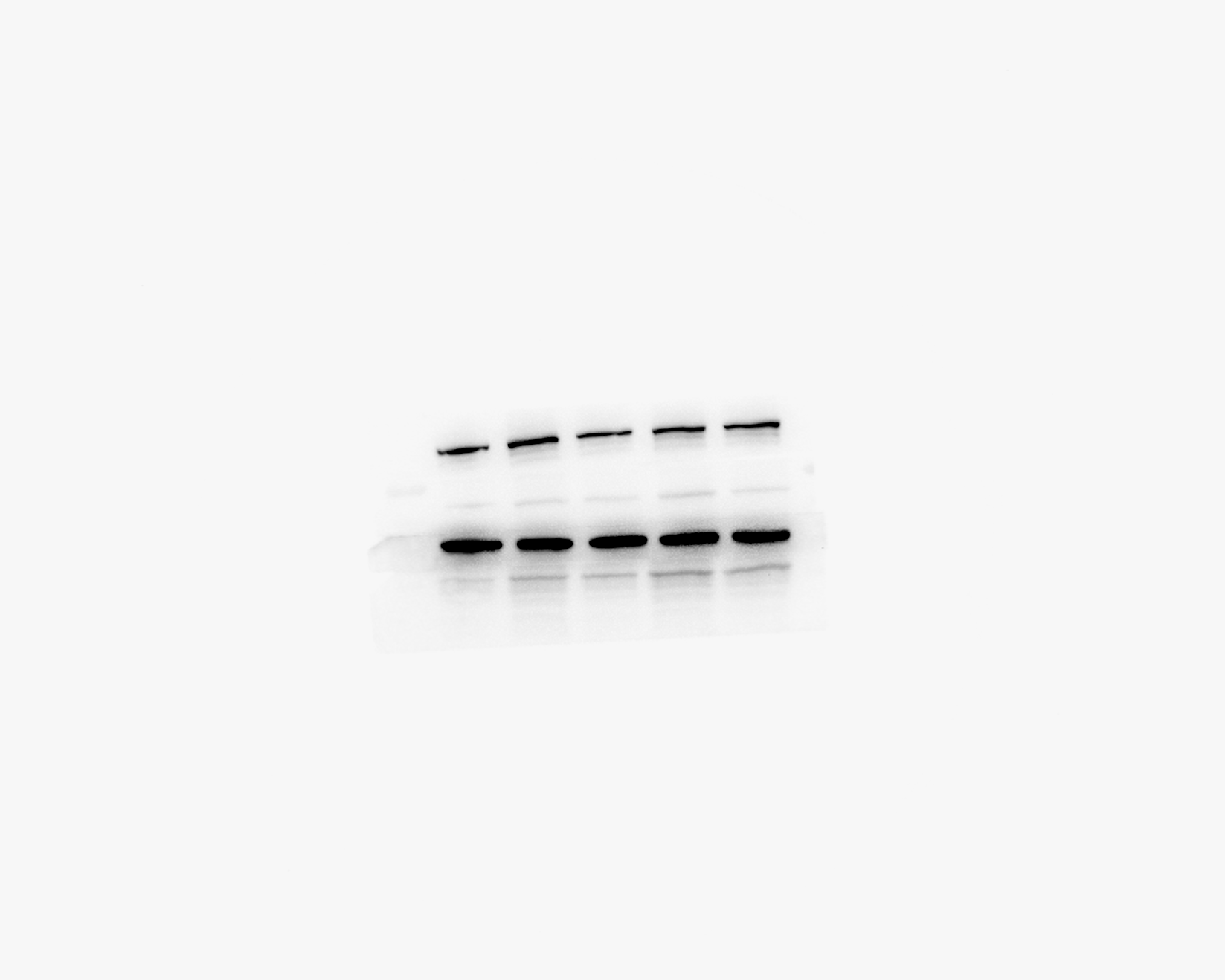

Supplement: Supplementary file 1 [file DataSheet3.ZIP › Myd88/H (1).tif]

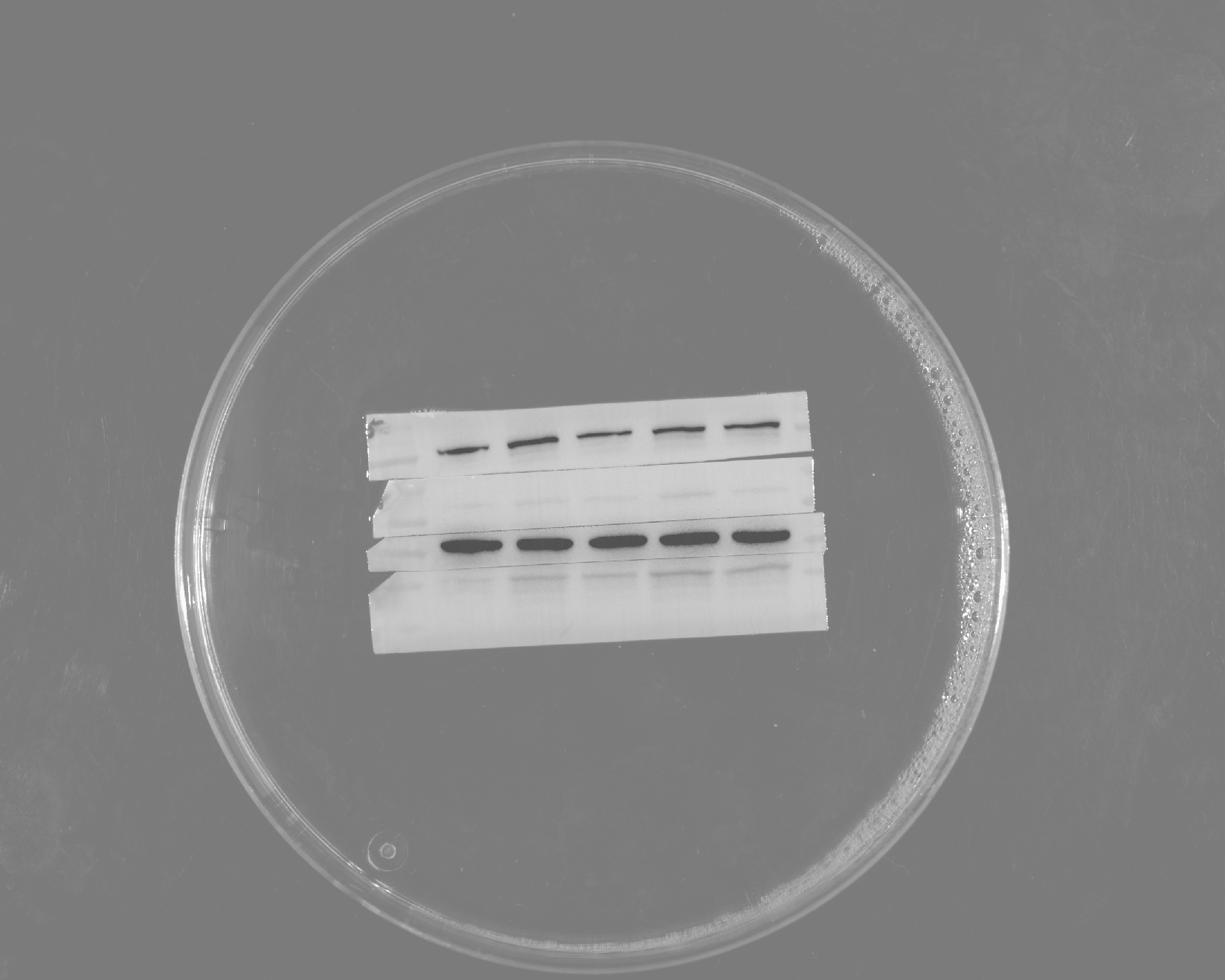

Supplement: Supplementary file 1 [file DataSheet3.ZIP › Myd88/H (2).tif]

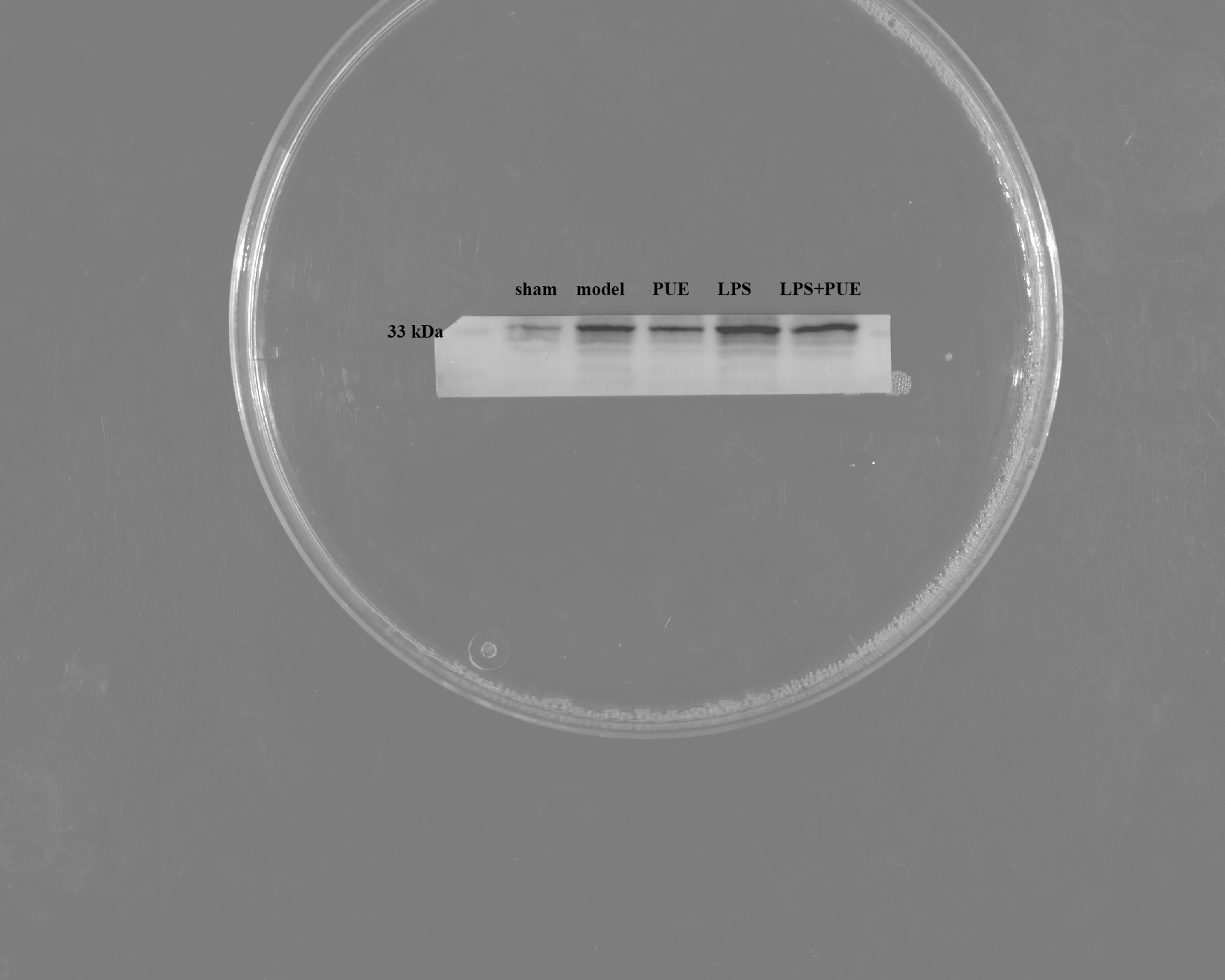

Supplement: Supplementary file 1 [file DataSheet3.ZIP › Myd88/MyD88 (1).tif]

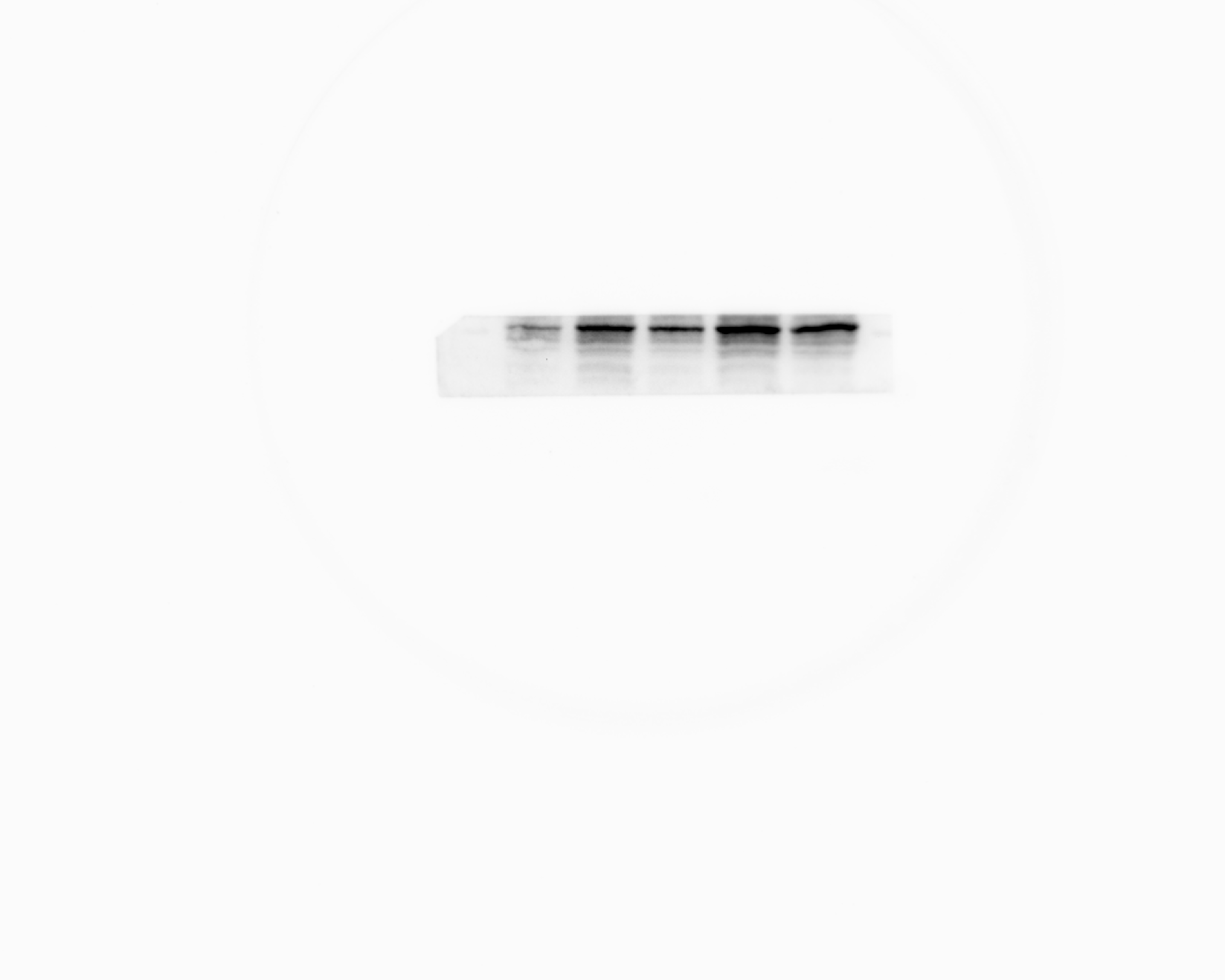

Supplement: Supplementary file 1 [file DataSheet3.ZIP › Myd88/MyD88 (2).tif]

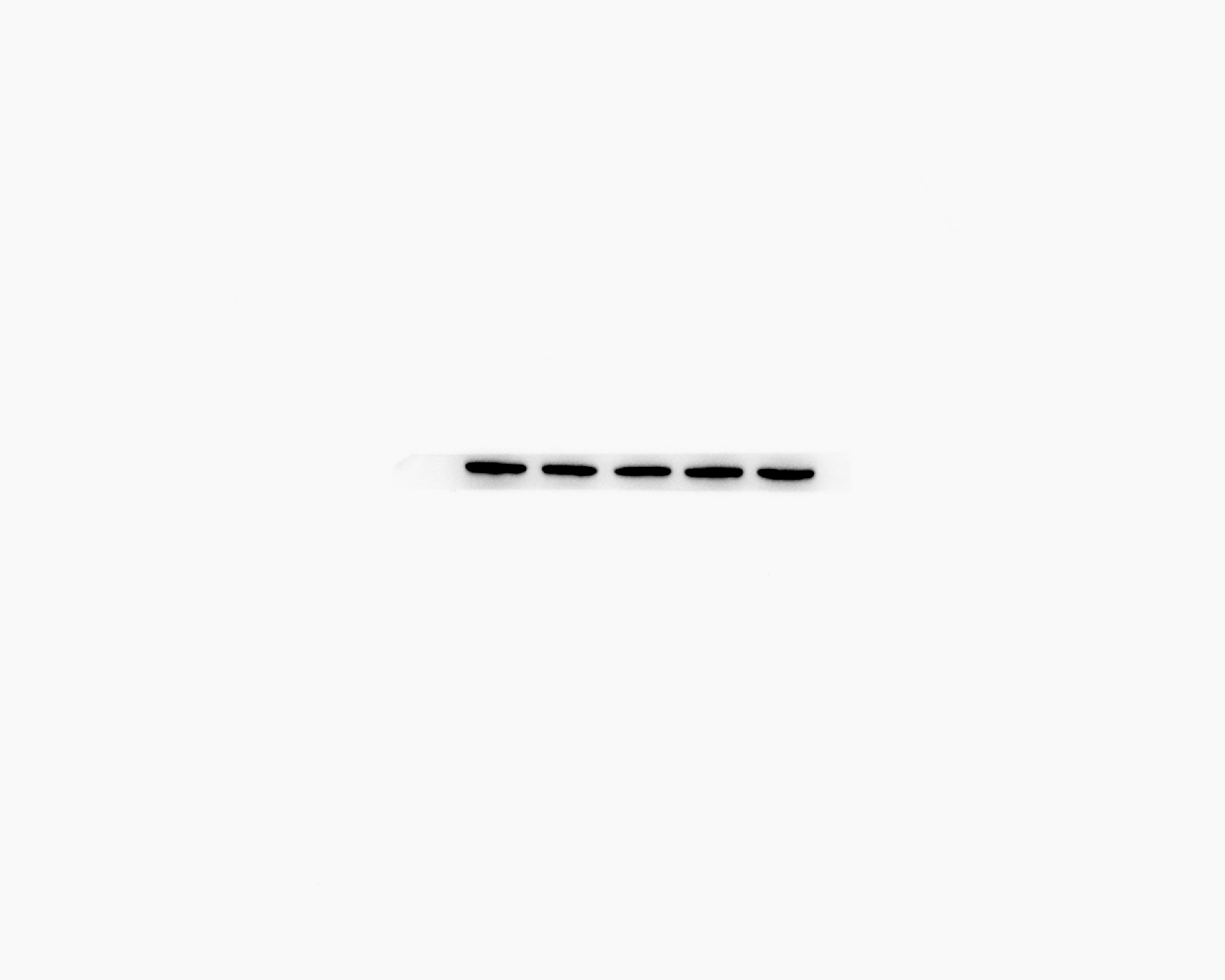

Supplement: Supplementary file 1 [file DataSheet3.ZIP › Myd88/β-actin (1).tif]

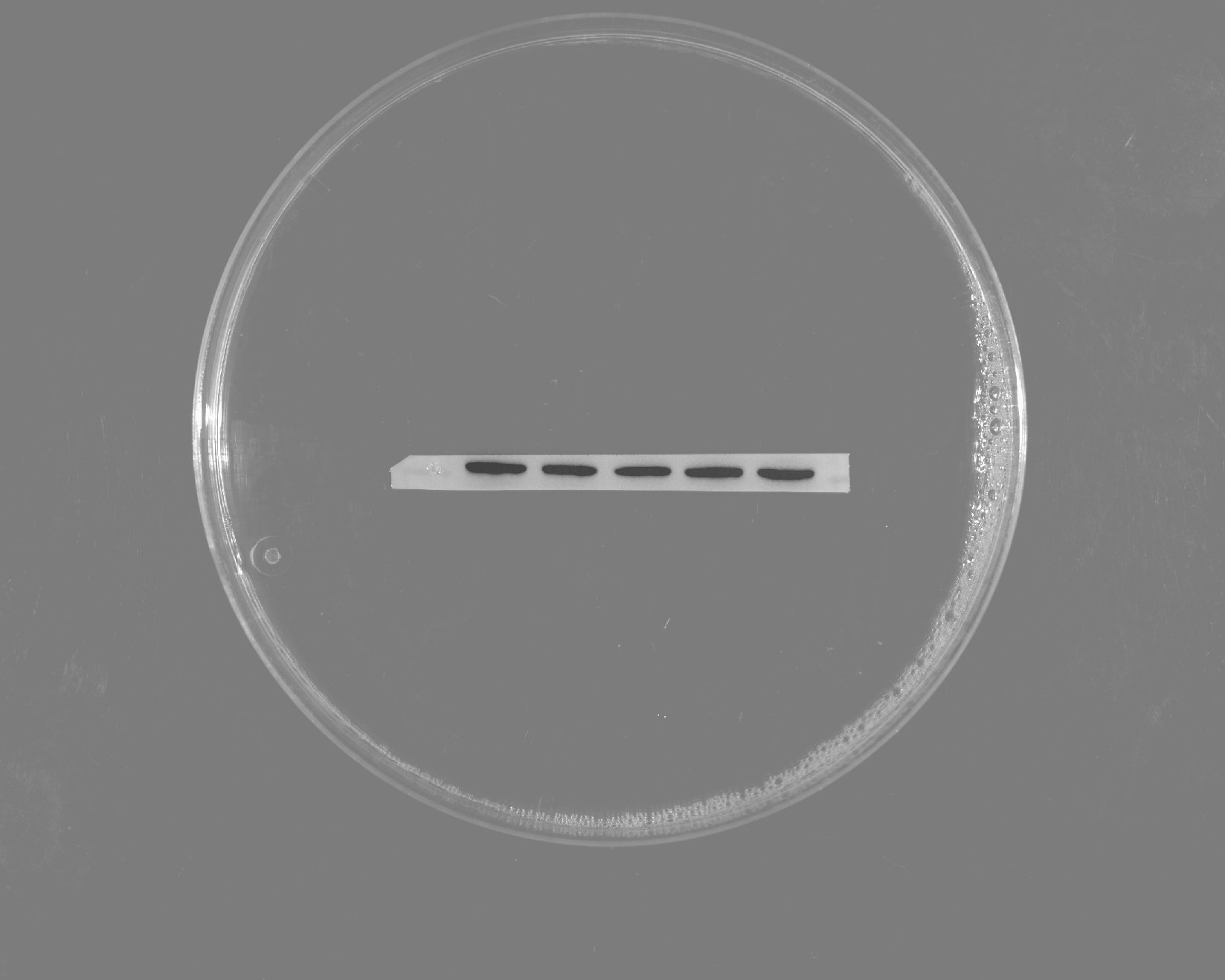

Supplement: Supplementary file 1 [file DataSheet3.ZIP › Myd88/β-actin (2).tif]

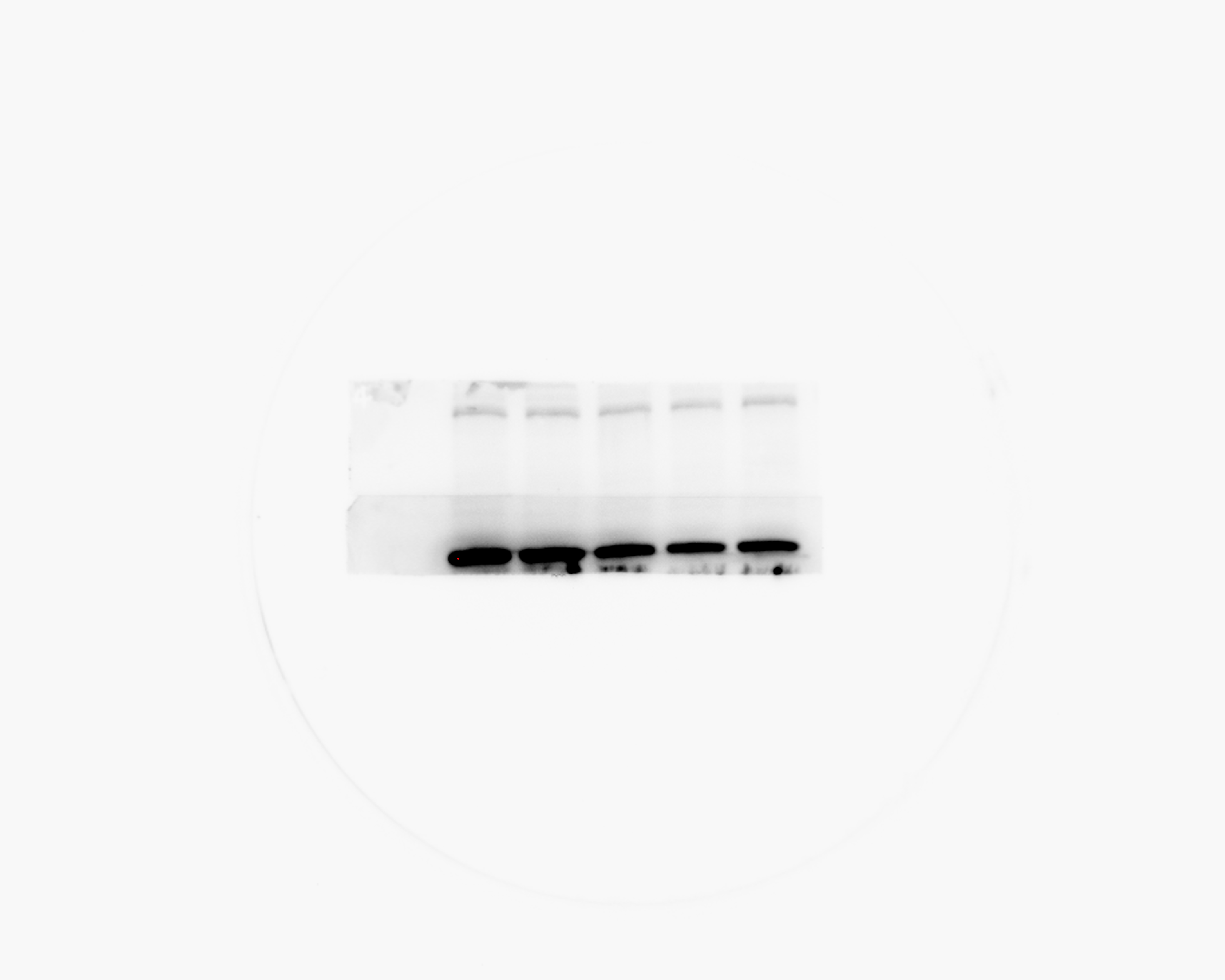

Supplement: Supplementary file 1 [file DataSheet3.ZIP › P65/H (1).tif]

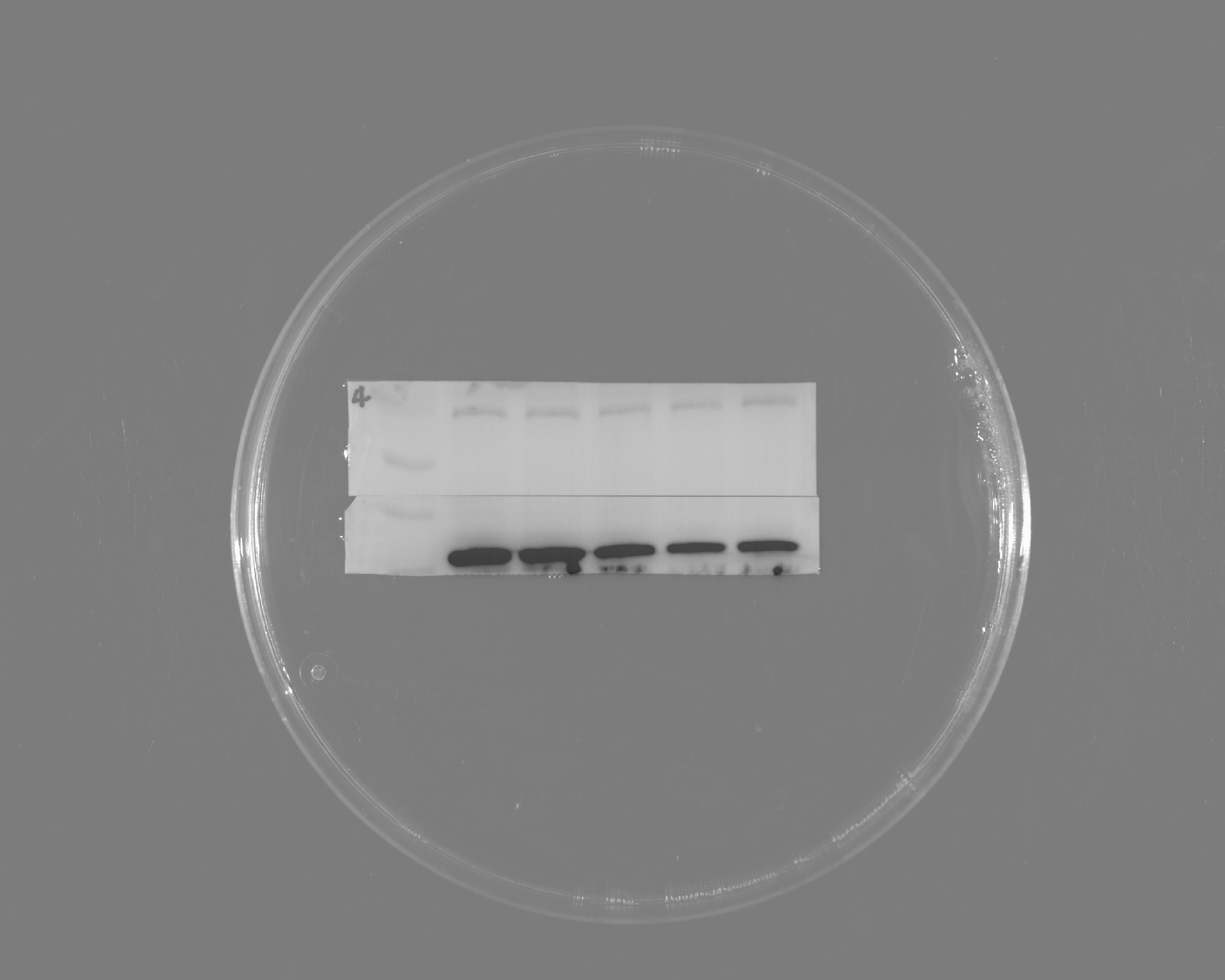

Supplement: Supplementary file 1 [file DataSheet3.ZIP › P65/H (2).tif]

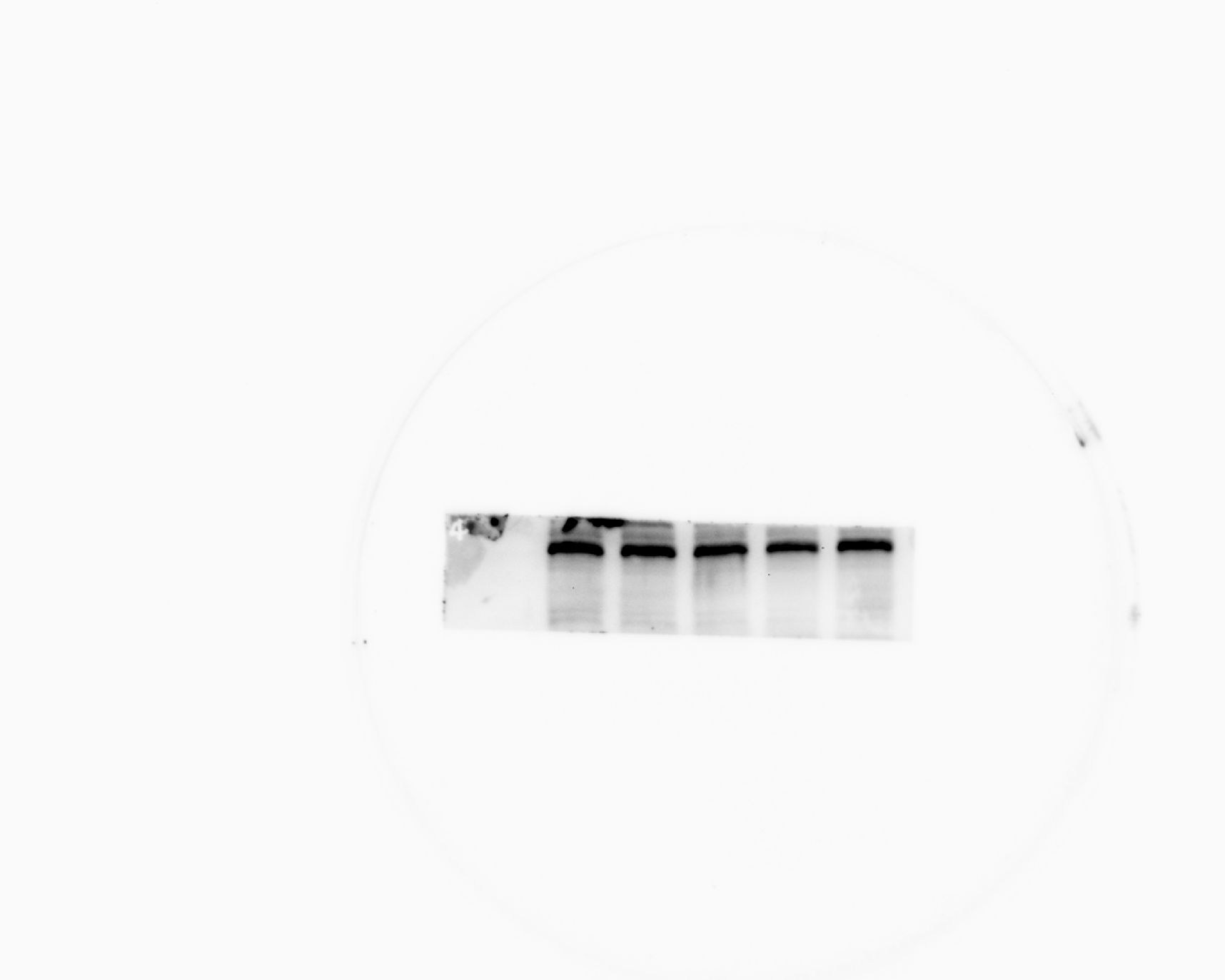

Supplement: Supplementary file 1 [file DataSheet3.ZIP › P65/P65 (1).tif]

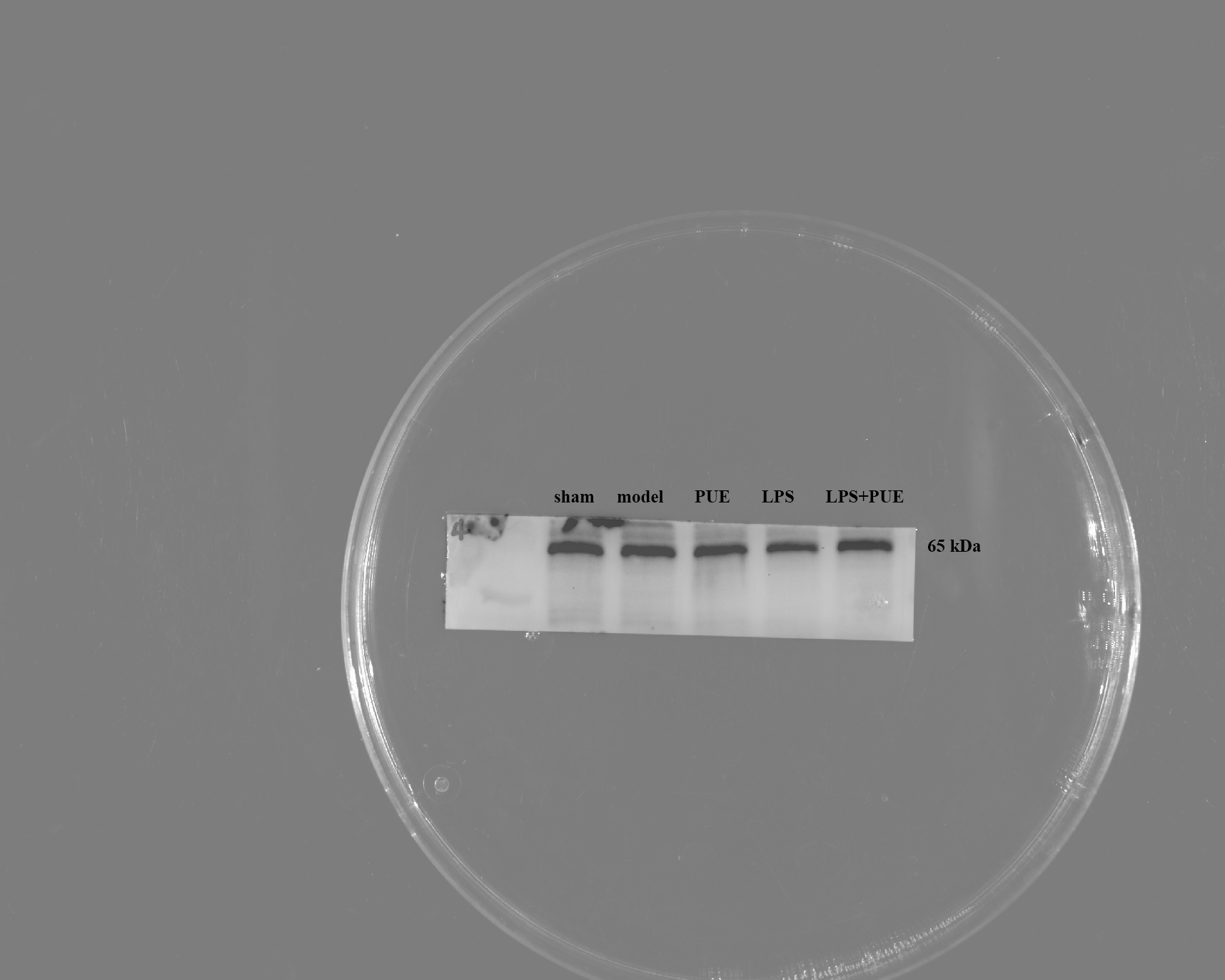

Supplement: Supplementary file 1 [file DataSheet3.ZIP › P65/P65 (2).tif]

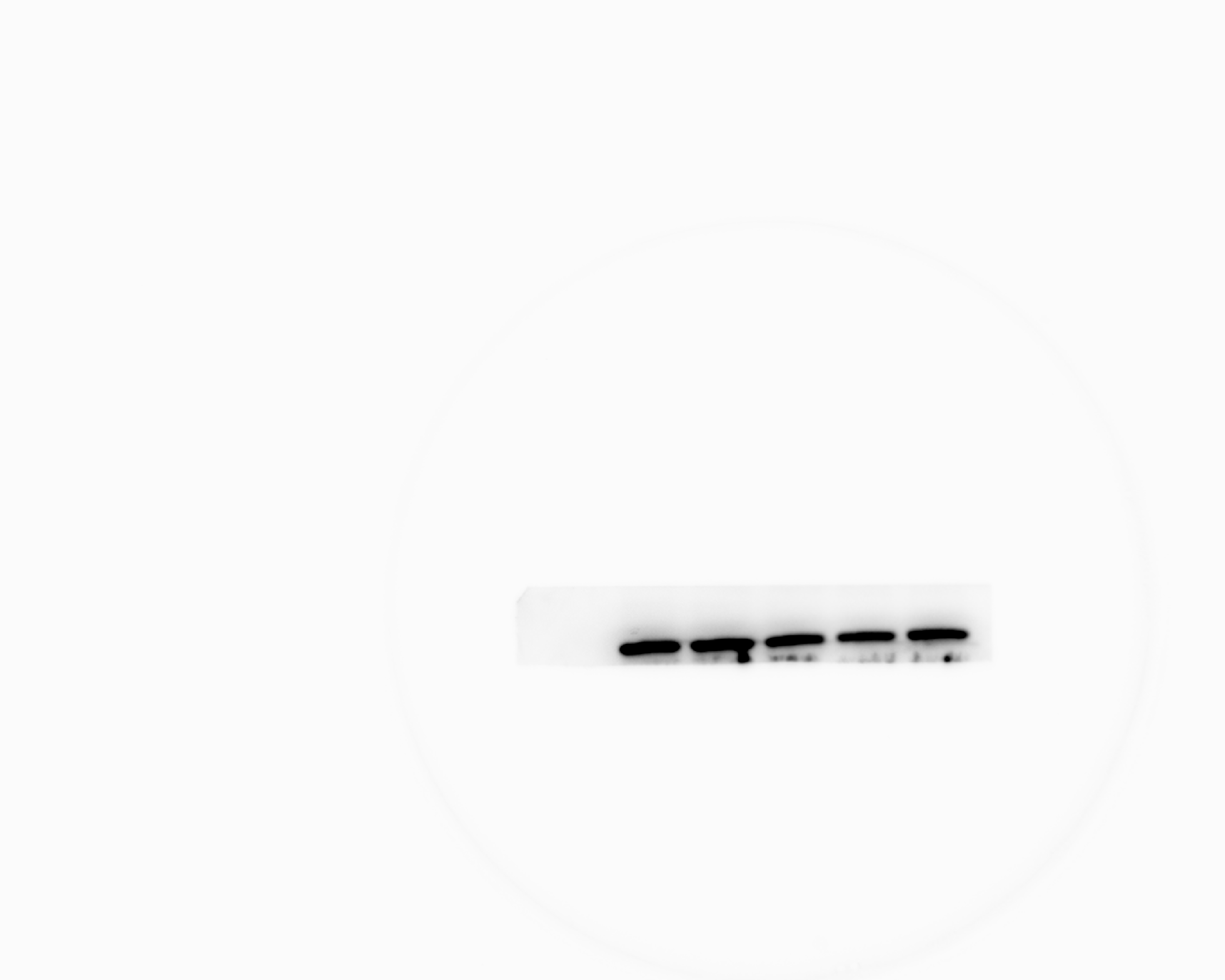

Supplement: Supplementary file 1 [file DataSheet3.ZIP › P65/β-actin (1).tif]

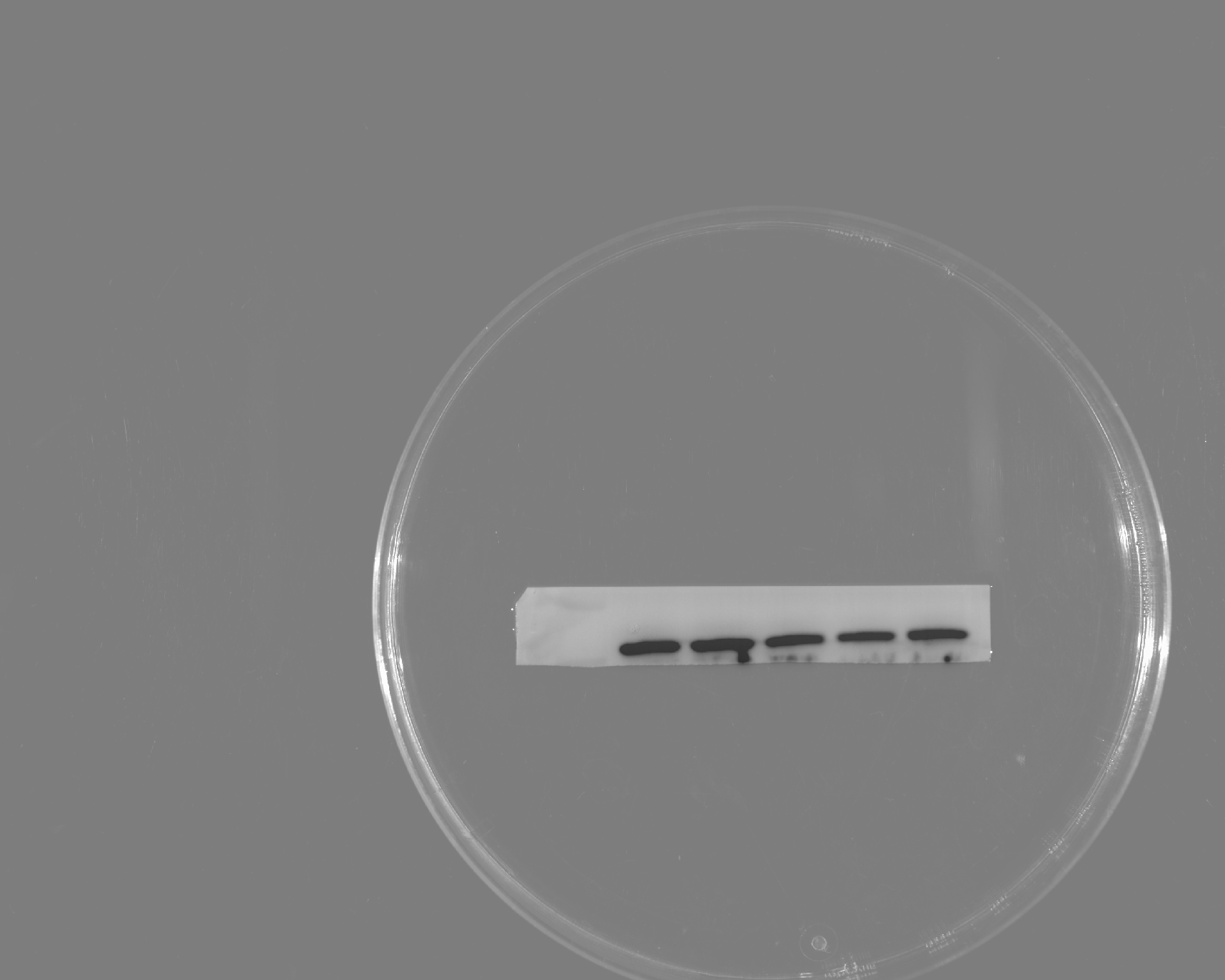

Supplement: Supplementary file 1 [file DataSheet3.ZIP › P65/β-actin (2).tif]

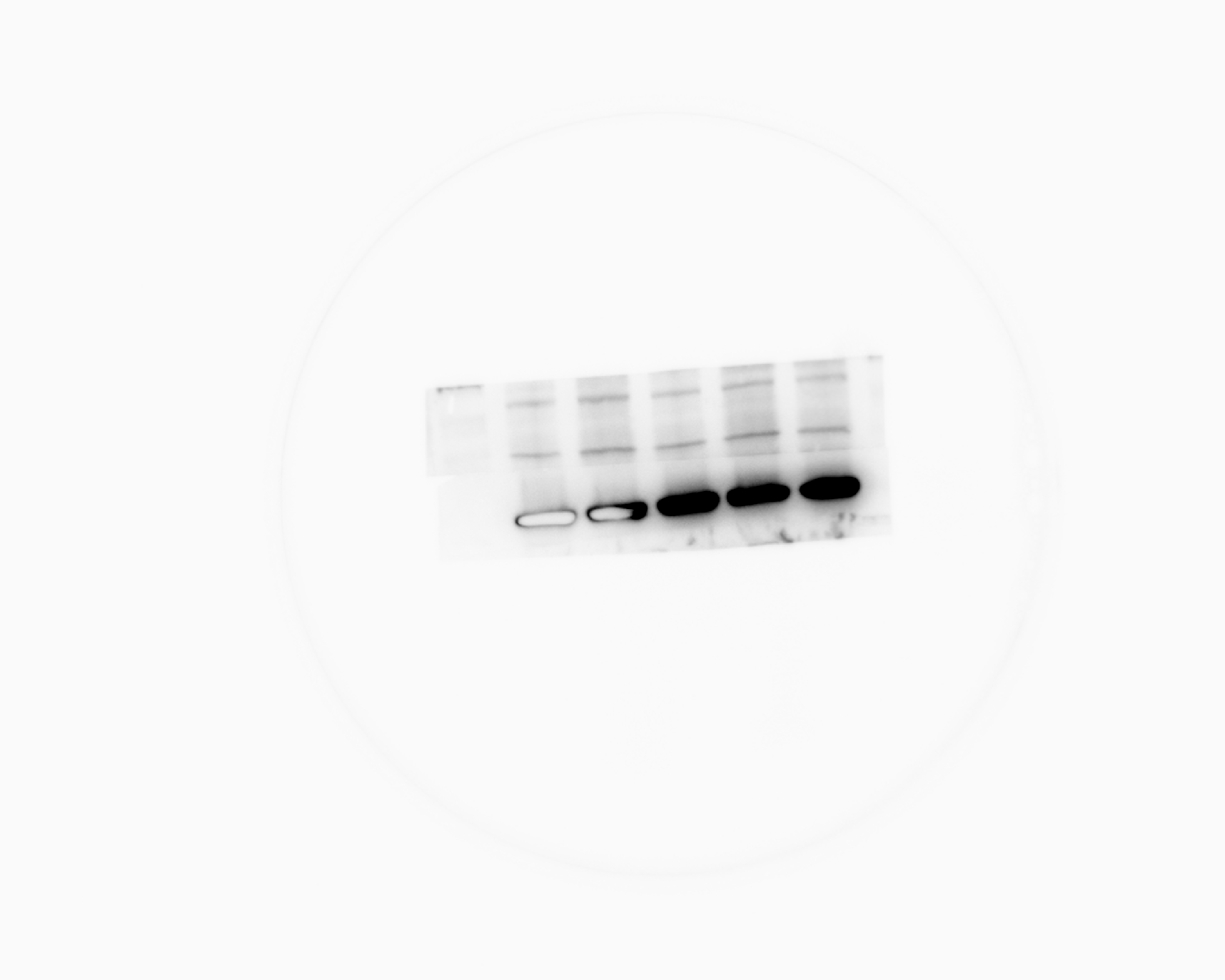

Supplement: Supplementary file 1 [file DataSheet3.ZIP › PP65/H (1).tif]

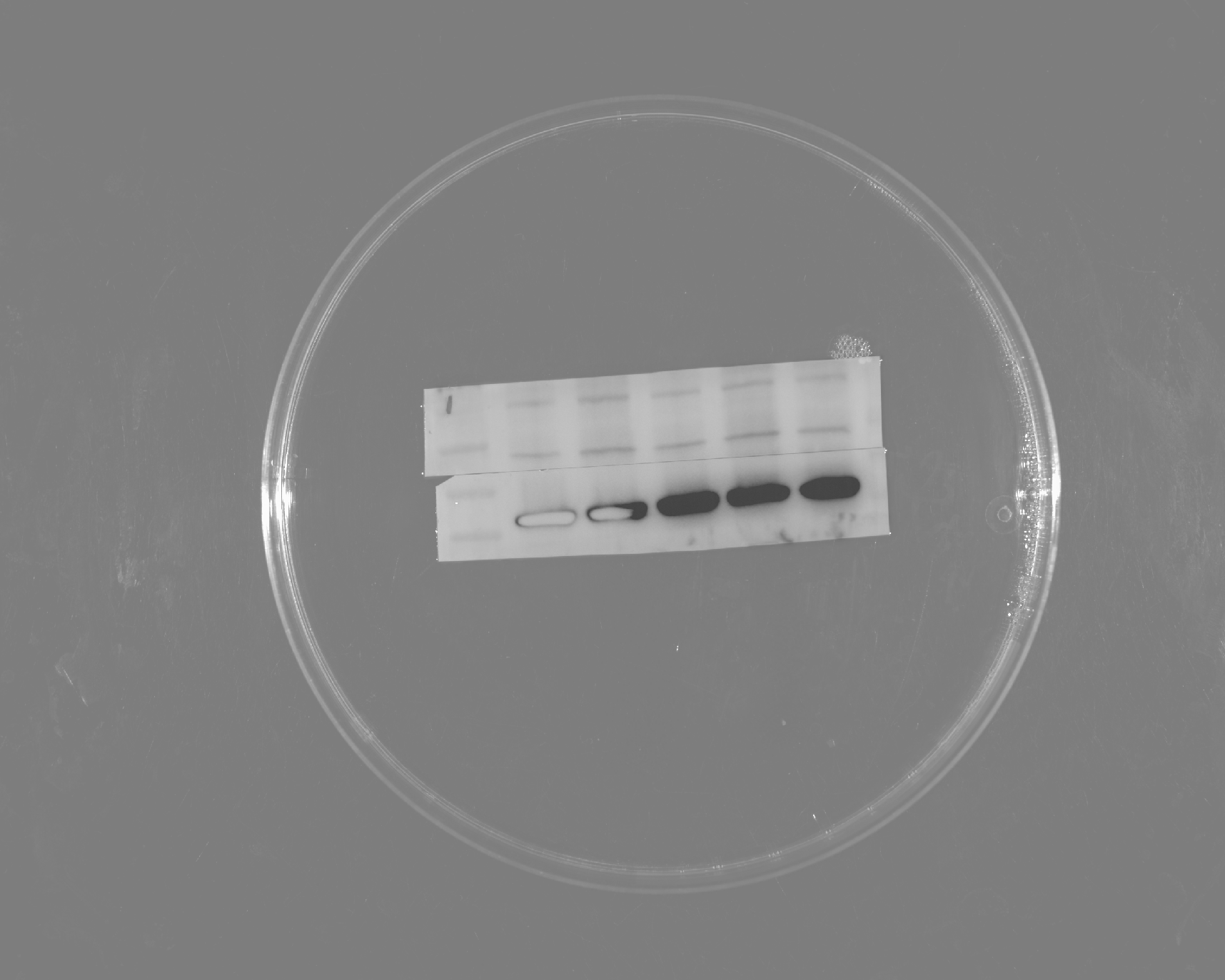

Supplement: Supplementary file 1 [file DataSheet3.ZIP › PP65/H (2).tif]

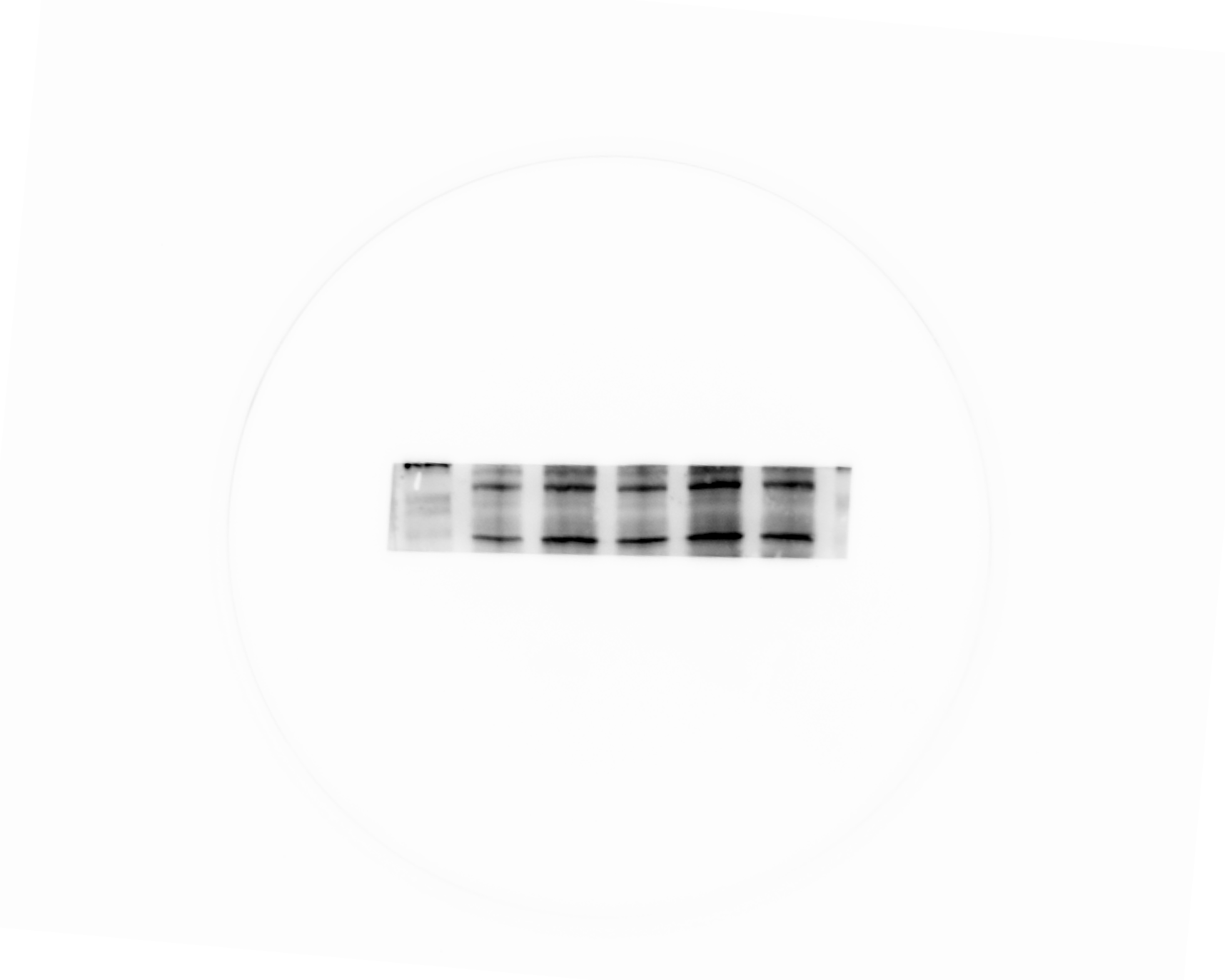

Supplement: Supplementary file 1 [file DataSheet3.ZIP › PP65/PP65 (1).tif]

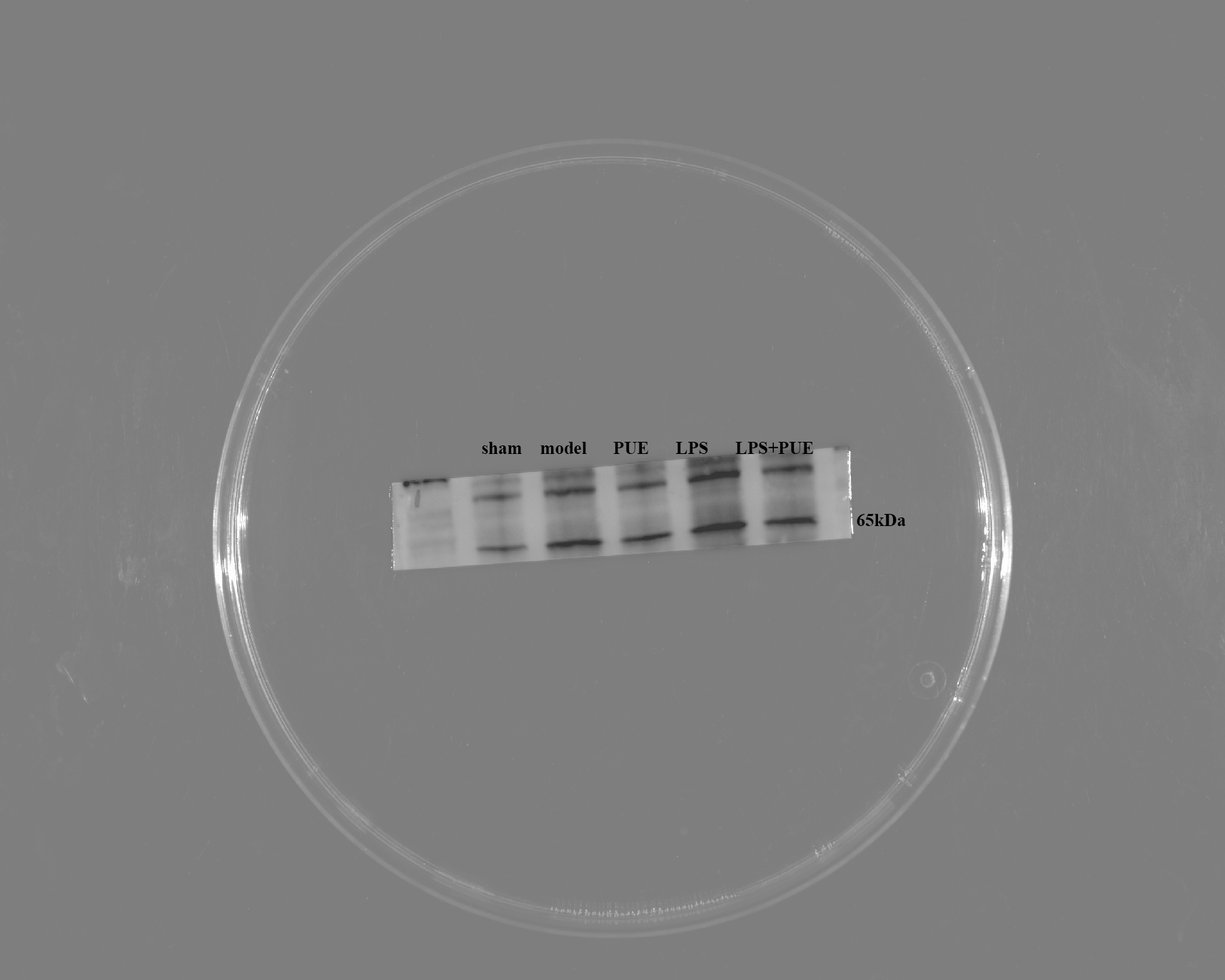

Supplement: Supplementary file 1 [file DataSheet3.ZIP › PP65/PP65 (2).tif]

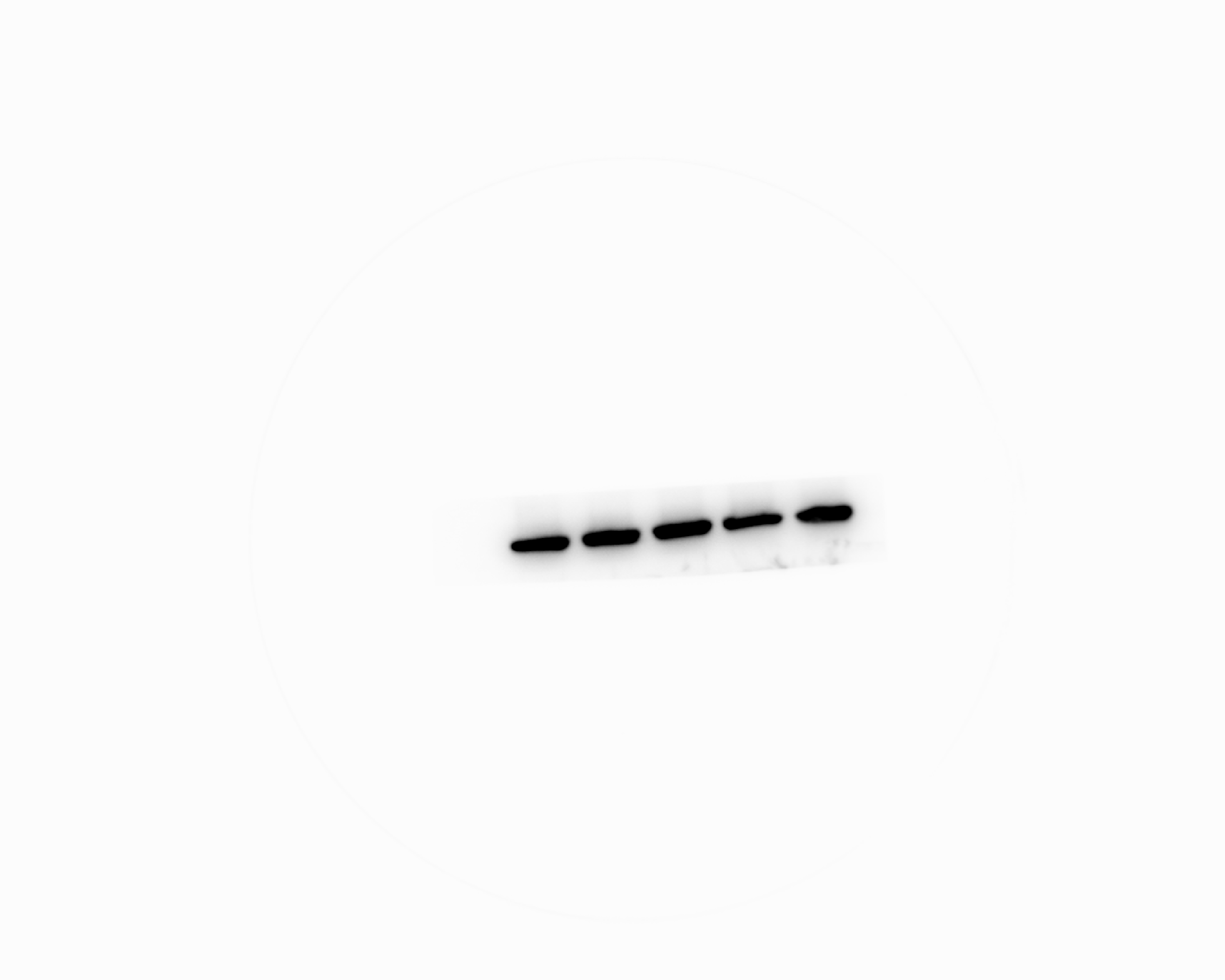

Supplement: Supplementary file 1 [file DataSheet3.ZIP › PP65/β-actin (1).tif]

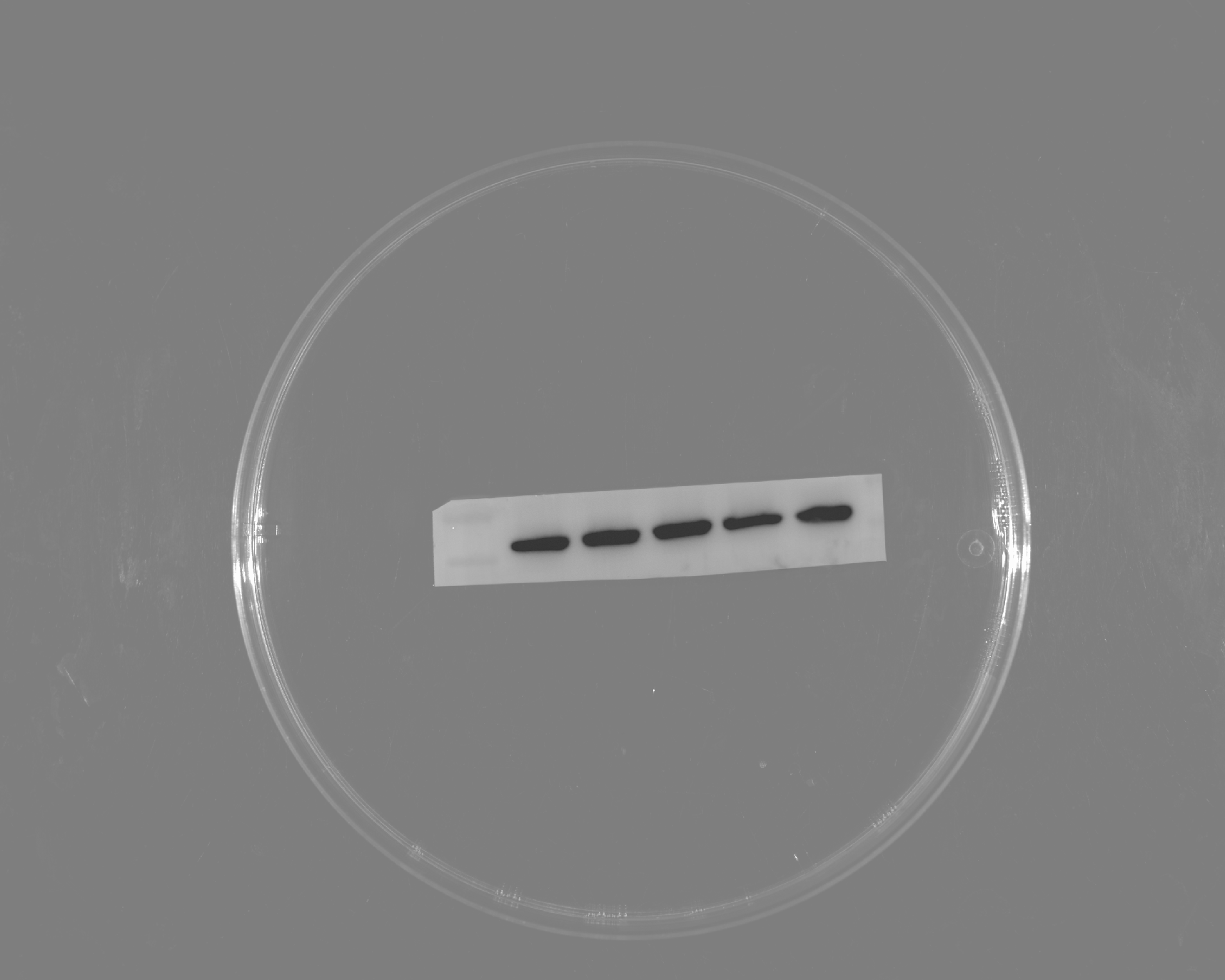

Supplement: Supplementary file 1 [file DataSheet3.ZIP › PP65/β-actin (2).tif]

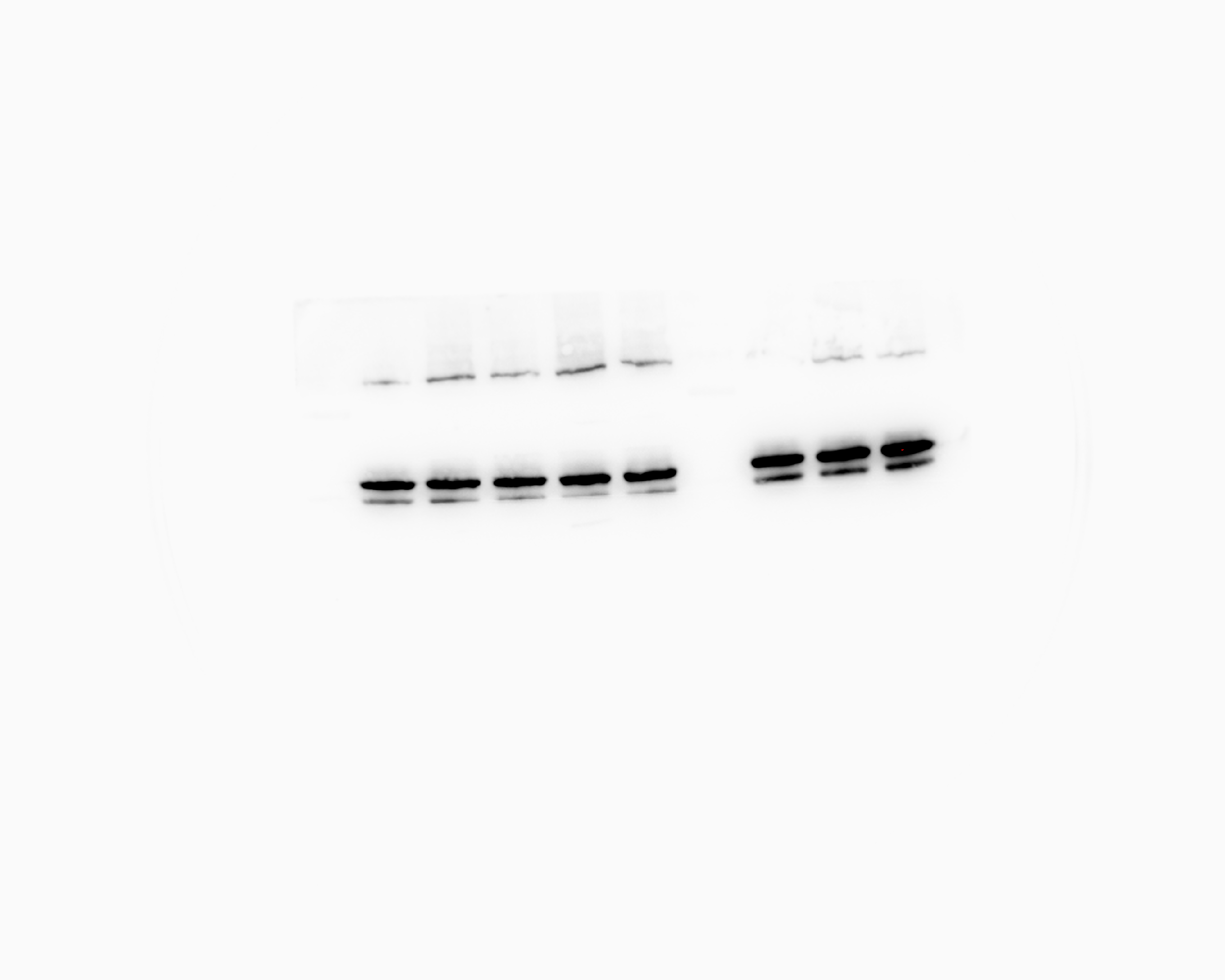

Supplement: Supplementary file 1 [file DataSheet3.ZIP › TLR4/H (1).tif]

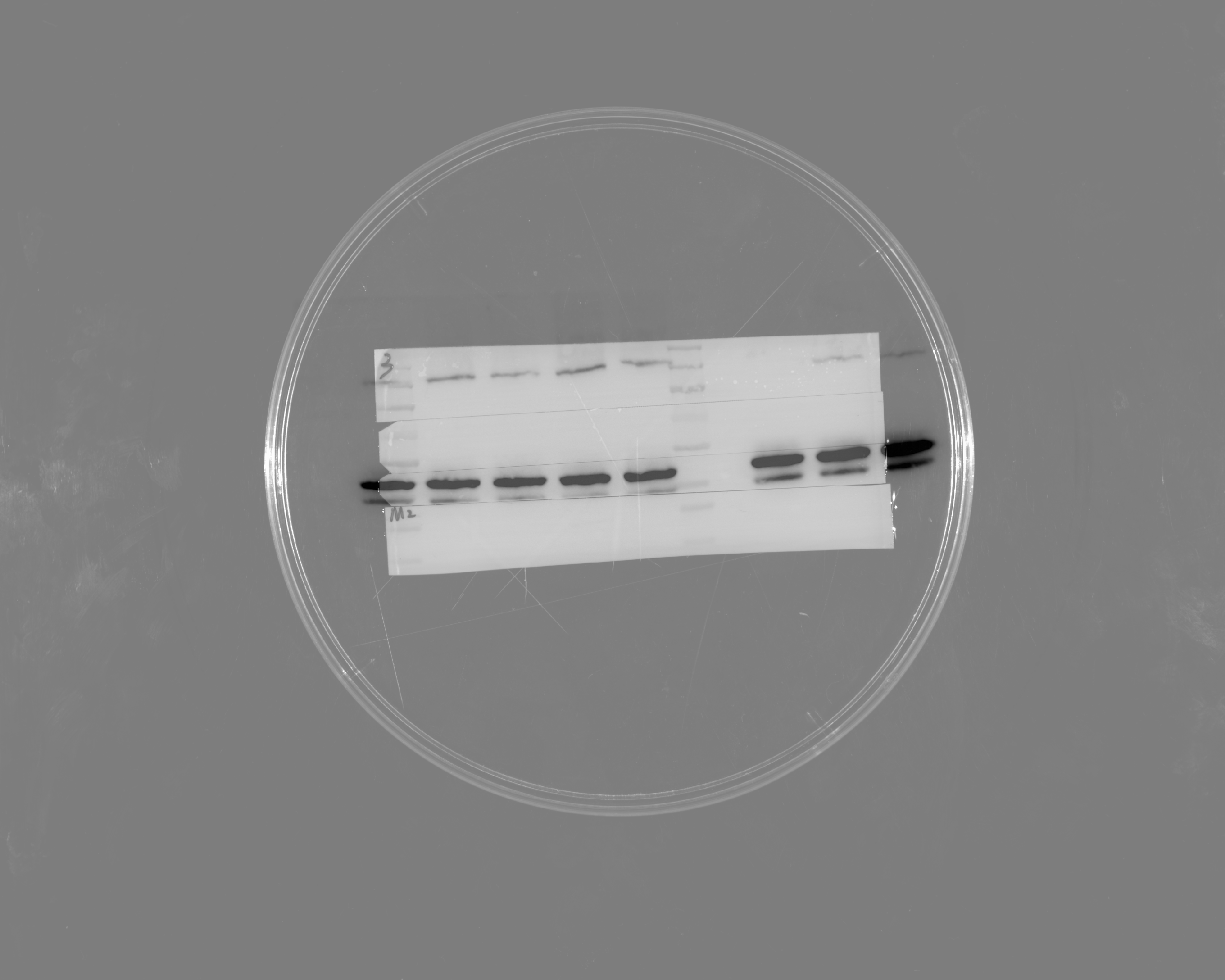

Supplement: Supplementary file 1 [file DataSheet3.ZIP › TLR4/H (2).tif]

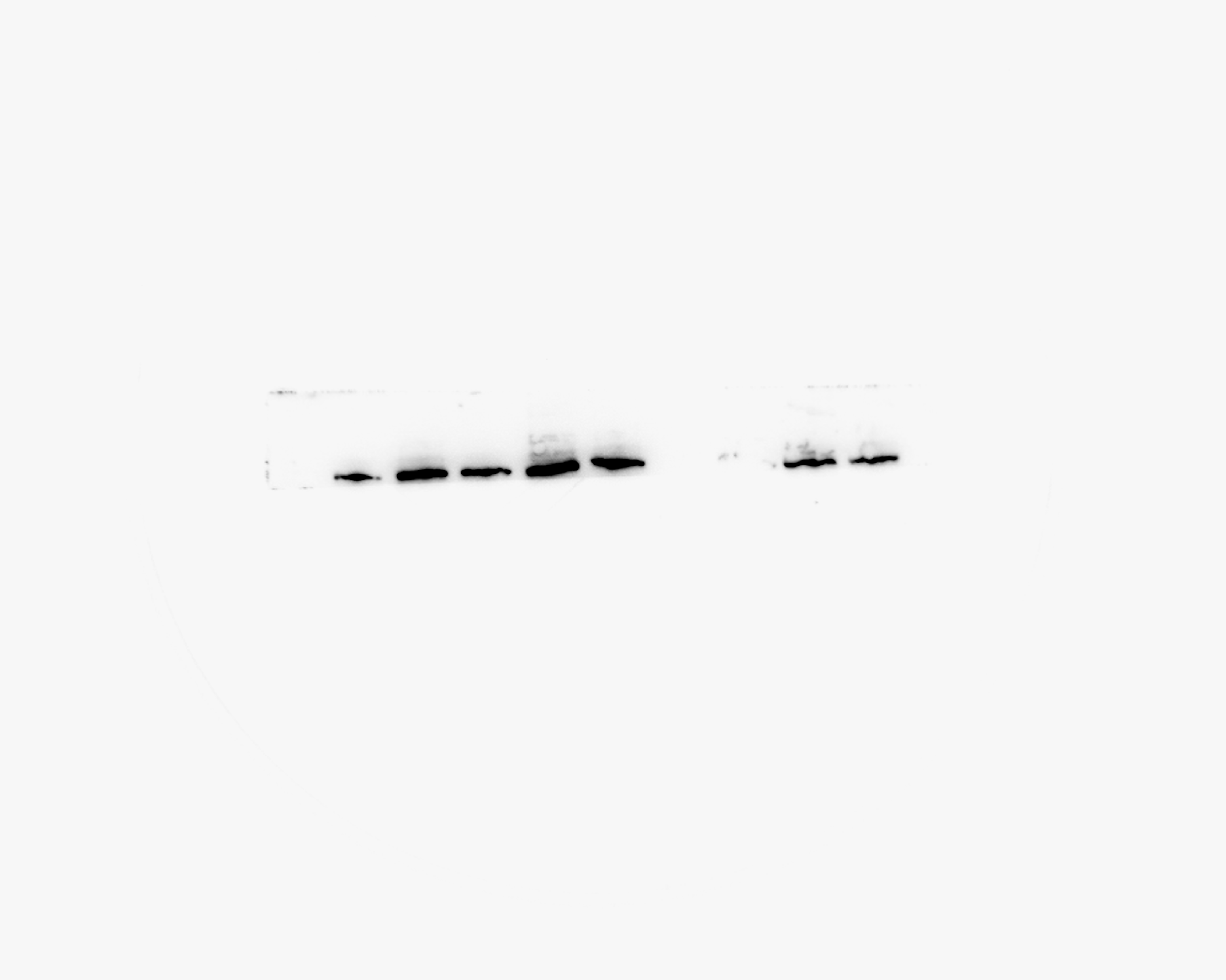

Supplement: Supplementary file 1 [file DataSheet3.ZIP › TLR4/TLR4 (1).tif]

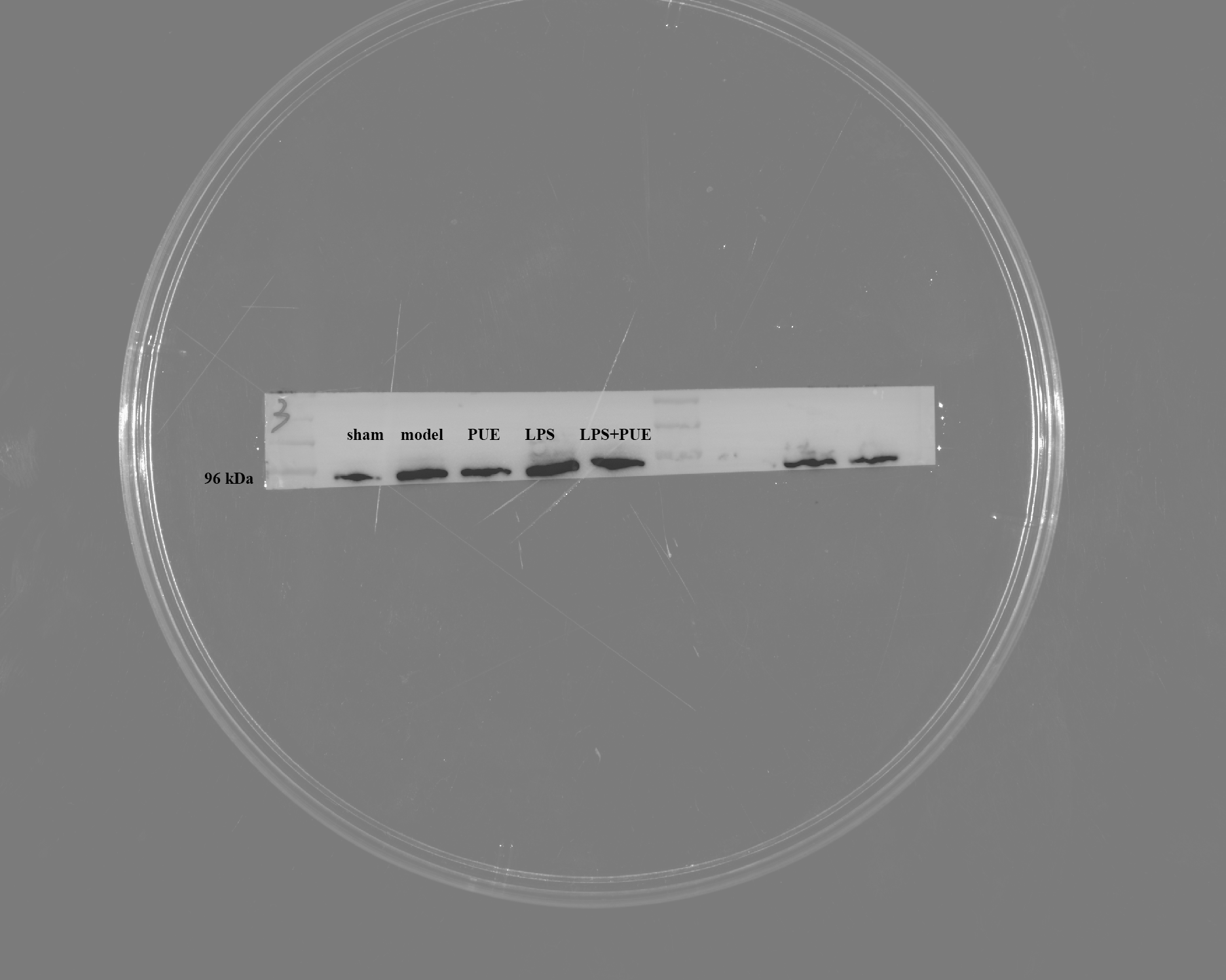

Supplement: Supplementary file 1 [file DataSheet3.ZIP › TLR4/TLR4 (2).tif]

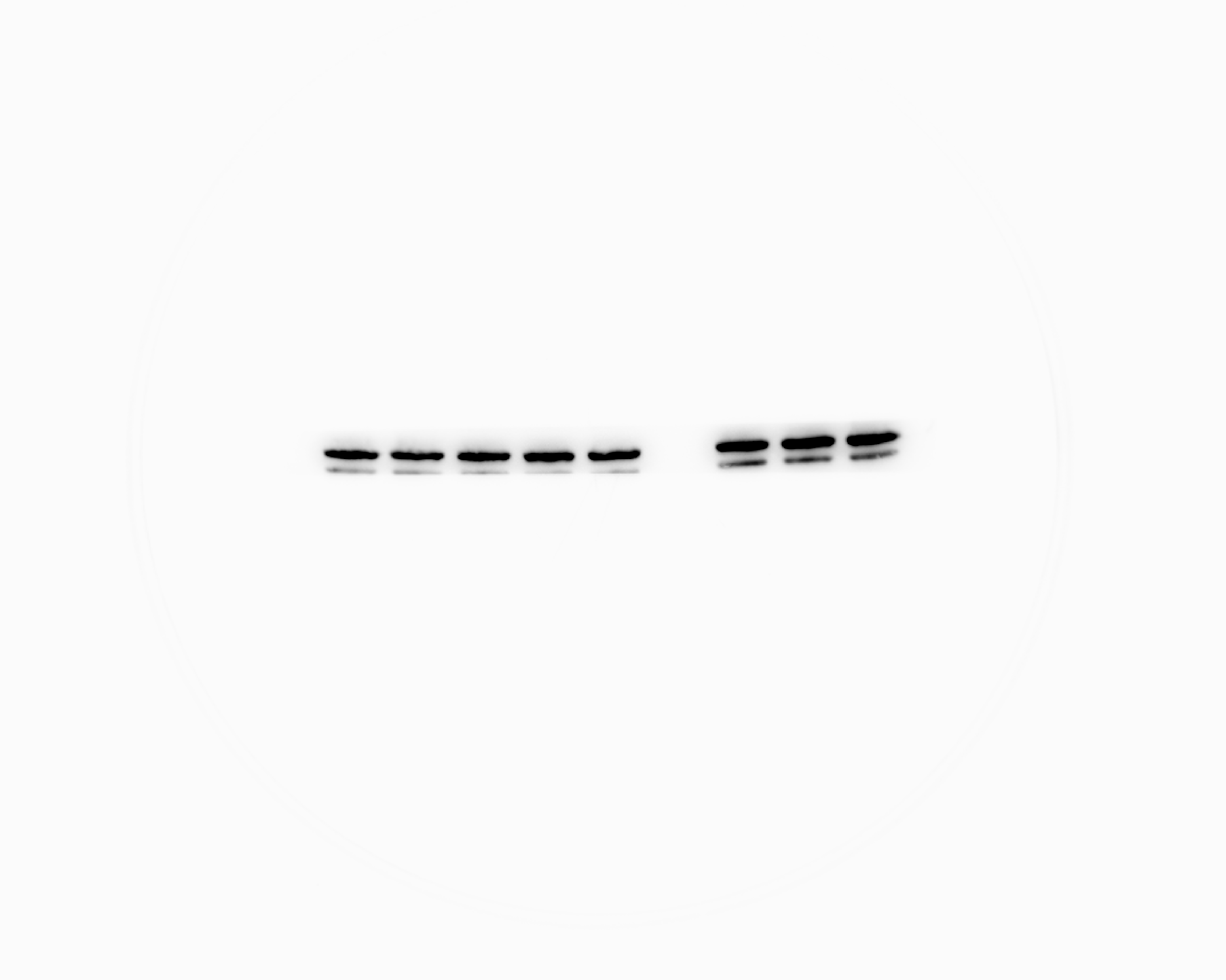

Supplement: Supplementary file 1 [file DataSheet3.ZIP › TLR4/β-actin (1).tif]

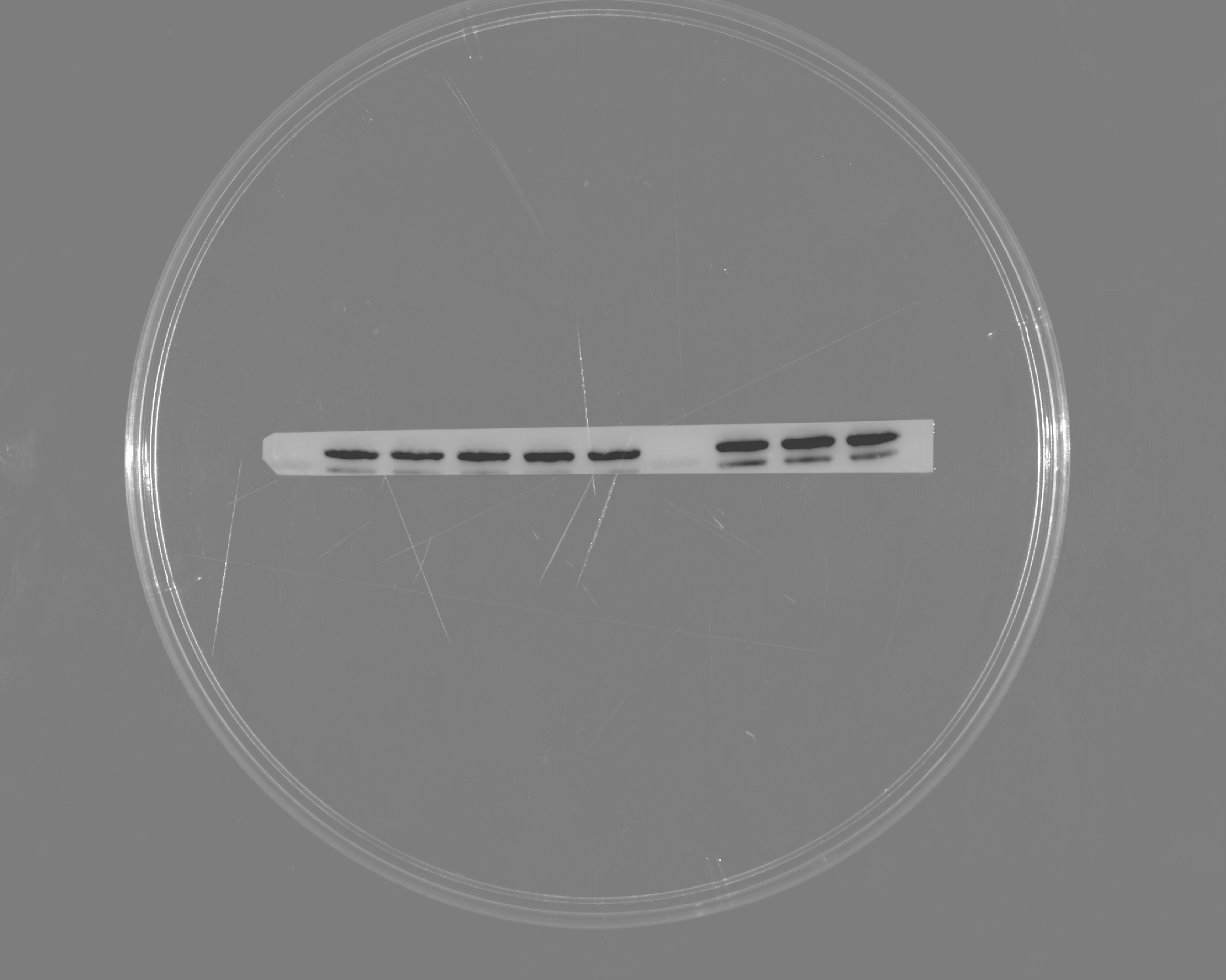

Supplement: Supplementary file 1 [file DataSheet3.ZIP › TLR4/β-actin (2).tif]

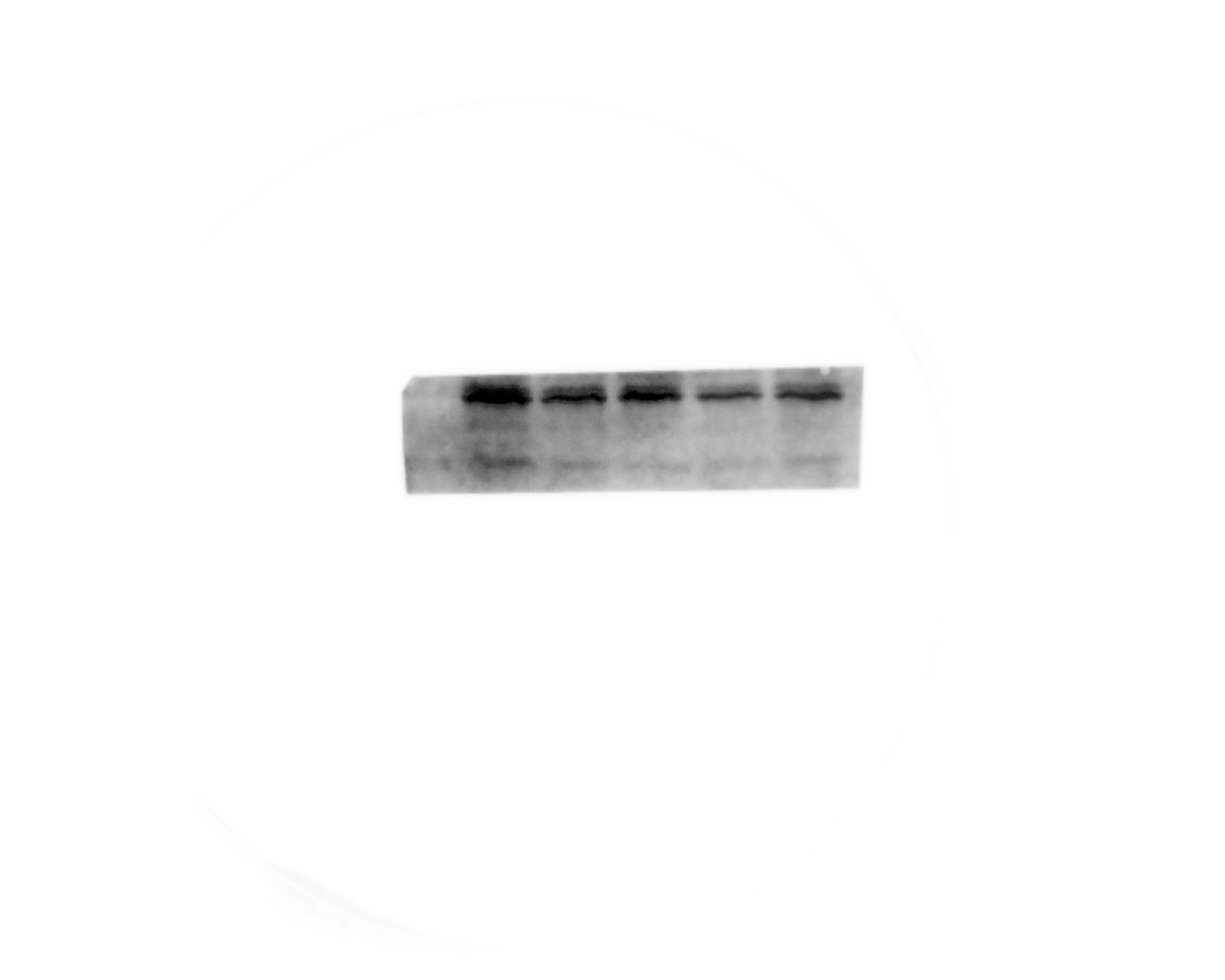

Supplement: Supplementary file 3 [file DataSheet1.ZIP › cladin5/claudin5 (1).tif]

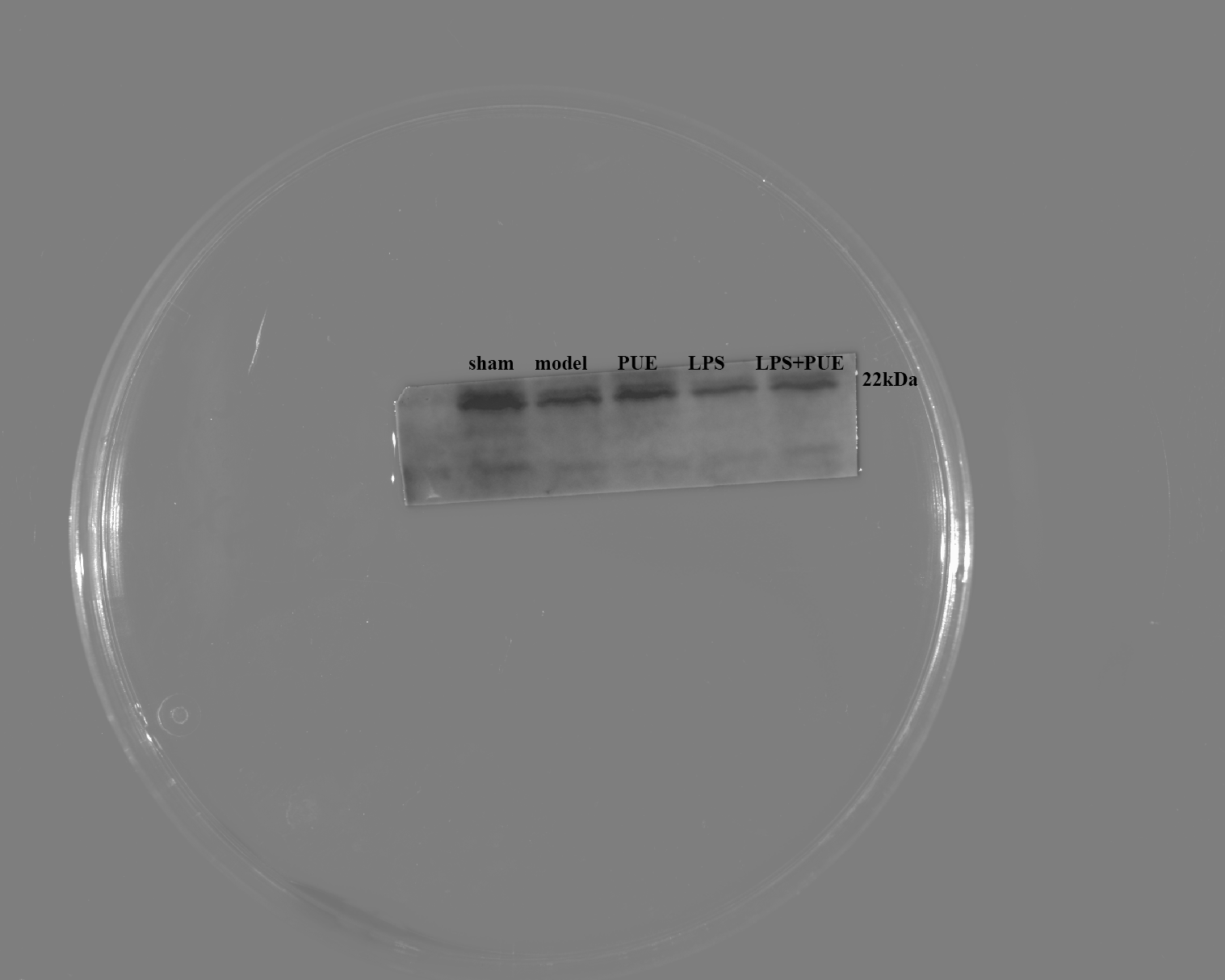

Supplement: Supplementary file 3 [file DataSheet1.ZIP › cladin5/claudin5 (2).tif]

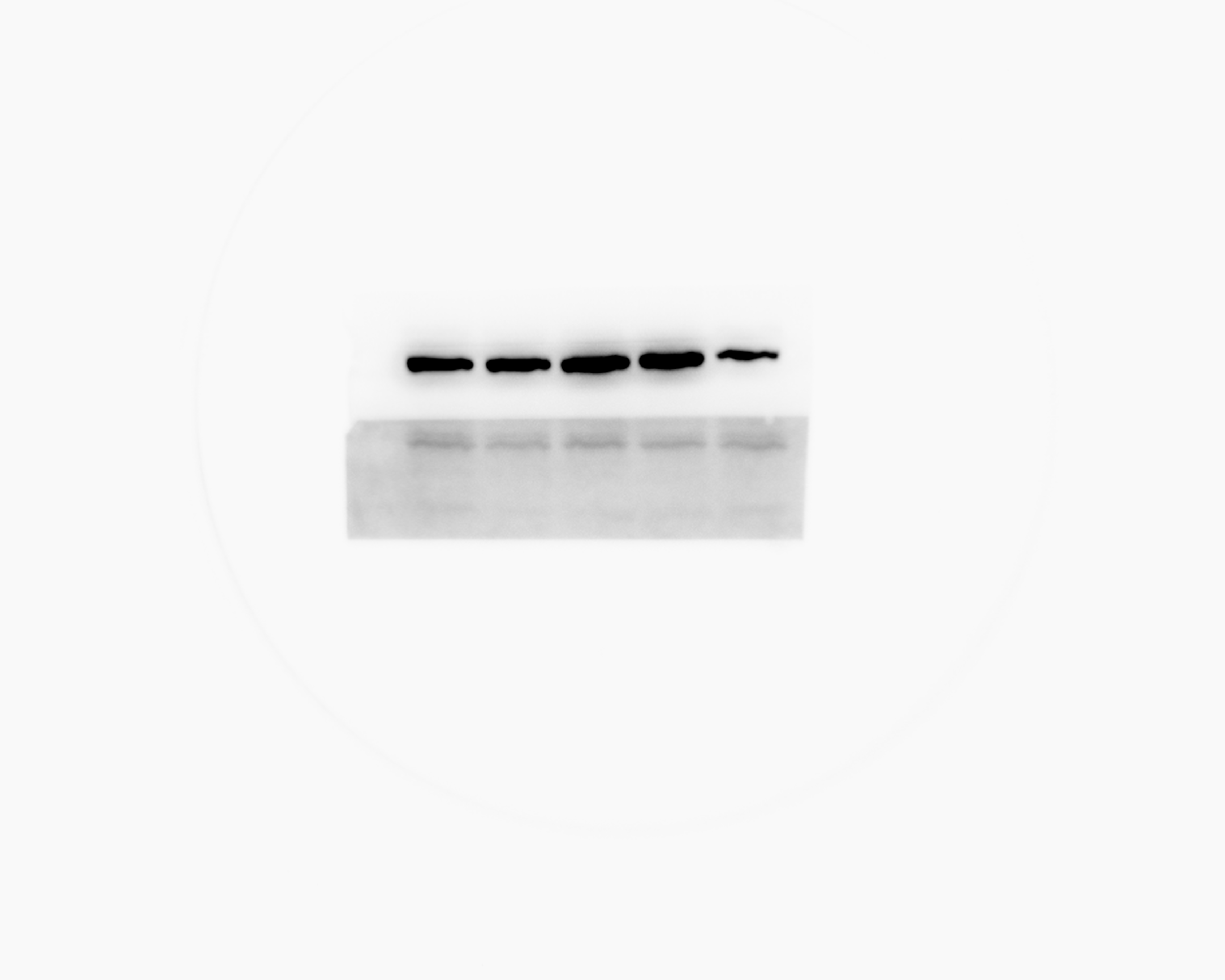

Supplement: Supplementary file 3 [file DataSheet1.ZIP › cladin5/H(1).tif]

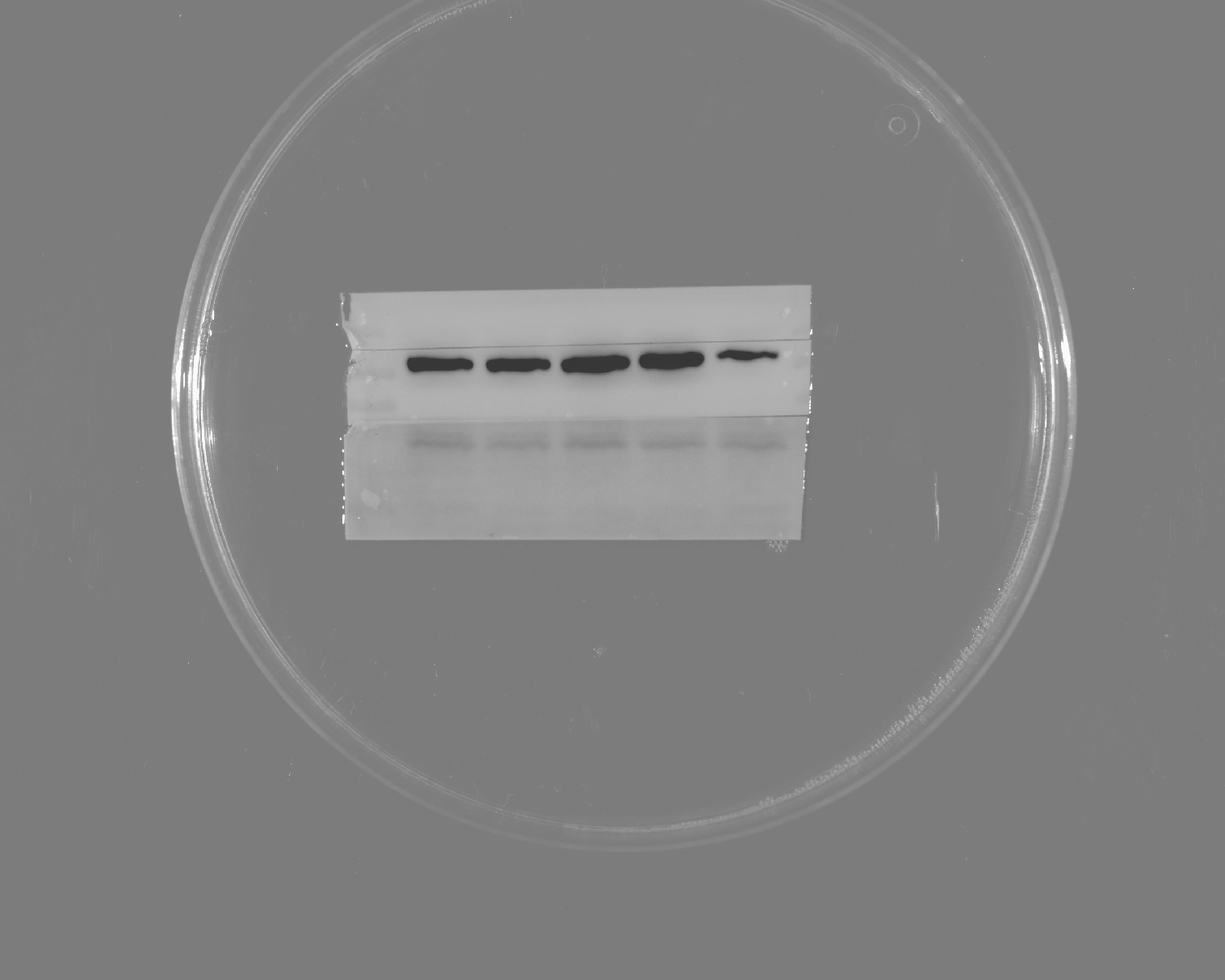

Supplement: Supplementary file 3 [file DataSheet1.ZIP › cladin5/H(2).tif]

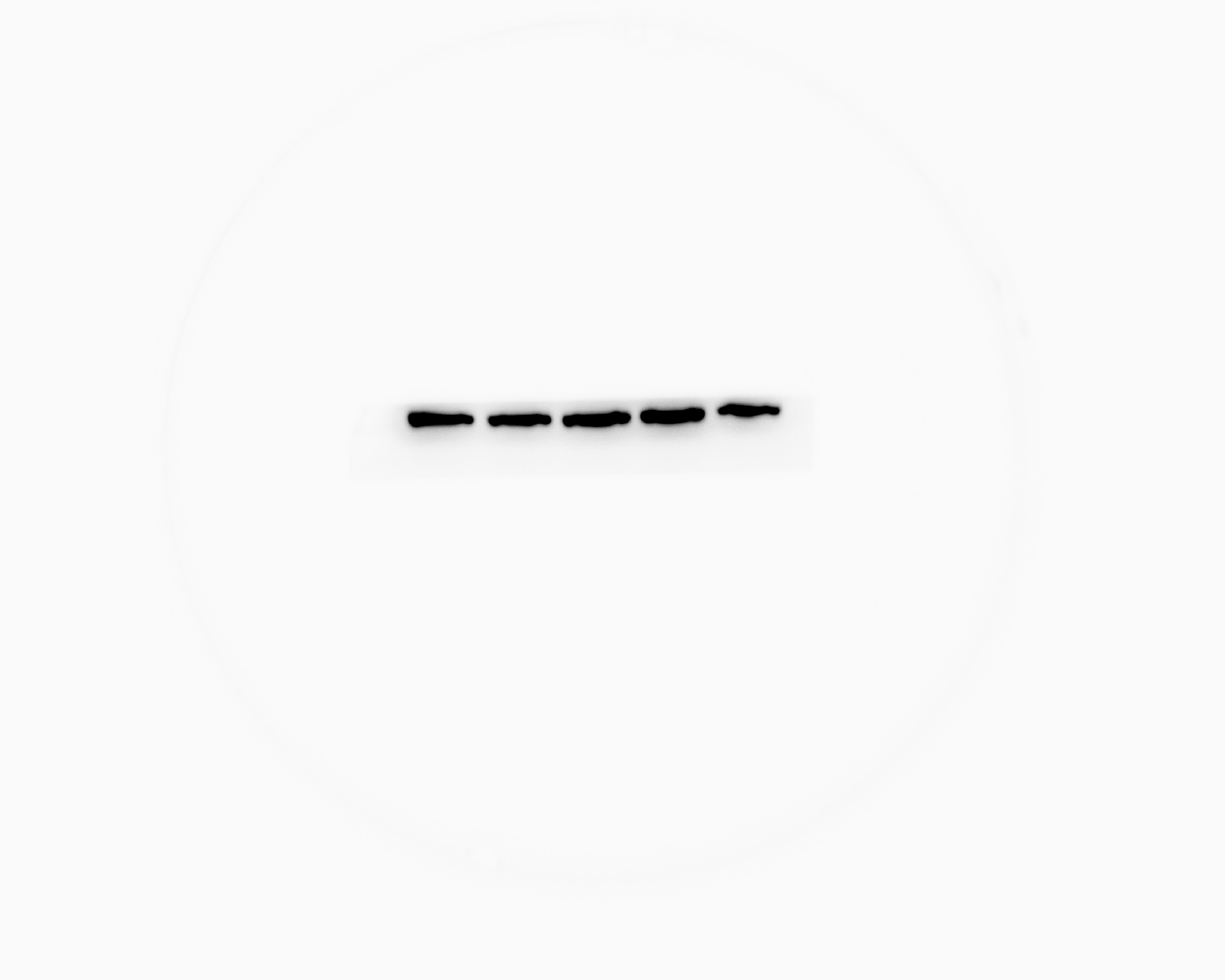

Supplement: Supplementary file 3 [file DataSheet1.ZIP › cladin5/β-actin (1).tif]

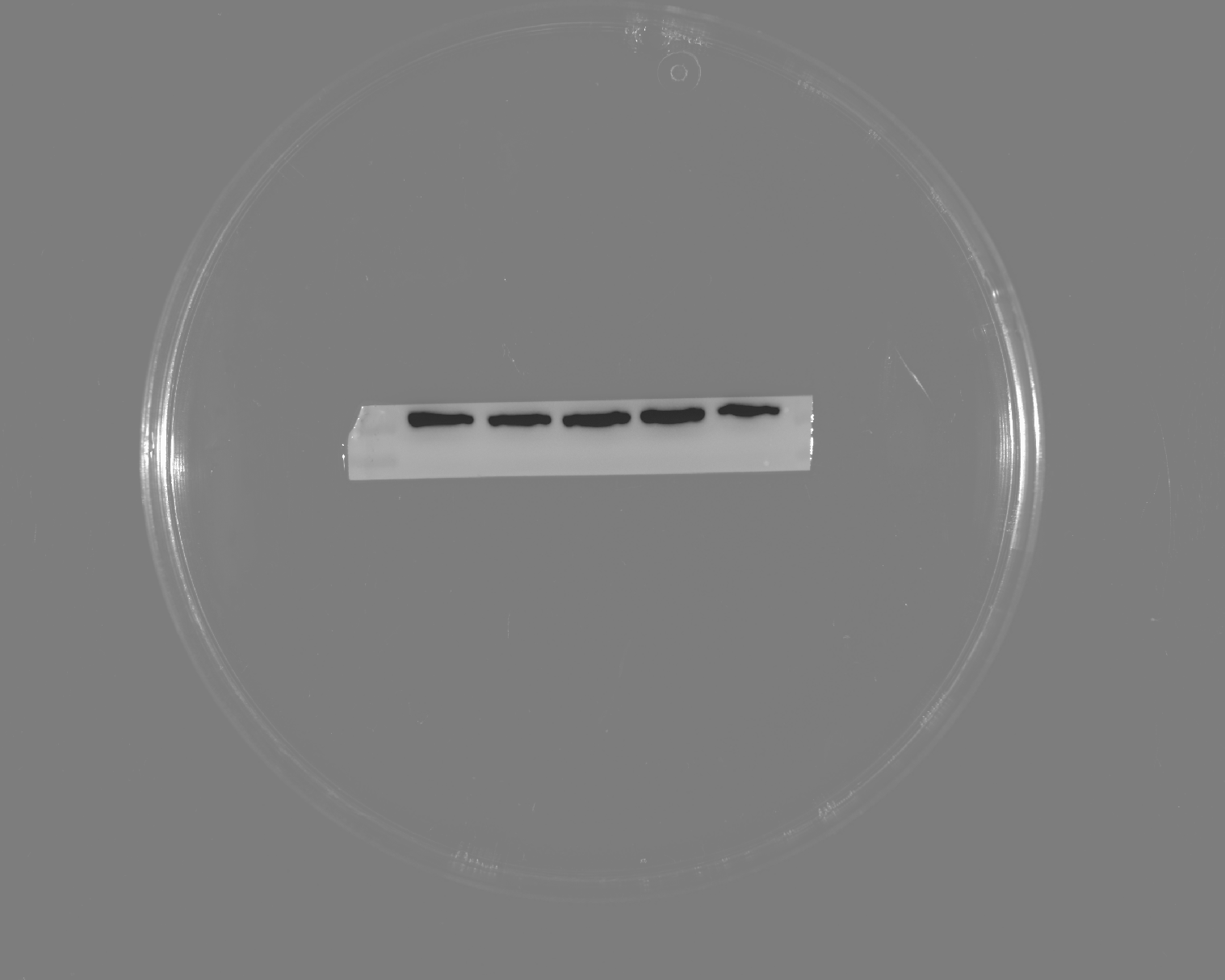

Supplement: Supplementary file 3 [file DataSheet1.ZIP › cladin5/β-actin(2).tif]

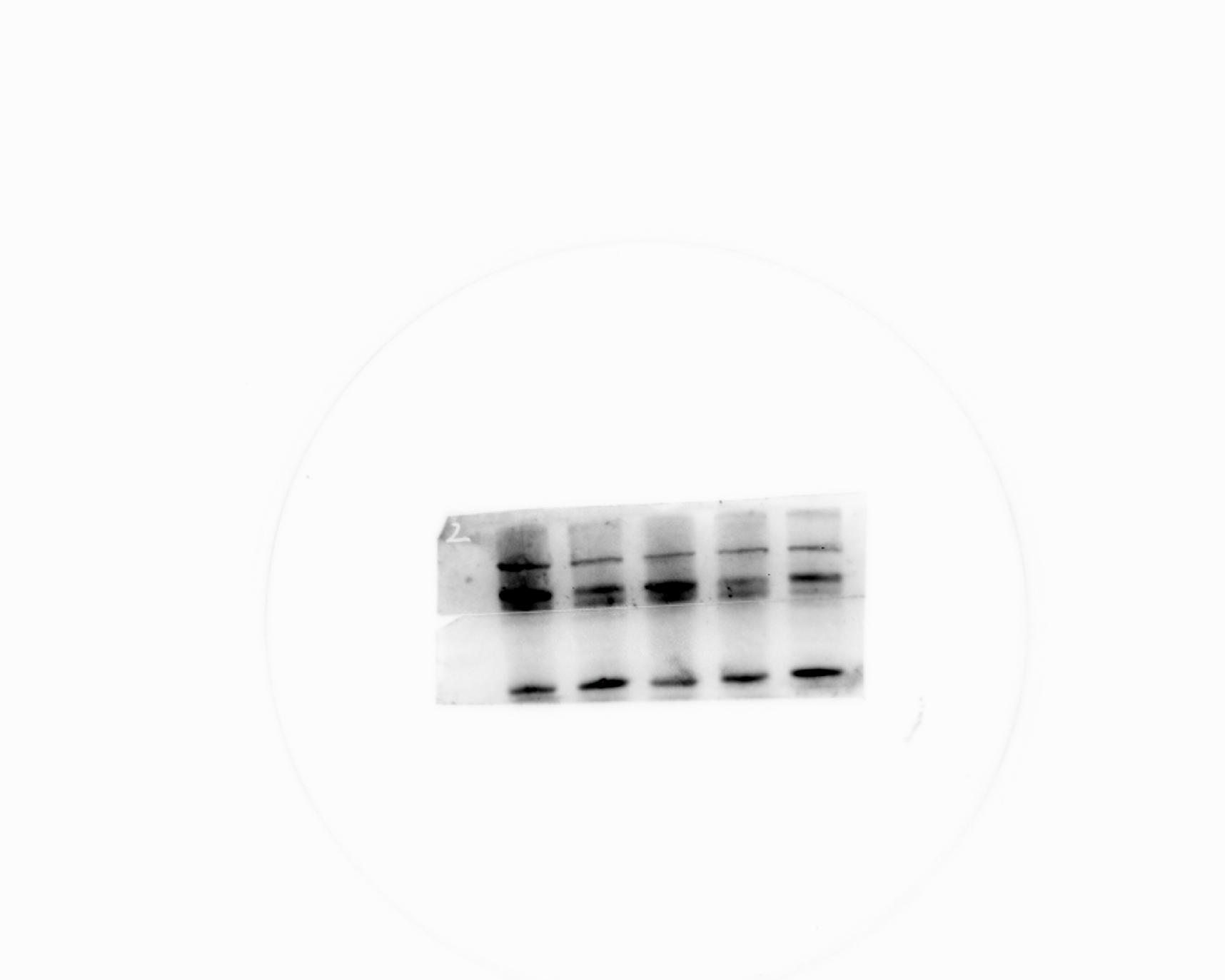

Supplement: Supplementary file 3 [file DataSheet1.ZIP › occ/H (1).tif]

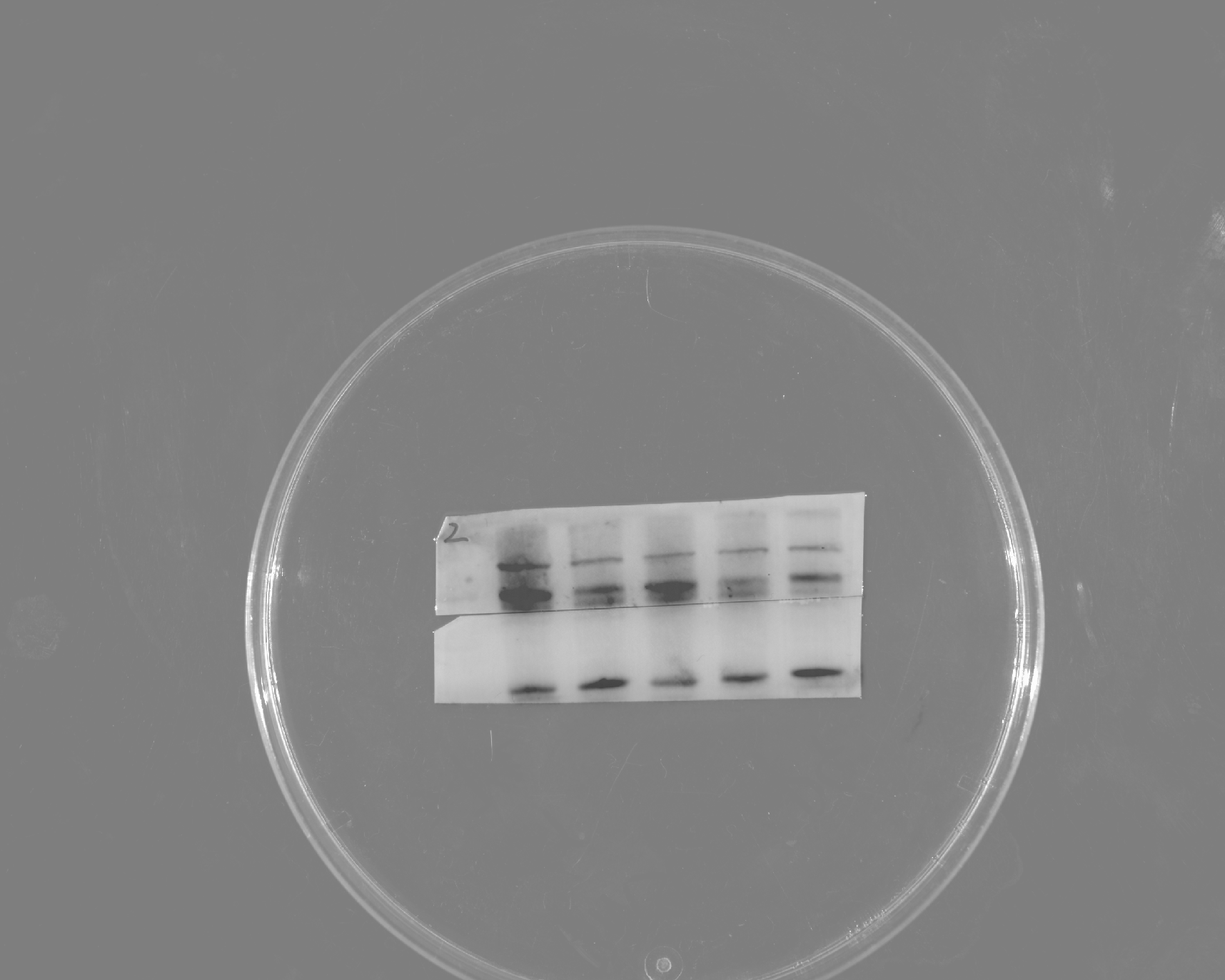

Supplement: Supplementary file 3 [file DataSheet1.ZIP › occ/H (2).tif]

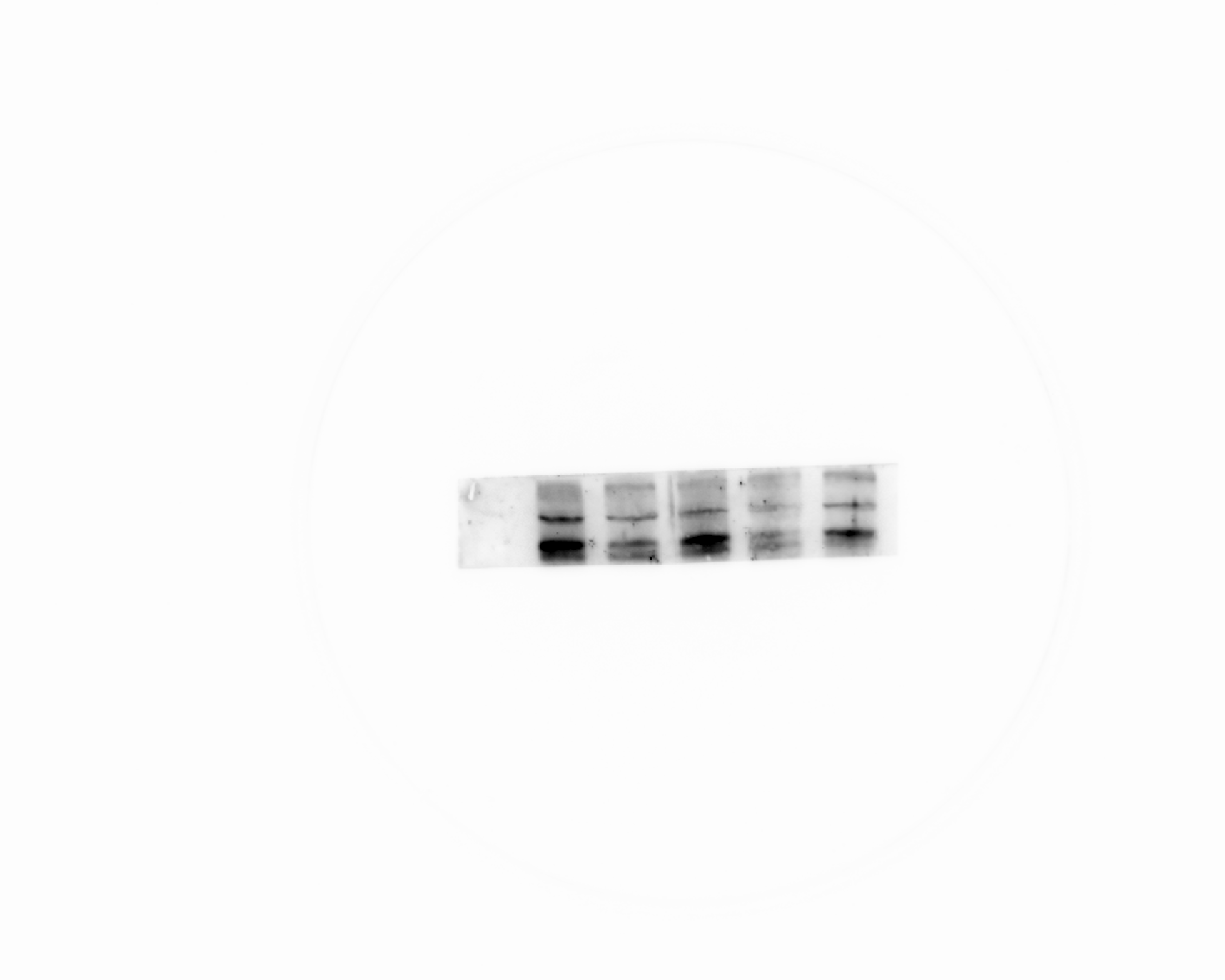

Supplement: Supplementary file 3 [file DataSheet1.ZIP › occ/OCC (1).tif]

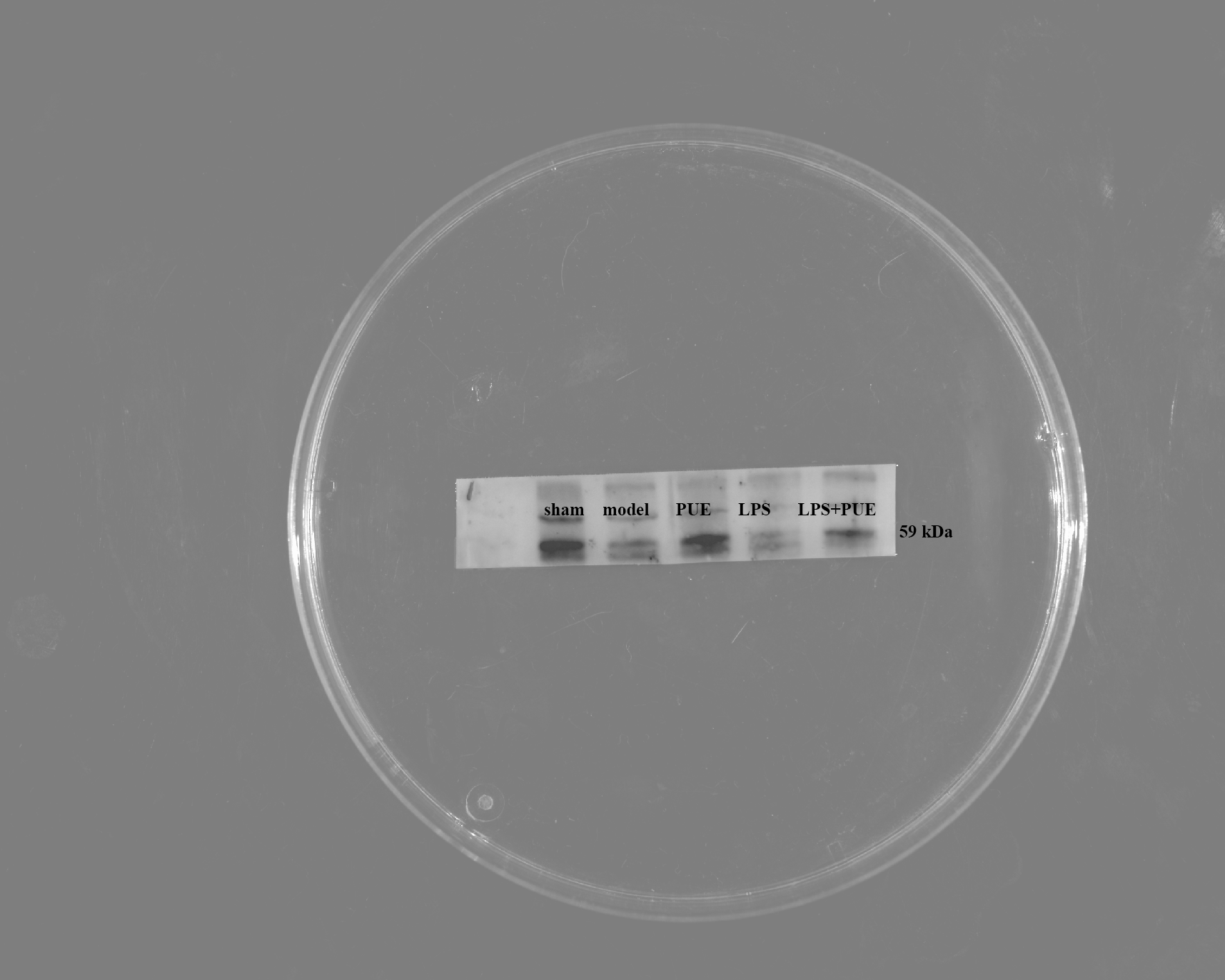

Supplement: Supplementary file 3 [file DataSheet1.ZIP › occ/OCC (2).tif]

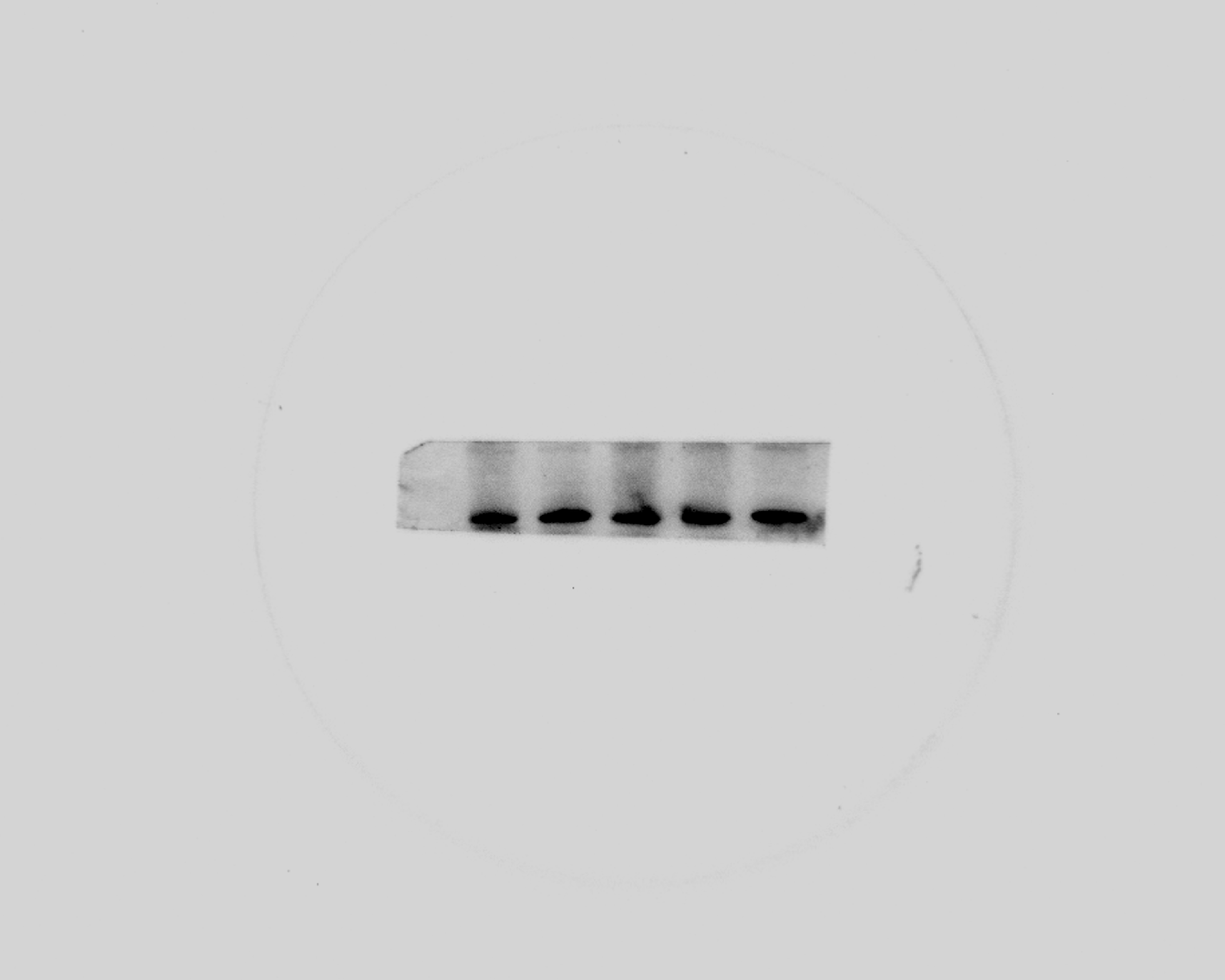

Supplement: Supplementary file 3 [file DataSheet1.ZIP › occ/β-actin (1).tif]

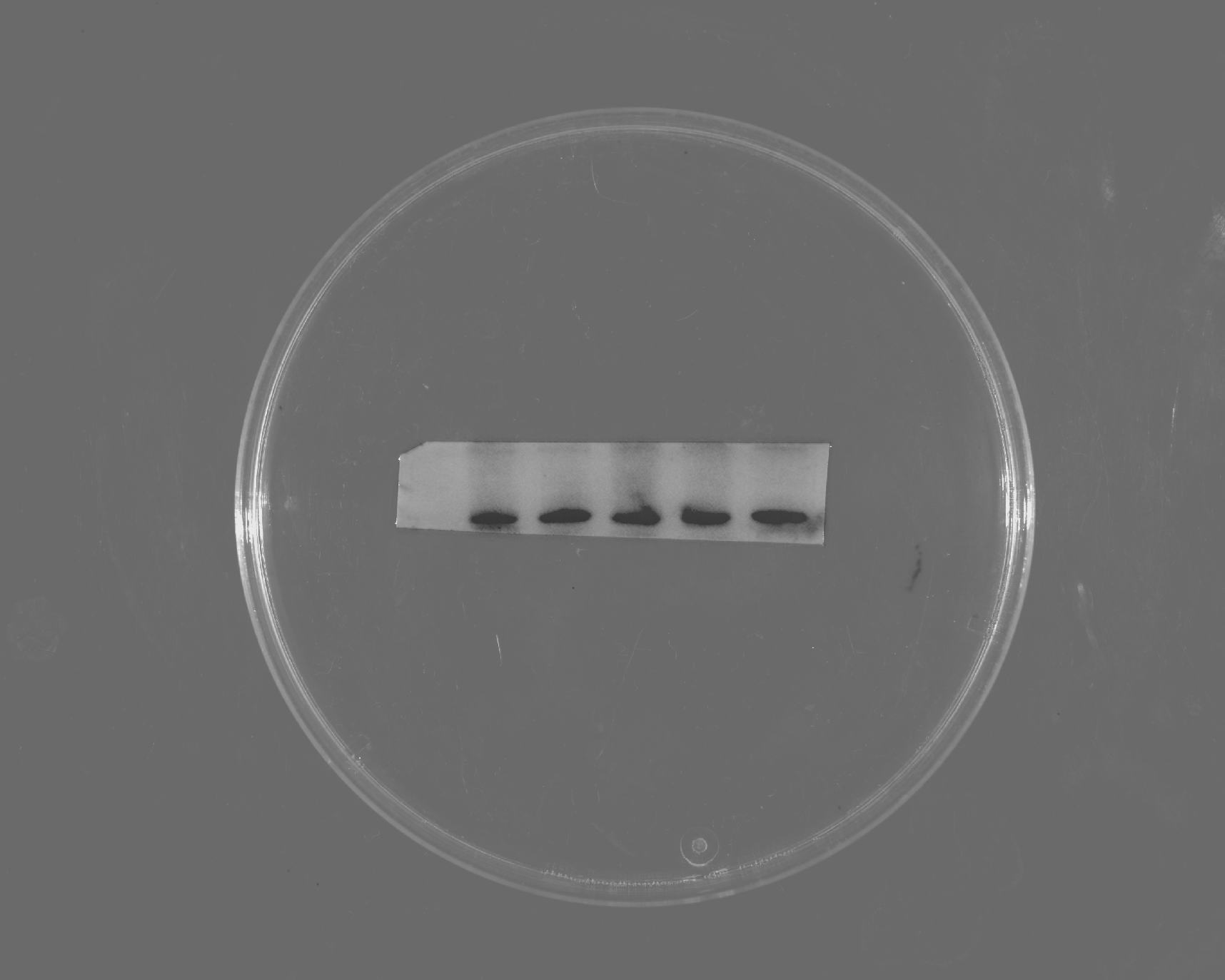

Supplement: Supplementary file 3 [file DataSheet1.ZIP › occ/β-actin (2).tif]

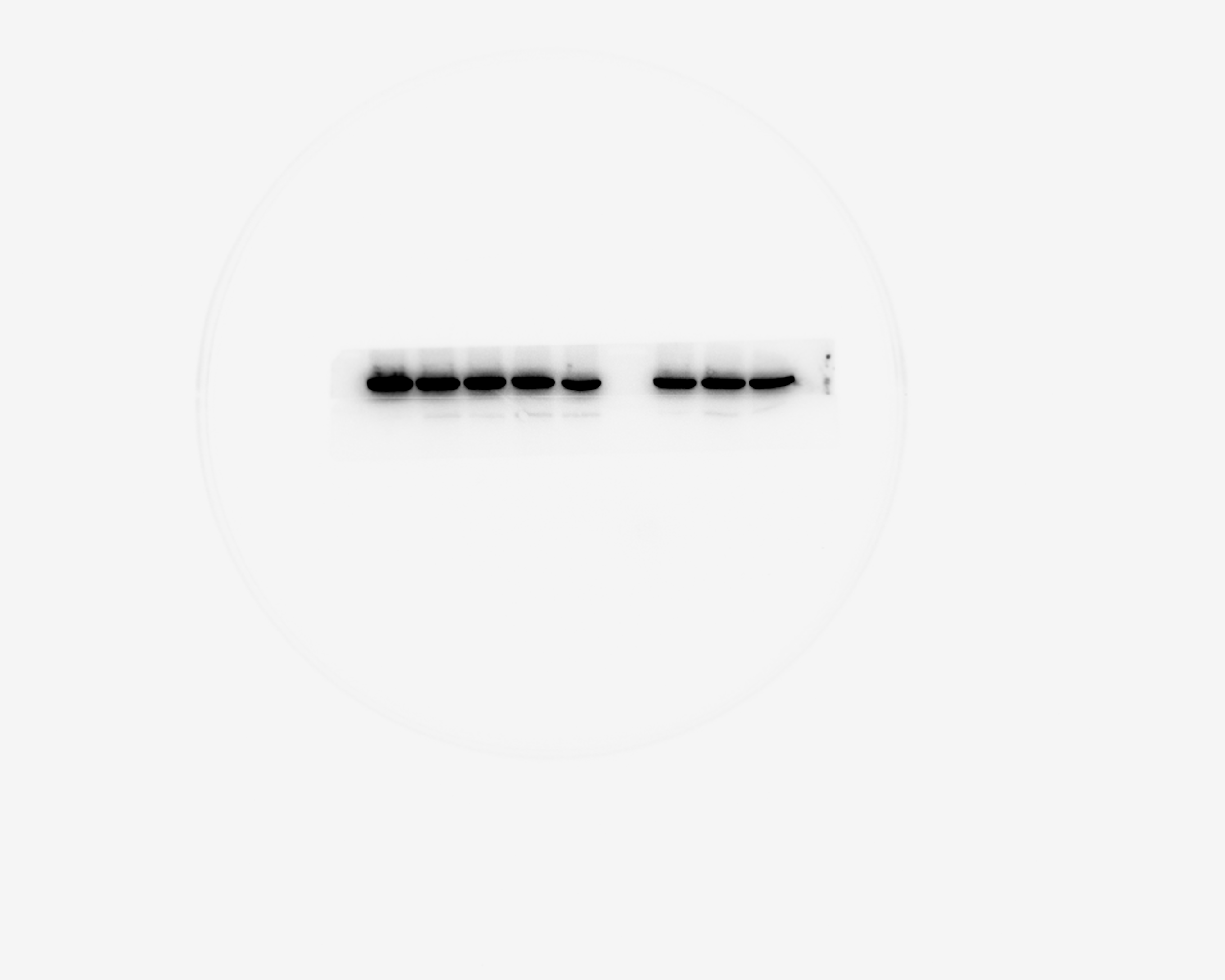

Supplement: Supplementary file 3 [file DataSheet1.ZIP › TNFα/H (1).tif]

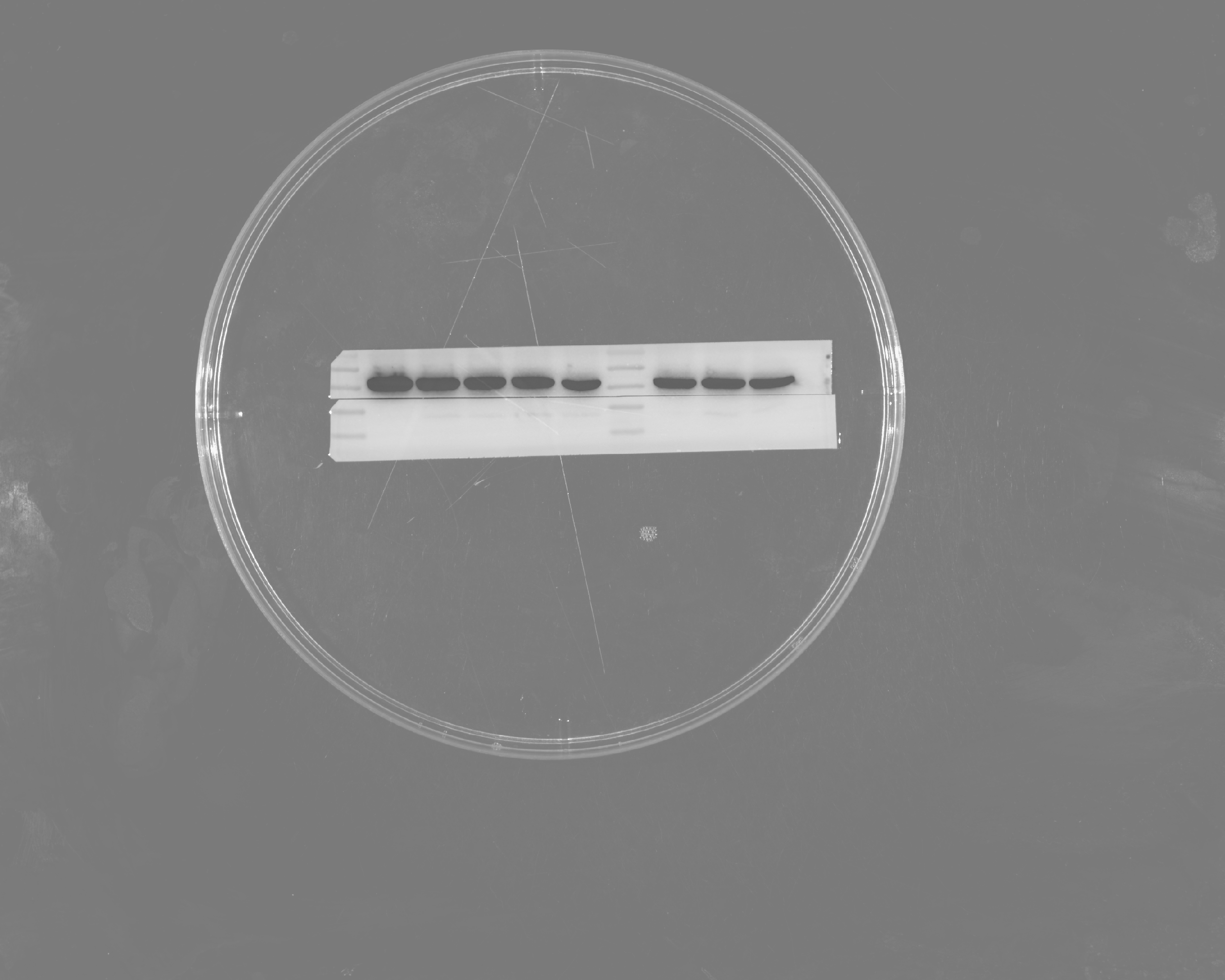

Supplement: Supplementary file 3 [file DataSheet1.ZIP › TNFα/H (2).tif]

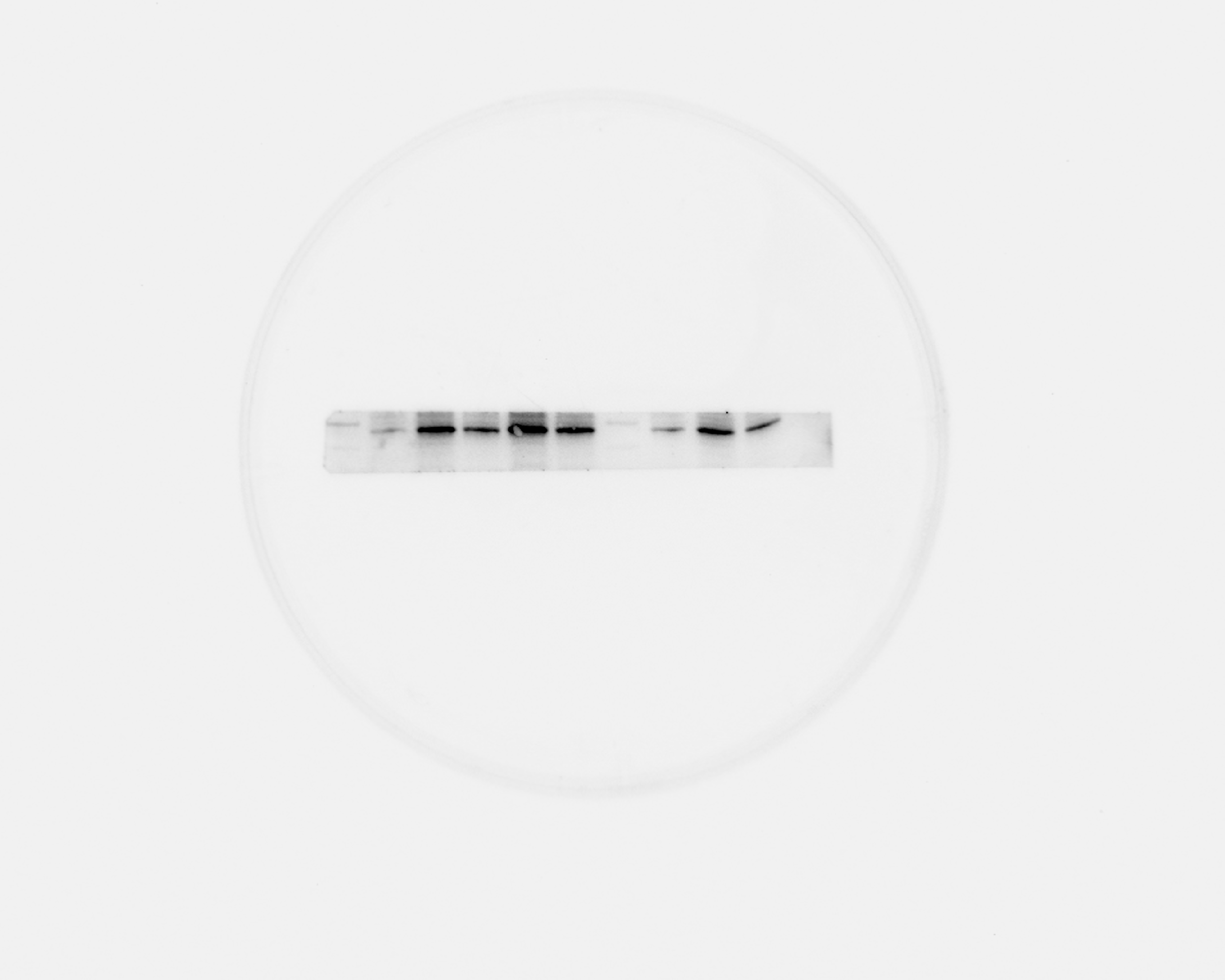

Supplement: Supplementary file 3 [file DataSheet1.ZIP › TNFα/TNFα (1).tif]

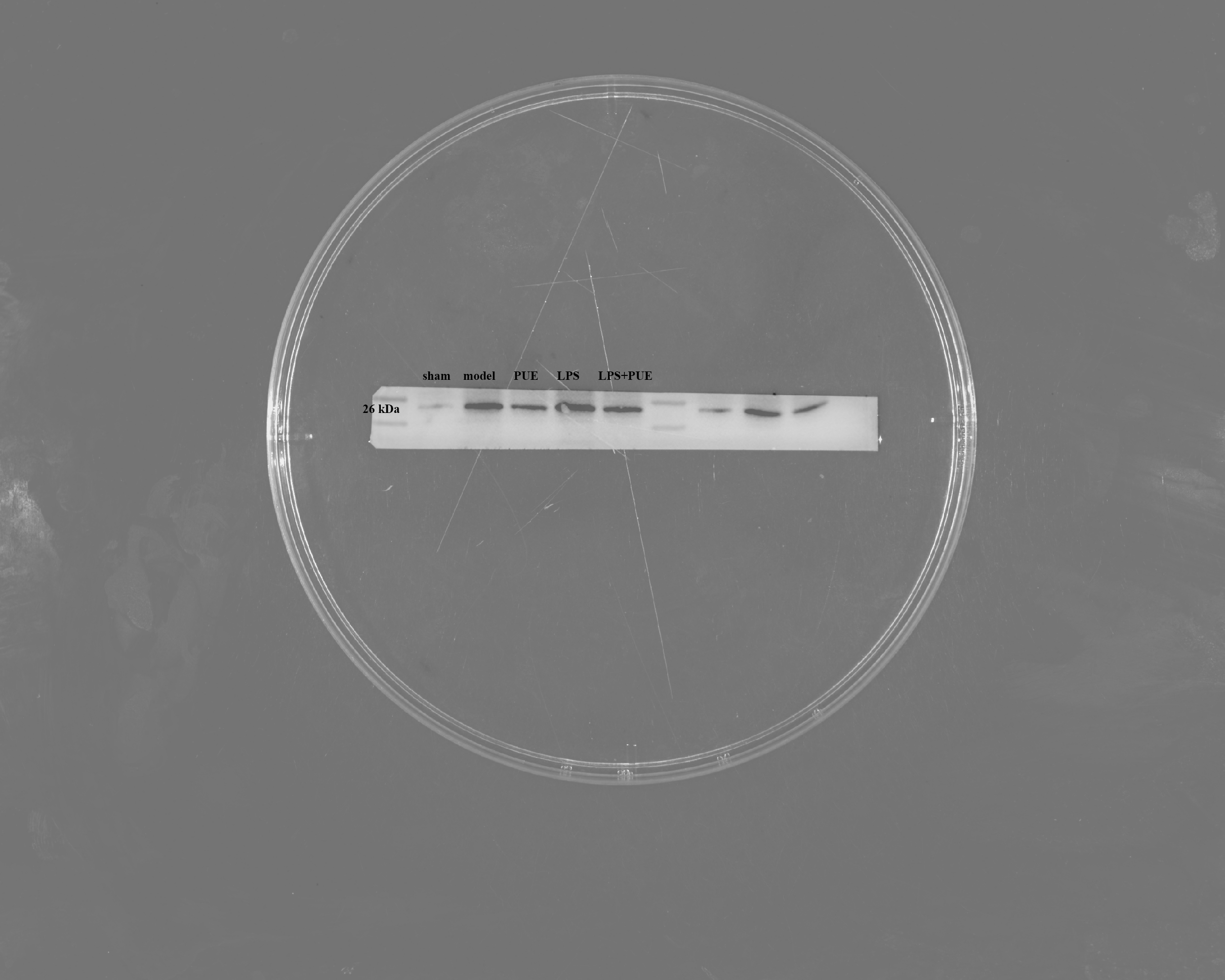

Supplement: Supplementary file 3 [file DataSheet1.ZIP › TNFα/TNFα (2).tif]

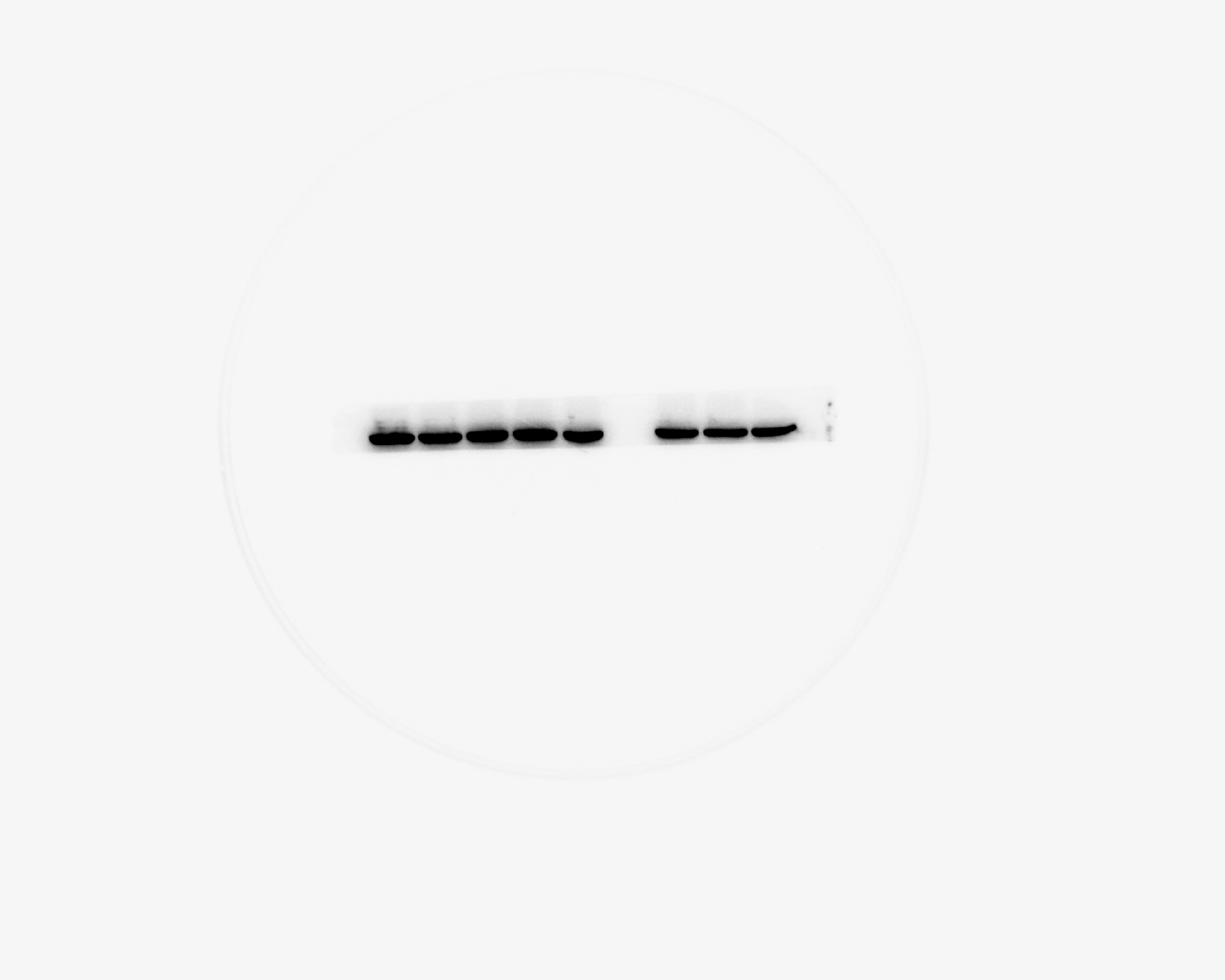

Supplement: Supplementary file 3 [file DataSheet1.ZIP › TNFα/β-actin (1).tif]

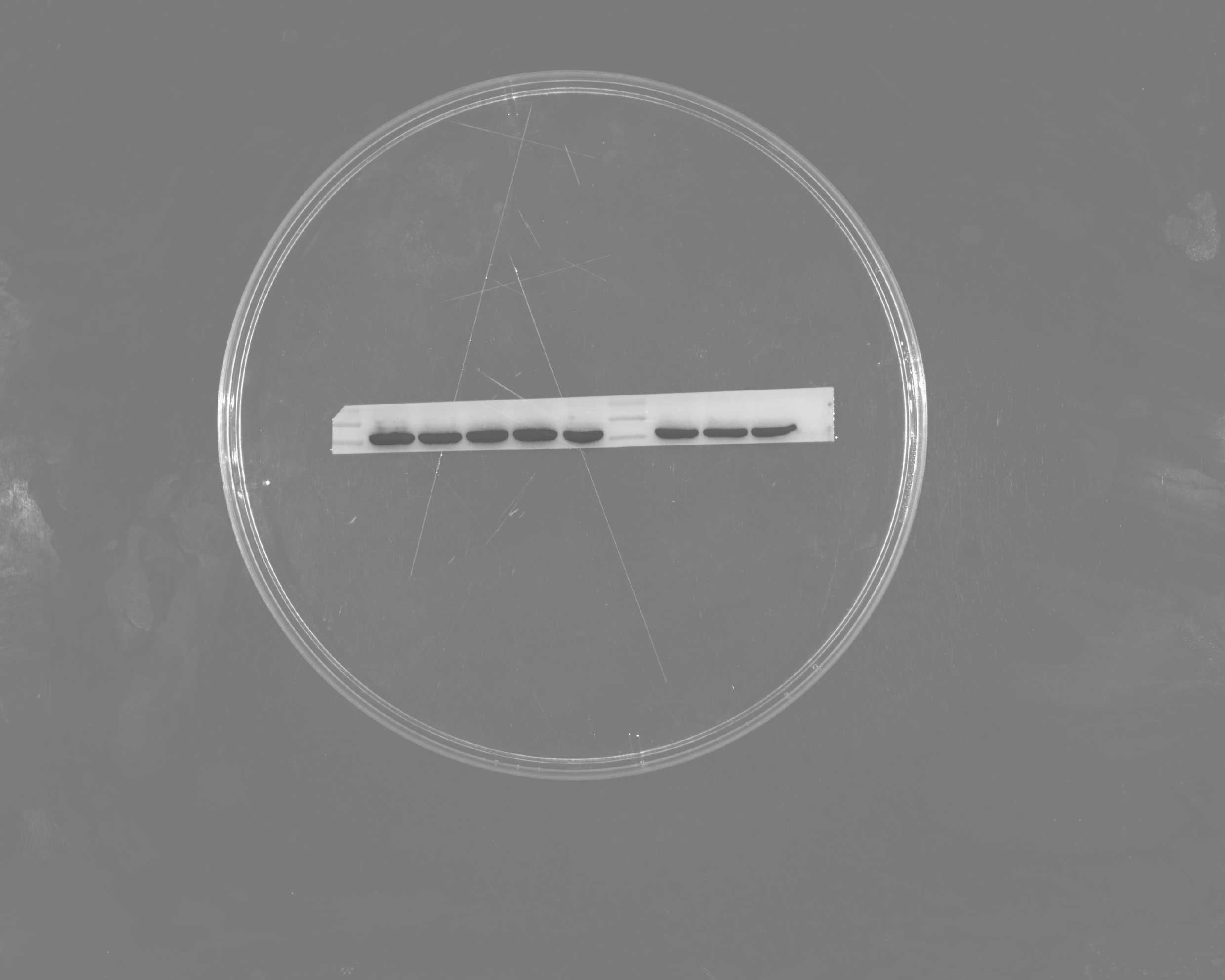

Supplement: Supplementary file 3 [file DataSheet1.ZIP › TNFα/β-actin (2).tif]

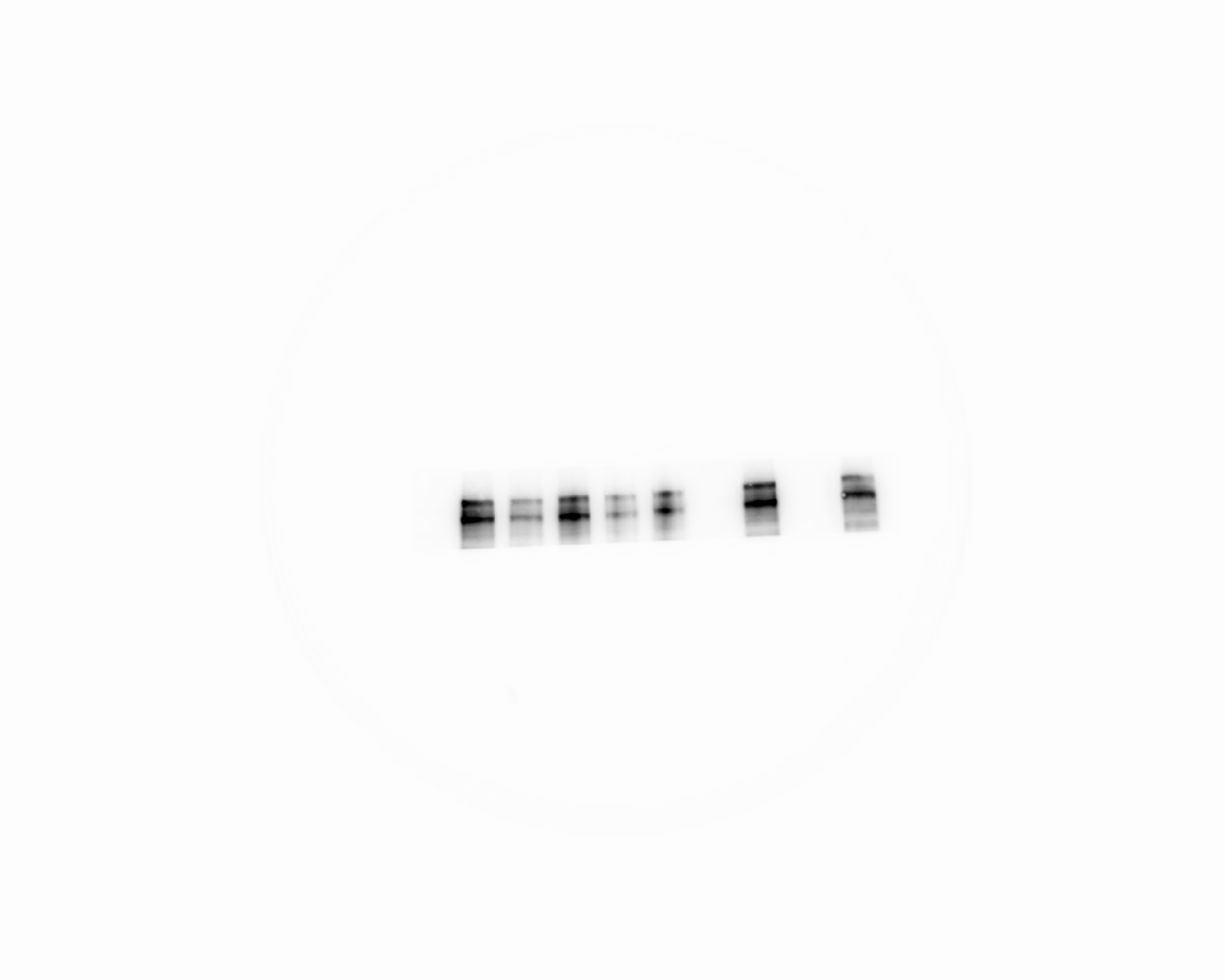

Supplement: Supplementary file 3 [file DataSheet1.ZIP › zo1/ZO1 (1).tif]

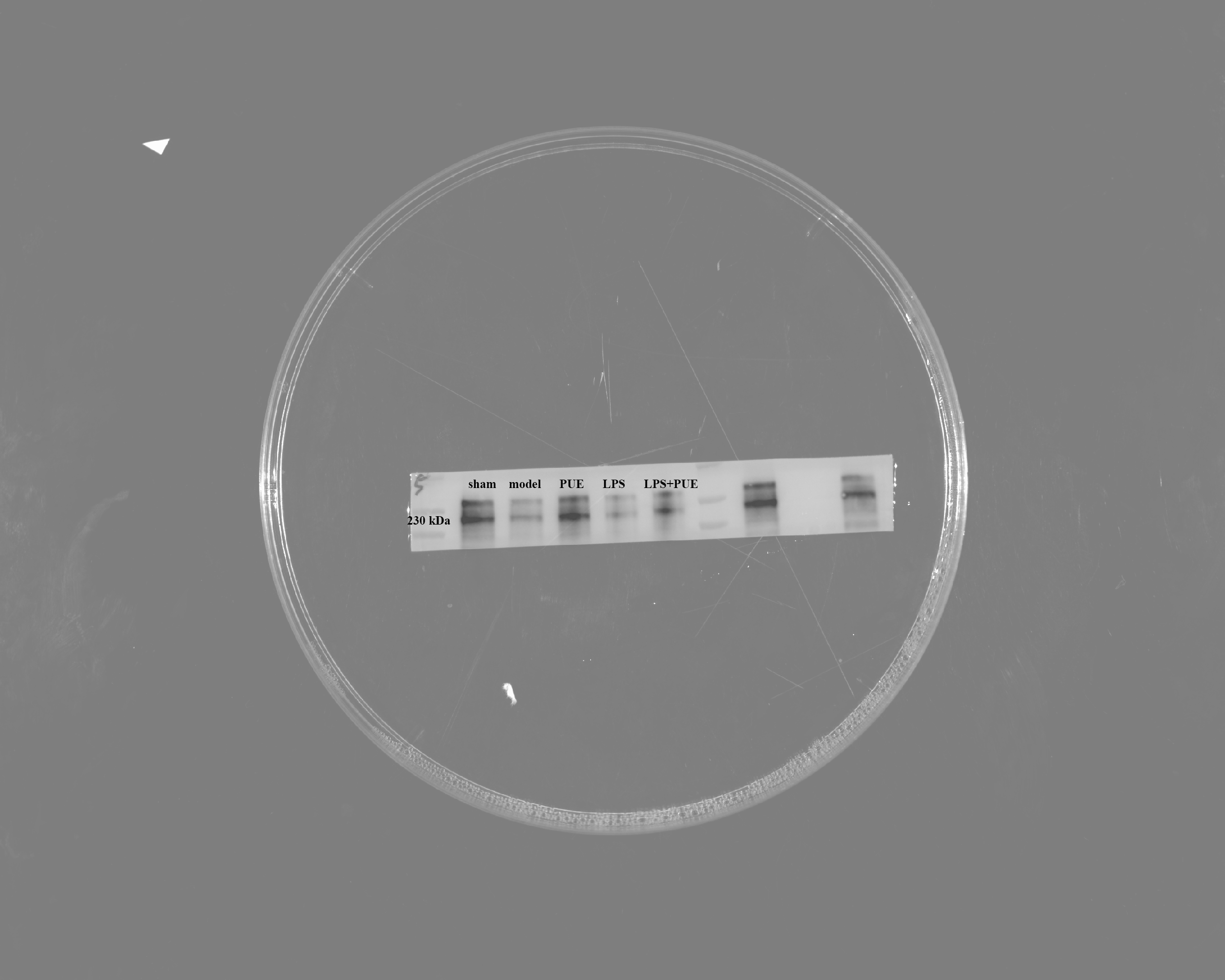

Supplement: Supplementary file 3 [file DataSheet1.ZIP › zo1/ZO1 (2).tif]

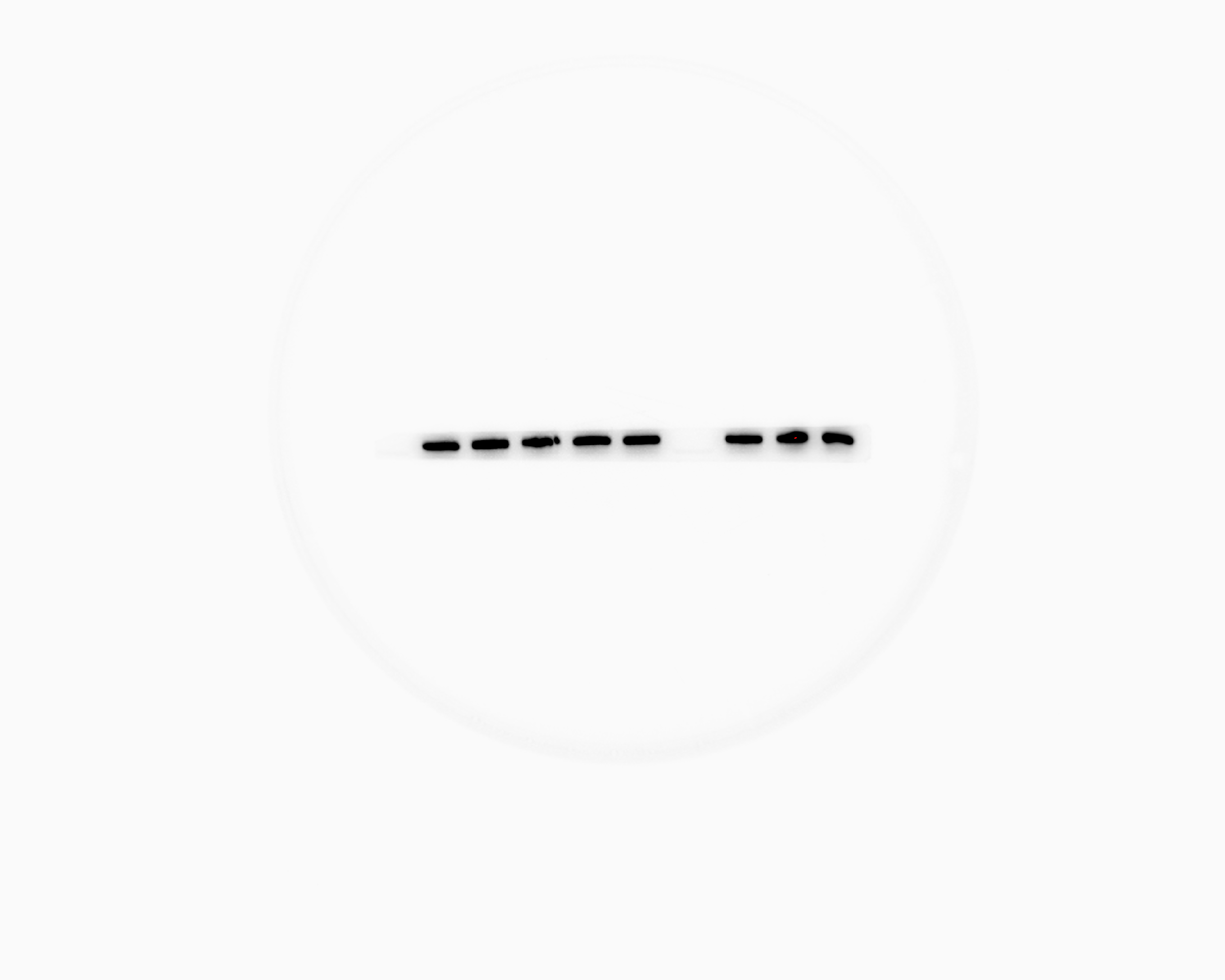

Supplement: Supplementary file 3 [file DataSheet1.ZIP › zo1/zo1(β-actin) (1).tif]

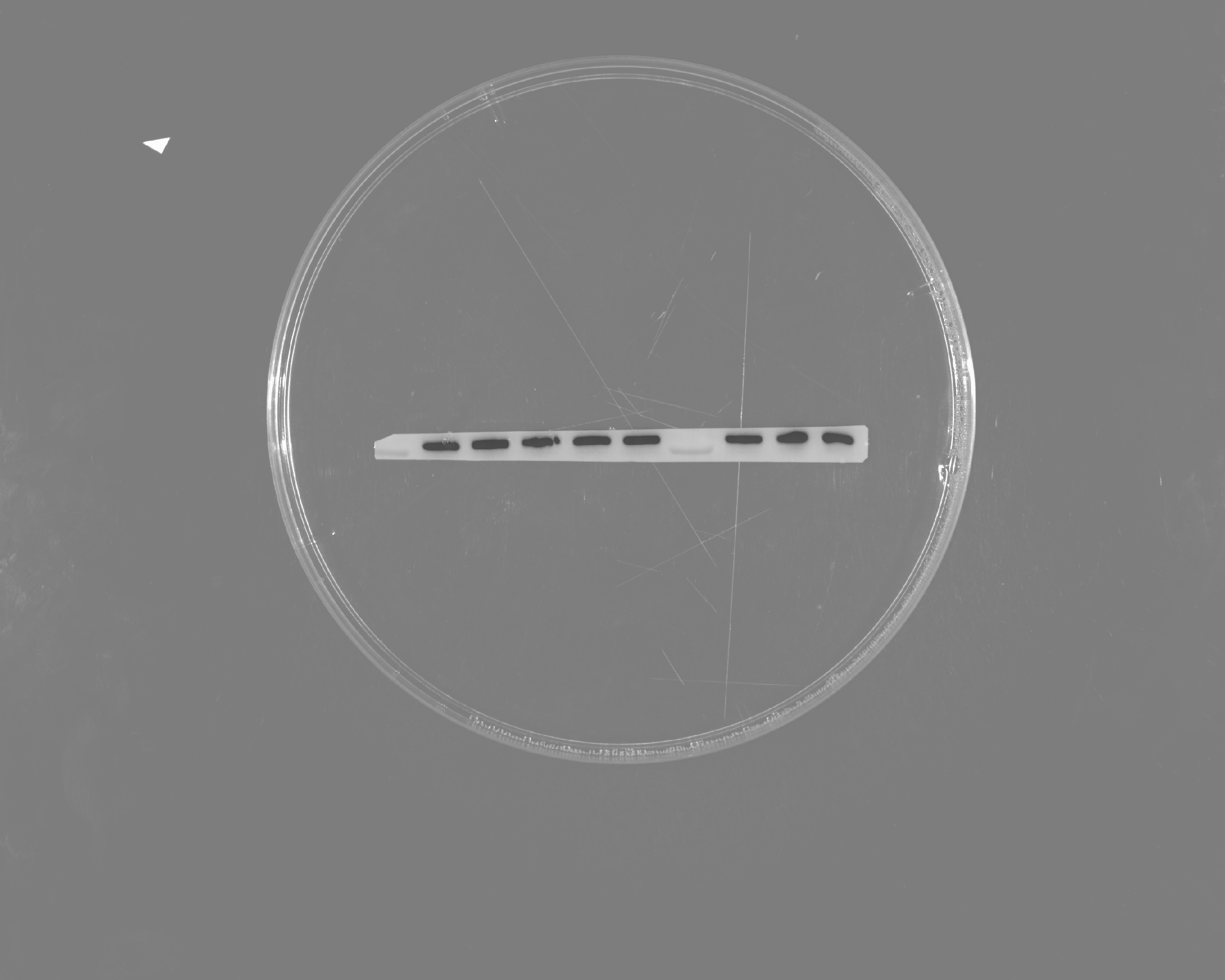

Supplement: Supplementary file 3 [file DataSheet1.ZIP › zo1/zo1(β-actin) (2).tif]

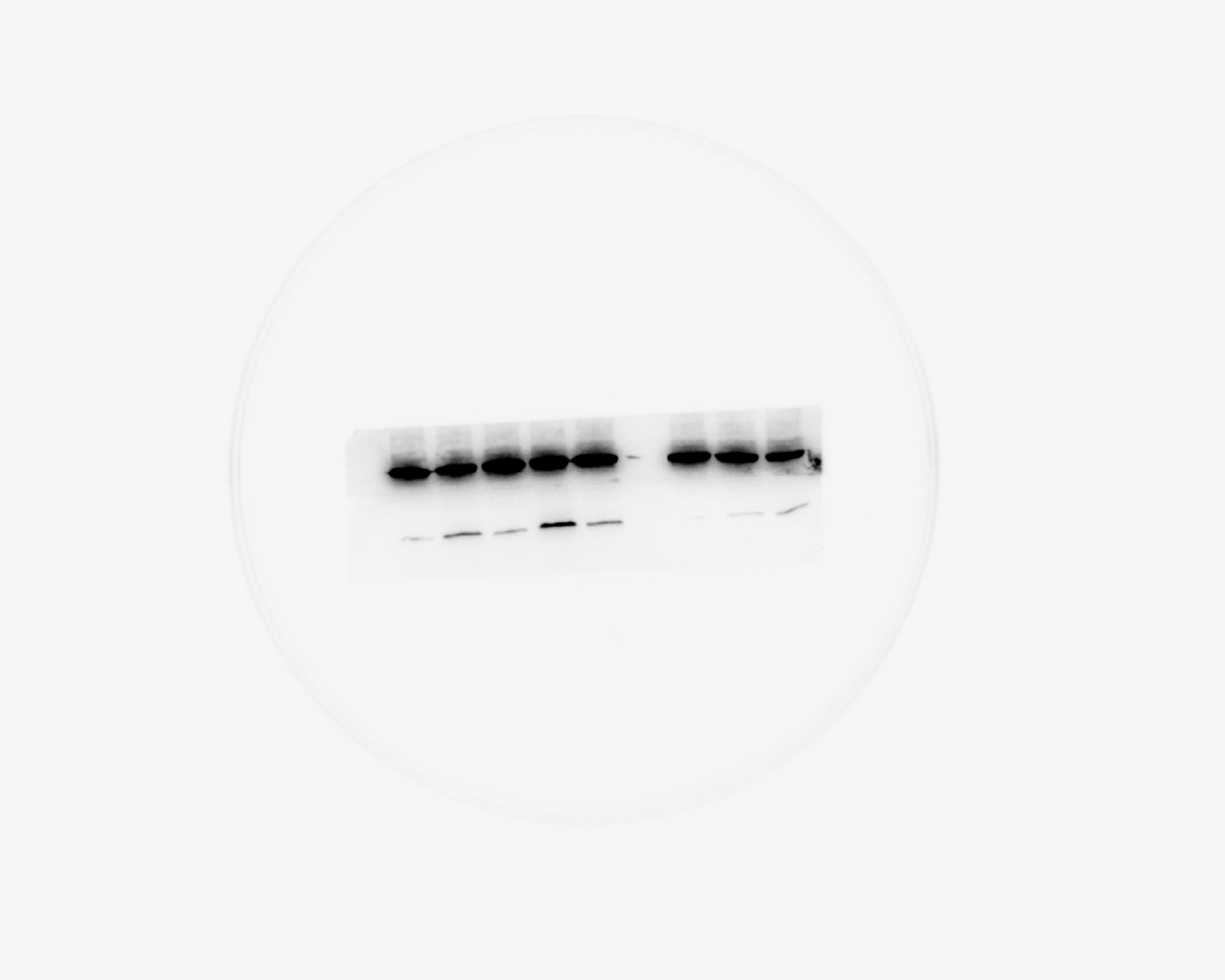

Supplement: Supplementary file 4 [file DataSheet2.ZIP › IL1β/H (1).tif]

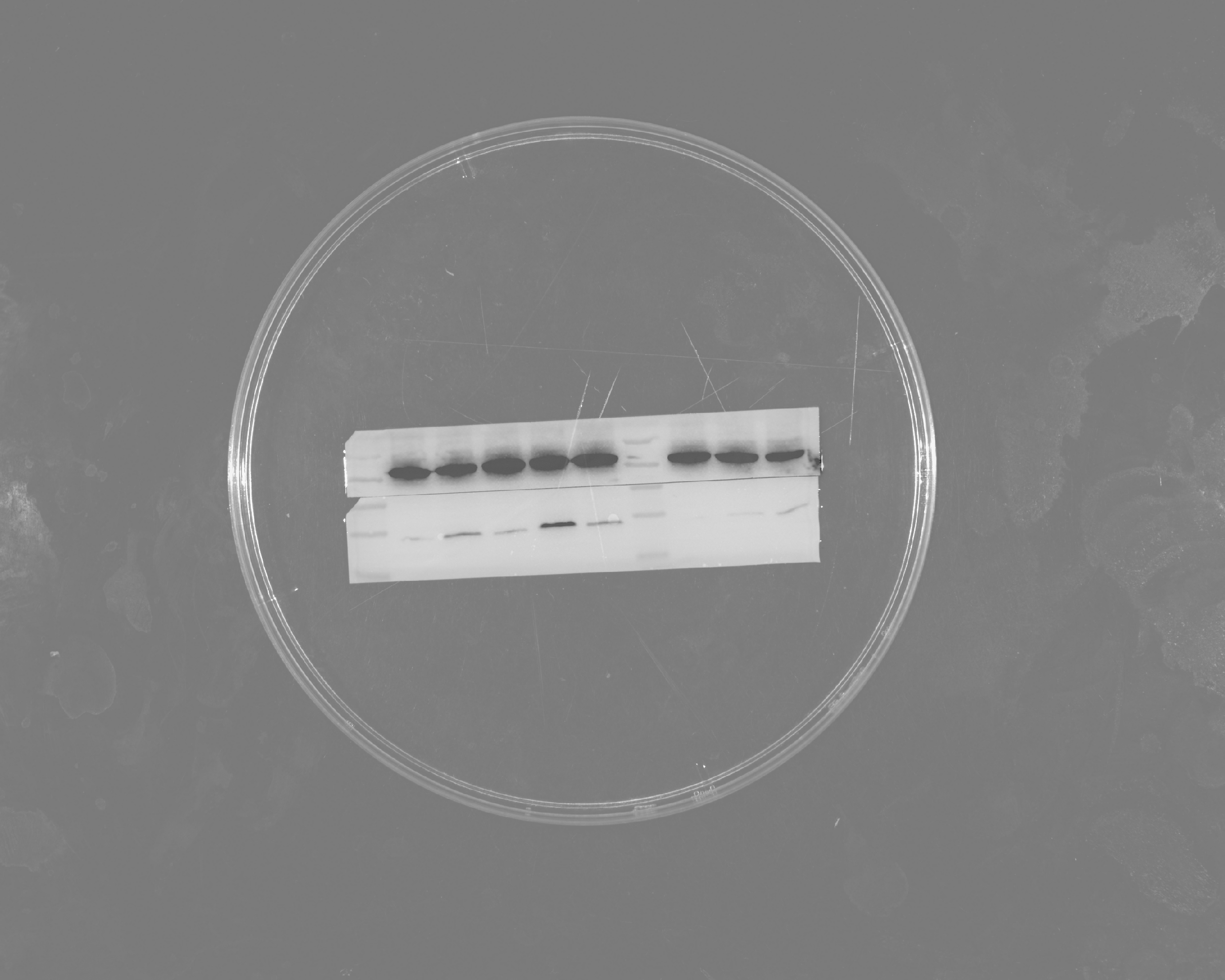

Supplement: Supplementary file 4 [file DataSheet2.ZIP › IL1β/H (2).tif]

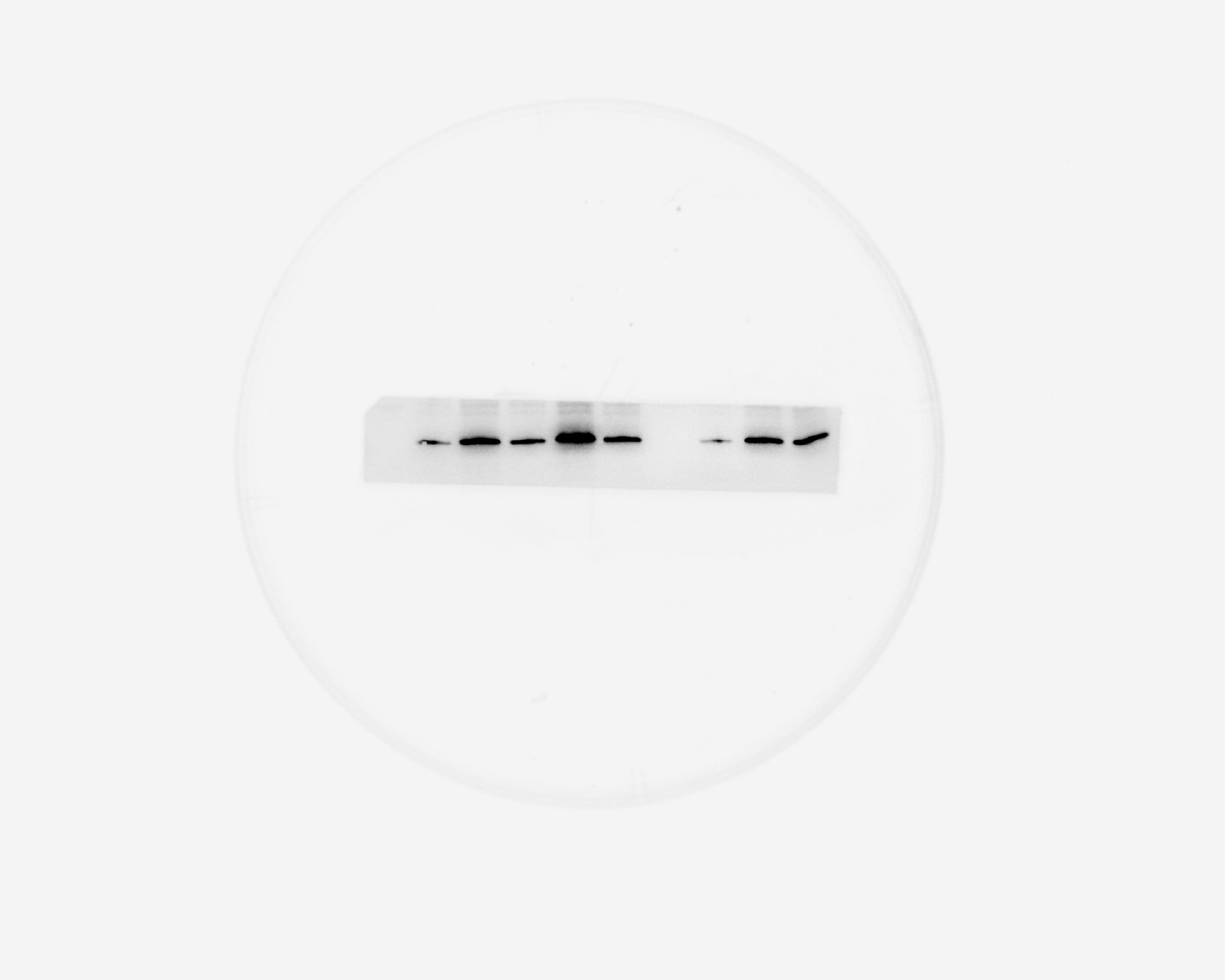

Supplement: Supplementary file 4 [file DataSheet2.ZIP › IL1β/IL1β (1).tif]

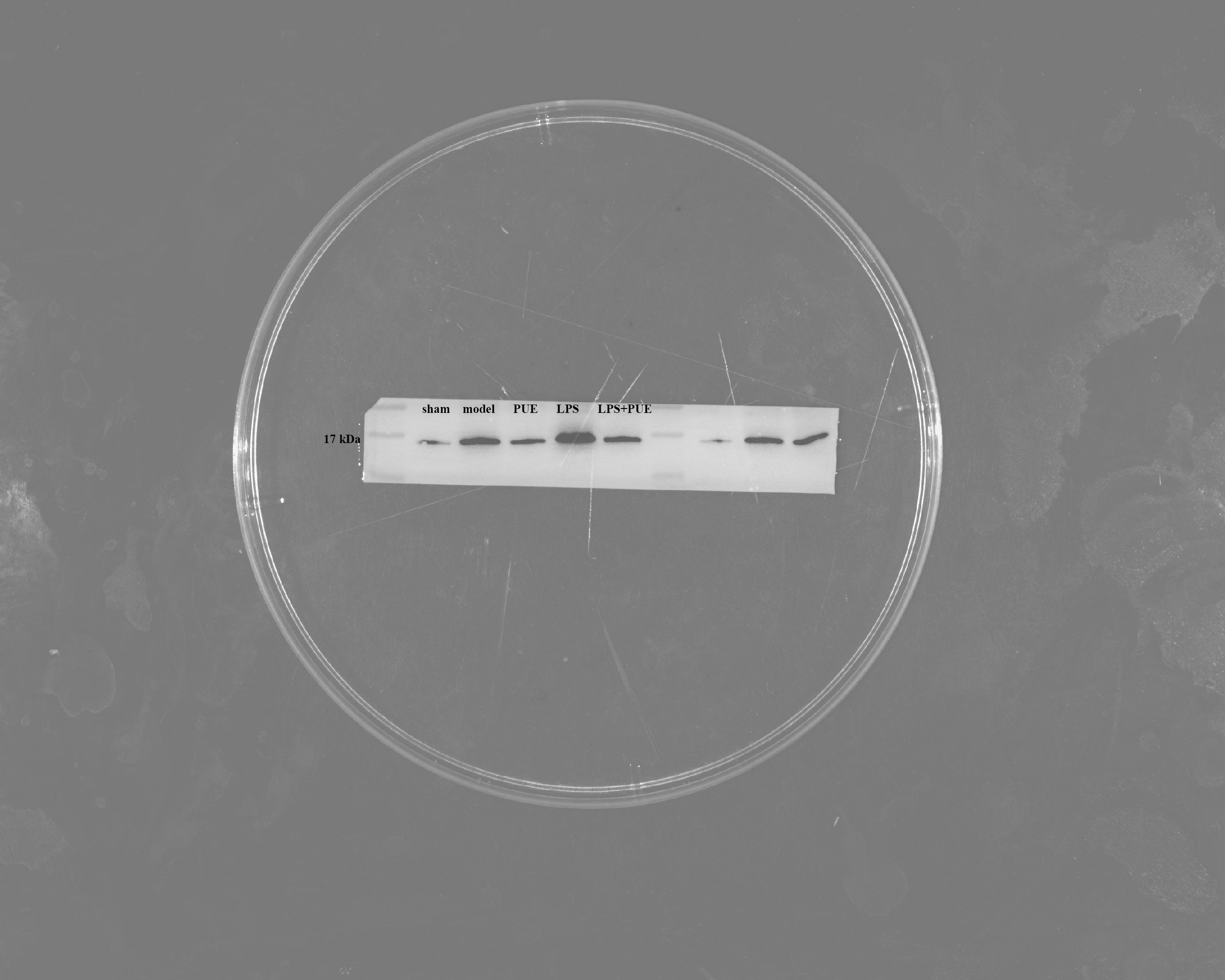

Supplement: Supplementary file 4 [file DataSheet2.ZIP › IL1β/IL1β (2).tif]

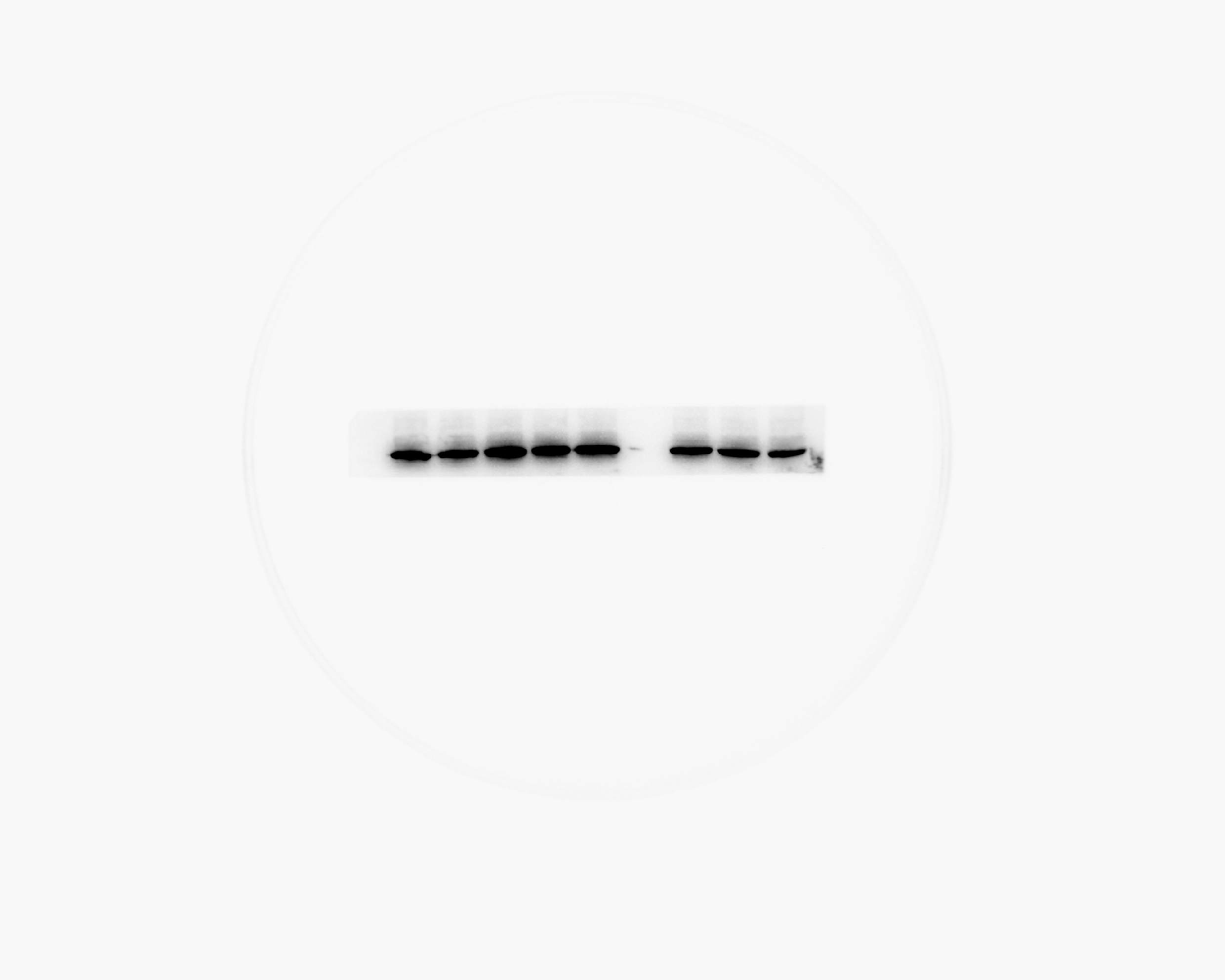

Supplement: Supplementary file 4 [file DataSheet2.ZIP › IL1β/β-actin (1).tif]

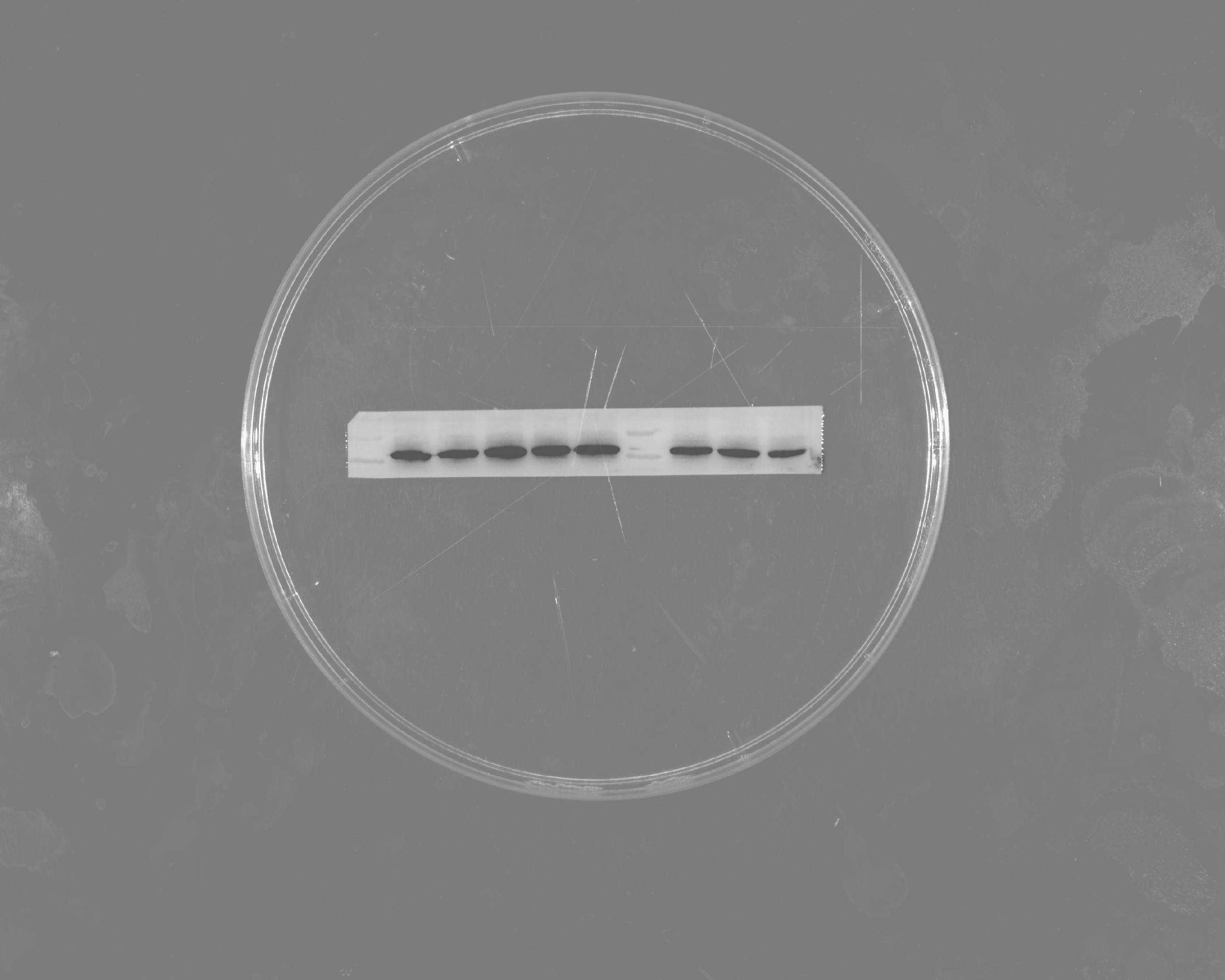

Supplement: Supplementary file 4 [file DataSheet2.ZIP › IL1β/β-actin (2).tif]

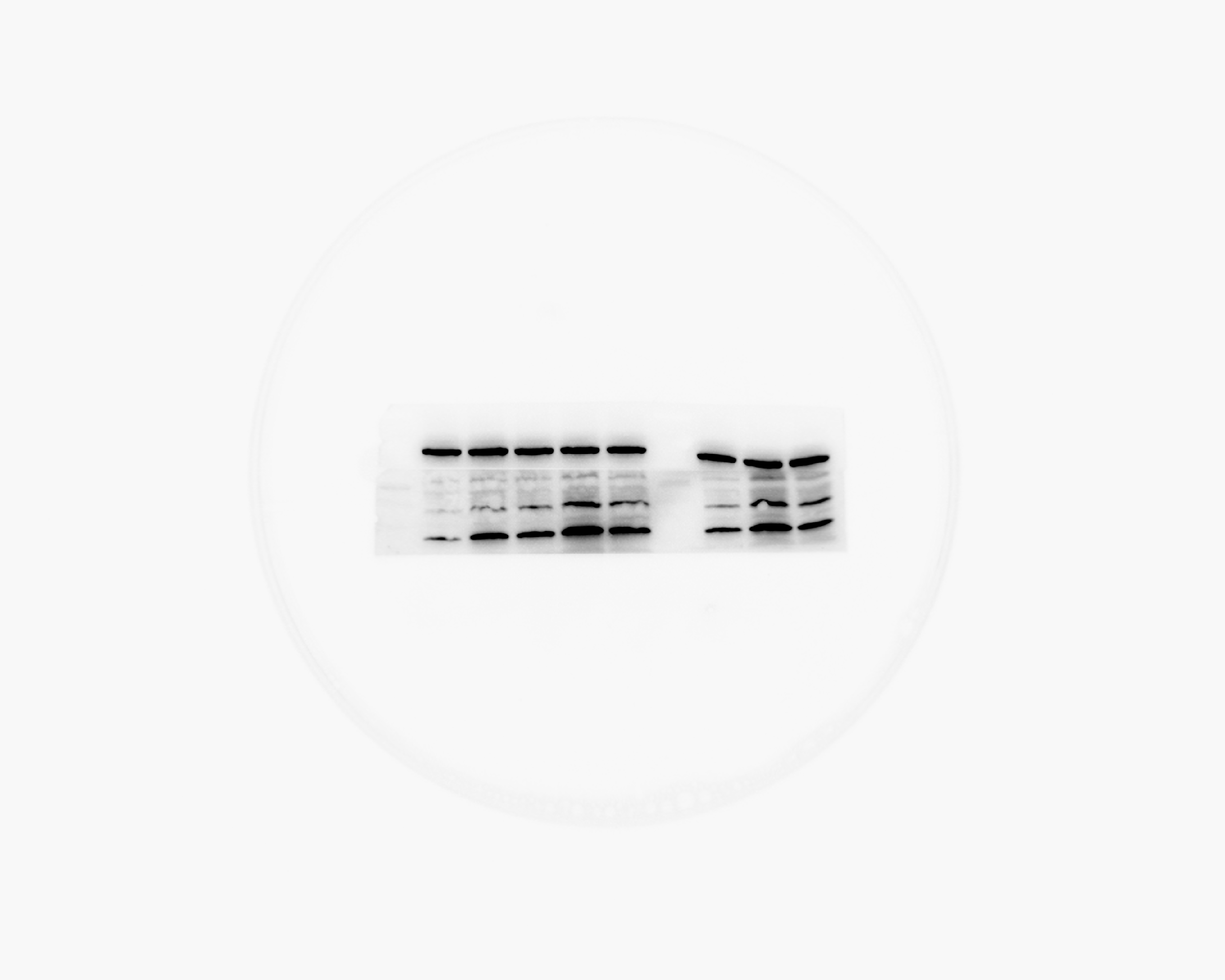

Supplement: Supplementary file 4 [file DataSheet2.ZIP › IL6/H (1).tif]

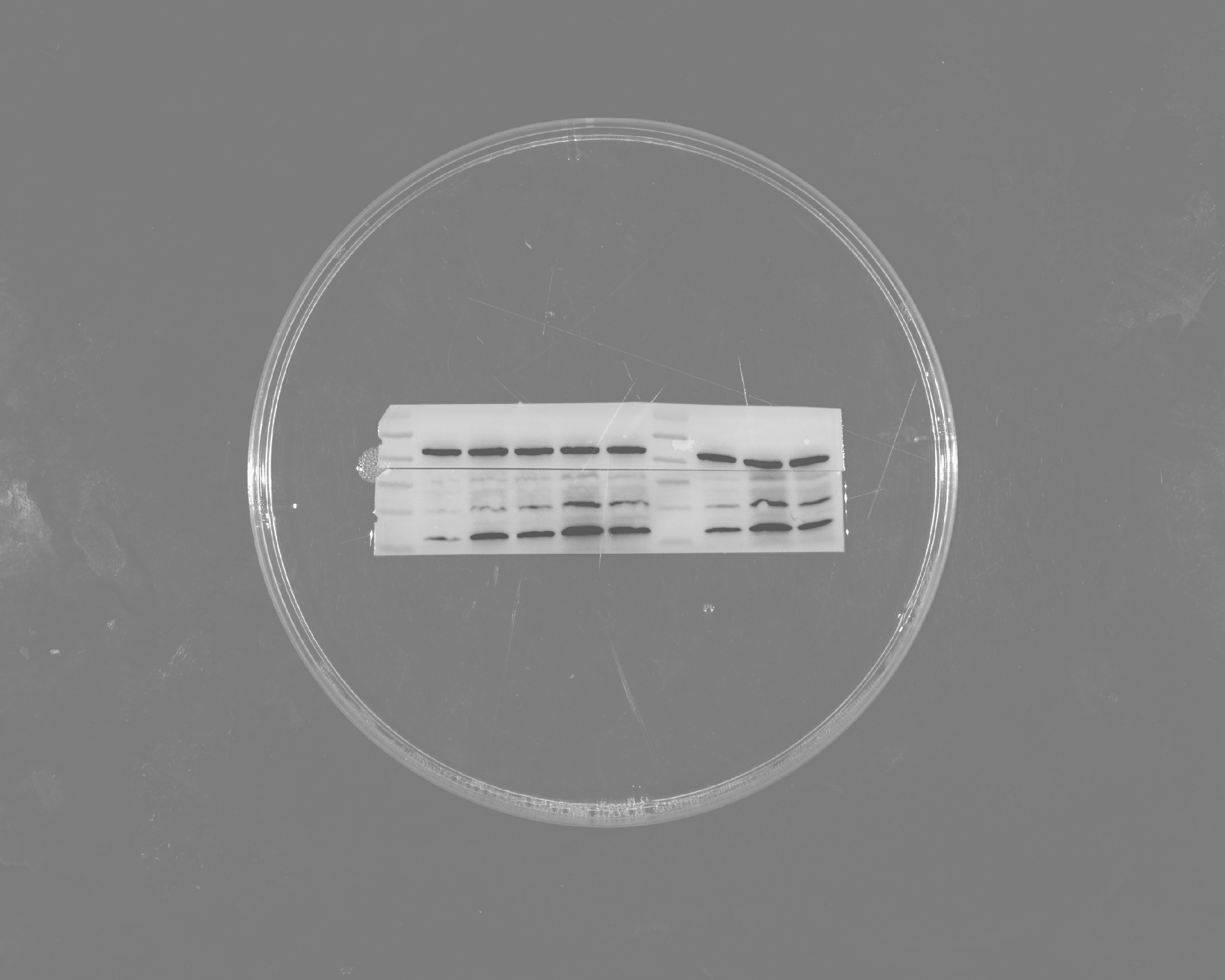

Supplement: Supplementary file 4 [file DataSheet2.ZIP › IL6/H (2).tif]

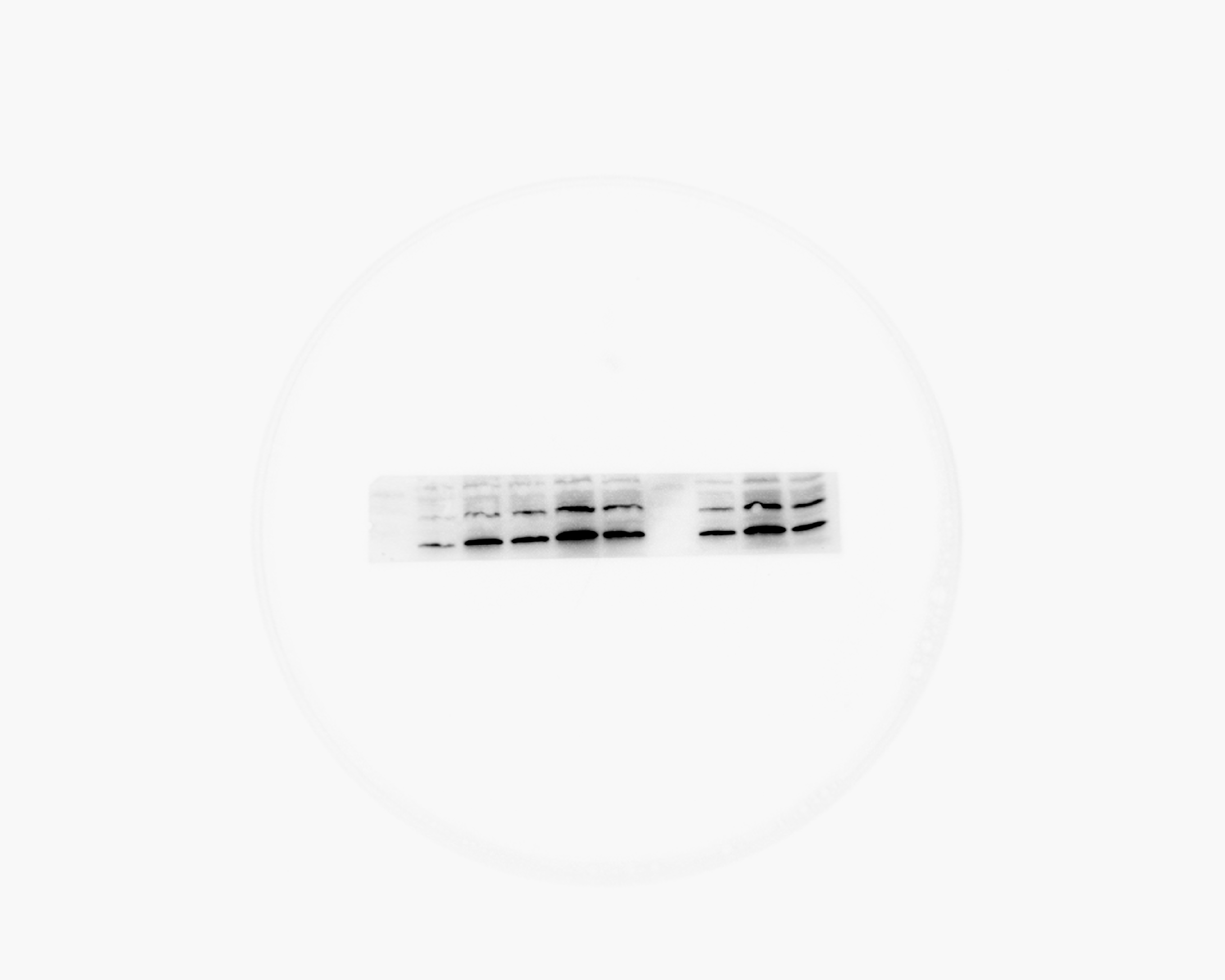

Supplement: Supplementary file 4 [file DataSheet2.ZIP › IL6/IL6 (1).tif]

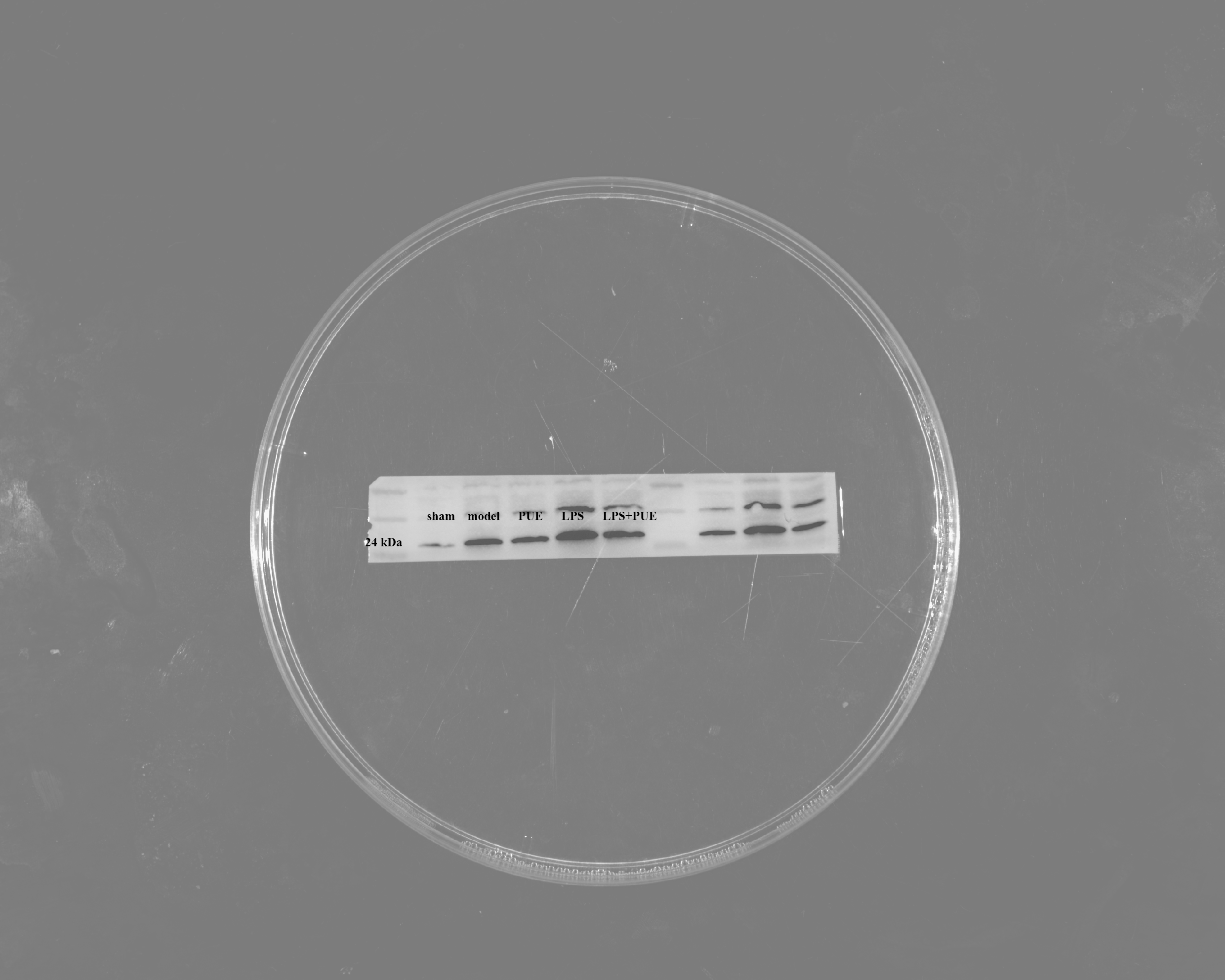

Supplement: Supplementary file 4 [file DataSheet2.ZIP › IL6/IL6 (2).tif]

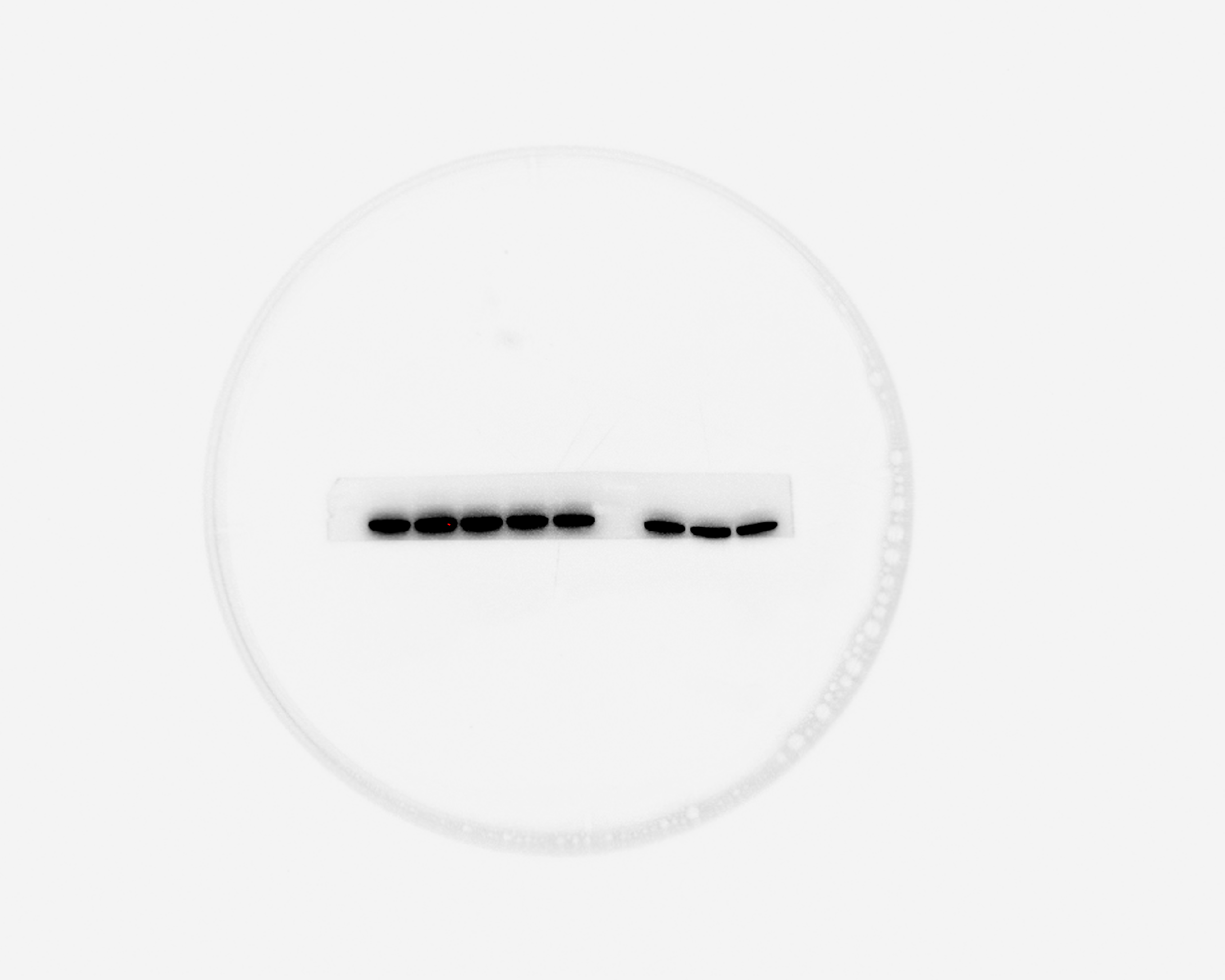

Supplement: Supplementary file 4 [file DataSheet2.ZIP › IL6/β-actin (1).tif]

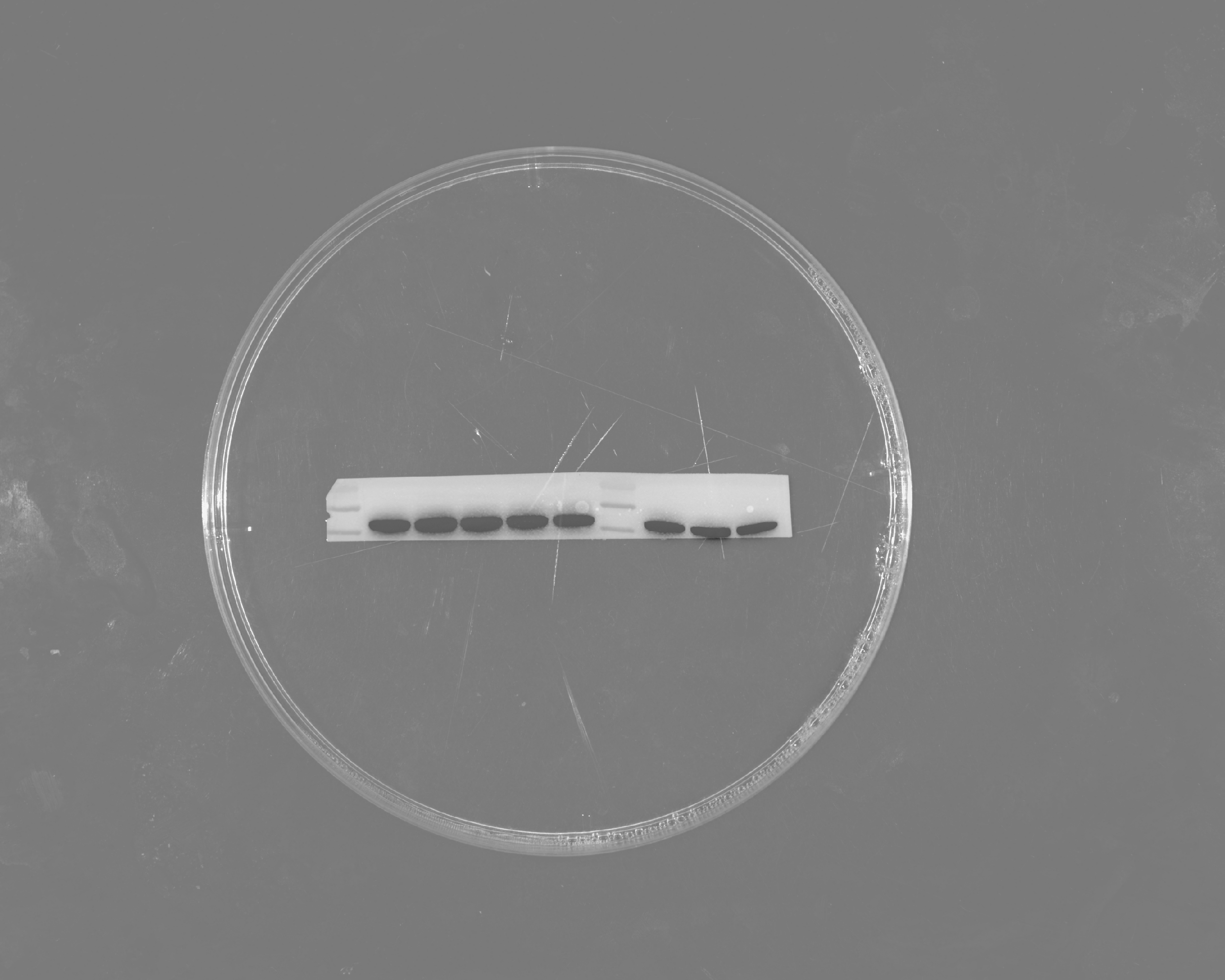

Supplement: Supplementary file 4 [file DataSheet2.ZIP › IL6/β-actin (2).tif]
