# Supplementary material for: Investigating the therapeutic mechanism of Puerarin in vascular dementia: an integrated approach combining network pharmacology and experimental validation
Source: Front Pharmacol. 2026 Apr 29;17:1796295. doi: 10.3389/fphar.2026.1796295 (PMC13168132; doi:10.3389/fphar.2026.1796295)
Supplement: Supplementary file 2 [file Table1.docx]

| **Gene** | **Primer**  **(5′–3′)** | | **NCBI Gene ID** | **Species Specificity** |
| --- | --- | --- | --- | --- |
| ZO-1 | | F: ACCGGAGAAGTTTCGAGAGC  R: TCGGGTTTCCCCTTTGAAGAA | 292994 | *Rattus norvegicus* |
| Occludin | | F: ATAGCCATTGTCCTGGGGTTC  R: TTCCACATTAGGGGGCTGTTC | 83497 | *Rattus norvegicus* |
| Claudin-5 | | F: TACTCAGCACCAAGGCGAAC  R: TTCCCACATCGGTCTTTCCG | 65131 | *Rattus norvegicus* |
| TLR4 | | F: TCAGCTTTGGTCAGTTGGCT  R: TGACACCATTGAAGCTGAGGT | 29260 | *Rattus norvegicus* |
| Myd88 | | F: AGGTGTCCAACAGAAGCGAC  R: AGGACTTGGTGCAAGGGTTG | 25137 | *Rattus norvegicus* |
| TNF-a | | F: ACTGAACTTCGGGGTGATCG  R: GCTTGGTGGTTTGCTACGAC | 24835 | *Rattus norvegicus* |
| IL-6 | | F: ACCACTTCACAAGTCGGAGG  R: TCTGACAGTGCATCATCGCT | 24498 | *Rattus norvegicus* |
| GAPDH | | F: ACGGGAACCCATCACCATC  R: GGTGGTGAAGACGCCAGTAG | 24383 | *Rattus norvegicus* |

Supplementary Table 1
